# Using Structured Decision Making to Evaluate Wetland Restoration Opportunities in the Chesapeake Bay Watershed

**DOI:** 10.1007/s00267-022-01725-5

**Published:** 2022-10-08

**Authors:** David M. Martin, Amy D. Jacobs, Chase McLean, Michelle R. Canick, Kathleen Boomer

**Affiliations:** 1 Maryland/DC Chapter, The Nature Conservancy, 425 Barlow Place, Suite 100, Bethesda, MD 20814 USA; 2 Foundation for Food and Agricultural Research, 401 9th Street NW, Suite 630, Washington, DC USA

**Keywords:** Decision making, Multi-criteria analysis, Non-monetary valuation, Spatial analysis, Water quality

## Abstract

Wetland restoration is an important water quality and climate resilience strategy. Wetland restoration rarely considers tradeoffs at large spatial and temporal scales, which limits capacity to aid decision makers. High resolution data can reveal hundreds to thousands of possible restoration options across a landscape, but guidance for setting restoration targets at these scales is limited. This study uses structured decision making (SDM) as a process for evaluating the desirability of numerous restoration options, with a case study on the Outer Coastal Plain of the Chesapeake Bay watershed, USA. The Nature Conservancy, in partnership with federal, state, and nonprofit organizations, evaluated a decision to target large-scale wetland restoration based on two fundamental objectives: improve water quality and enhance climate resilience. A total of 964 potentially restorable alternatives were delineated across the study area. The alternatives were evaluated on seven water quality and climate resilience criteria. High-priority alternatives were mapped based on multi-criteria ranking methods and principal component analysis. Sensitivity analysis included varying nutrient load data, implementing multiple ranking methods with different assumptions, and varying criteria weights. The maps revealed seven distinct regions of restoration opportunities. Tradeoffs were evaluated to distinguish between desirable and less desirable regions. Results indicated that three regions were promising choices to initiate landowner engagement and outreach. This study highlights the advantages of SDM to structure large-scale restoration decisions. In doing so, our work offers a roadmap toward further developing SDM in future applied restoration contexts.

## Introduction

Ecological restoration aims to recover natural ecosystem functions and achieve ecological, social, economic, and cultural objectives (SER Society for Ecological Restoration International Science and Policy Working Group, 2004). Existing ecological theory has significantly enhanced capacities to understand and study the interactions within and across a diversity of ecosystems and scales. Principles and guidelines for ecological restoration aid decision makers in applying management practices to plan, implement, monitor, and evaluate restorative activities (Gann et al., 2019). However, restoration decisions are often not clarified, especially when they are complex and uncertain and many people are involved, including restoration practitioners, project managers, strategy leads, and local and indigenous communities that manage their own territories (Matzek et al., 2017). Restoration guidance can be improved by methodological research aimed at structuring a deliberate decision-making process, especially in contexts that involve many objectives and tradeoffs.

Limitations in human behavior and judgement can seriously affect the effectiveness of restoration actions and outcomes (Gregory et al., 2012). An intuitive approach to decision making implies that decision makers assign value directly to their options, or *alternatives*, paying attention to only one or a few dimensions of the decision context. There are well-documented issues with this approach, including many forms of cognitive errors and biases (von Winterfeldt and Edwards, 1986; Kahneman, 2013), misunderstanding the value of alternatives (Game et al., 2013), uncertainties in how the alternatives can achieve the objectives (Fischhoff and Davis, 2014), and inabilities to handle many-objective (Bond et al., 2008) and many-alternative situations (Siebert and Keeney, 2015).

*Structured decision making* (SDM) is a proactive approach to better structure a natural resource management decision and subsequent analysis process (Gregory et al., 2012). The foundations of SDM are based on well-established bodies of knowledge and professional practices, including behavioral decision theory, operations research, and systems analysis (Parnell et al., 2013). For the purposes of this article, SDM can be condensed into several inquiries, including (i) clarifying the decision, (ii) clarifying the objectives that will inform the decision, (iii) clarifying the restoration alternatives to choose from, (iv) clarifying how the alternatives will achieve the stated objectives, and (v) evaluating tradeoffs among alternatives. Assumptions, causal models, and decision-relevant preferences that inform these inquiries will vary depending on the decision context (Gregory et al., 2012).

Two key features of SDM are problem decomposition and iteration. Decision analysts work with decision makers to decompose the value of their decision options into different dimensions, subject the dimensions to separate examination, and then apply logic to integrate the dimensions and examine tradeoffs (Keeney and Raiffa, 1993; Gregory et al., 2005). One of the most important contributions to SDM, and one that explicitly targets problem decomposition, is *value-focused thinking* (Keeney, 1992). Value-focused thinking uses values to create and organize objectives and evaluates the consequences of decision options in consideration of those objectives. It is believed that following this process will result in a decision that is a direct reflection of what decision makers care about. These logical concepts, in conjunction with targeted facilitation and elicitation techniques, operationalizes SDM into a prescriptive framework (Gregory et al., 2012). Iteration allows decision makers to rapidly move through the process, achieving some general understanding, and coming back to refine or change previous work as clarifications to the process.

These features of SDM are beneficial for several reasons. First, they make decision makers consult their objectives and preferences seriously so that they can communicate effectively. Second, decision makers can measure the value of each alternative on a set of standards, or *criteria*, in same or similar ways, and then combine them into an overall value or “worth” for each alternative. Third, tradeoffs can be adequately evaluated using quantitative or qualitative techniques, such as assigning relative weights to the variation of criteria or openly discussing the differences among alternatives and their corresponding criteria. These features have been found to improve decision-making skills and decision-maker satisfaction in empirical (Arvai and Gregory, 2003; Hostmann et al., 2005; Siebert et al., 2021) and reflective analyses (Keeney et al., 1990; Bessette et al., 2019; Martin 2021).

## Literature Review

The application of SDM to environmental management is relatively new (Gregory et al., 2012). Recent reviews show growing support for this approach in many different decision contexts, including spatial analyses (Huang et al., 2011; Langemeyer et al., 2016; Cegan et al., 2017). Some recent restoration studies have focused specifically on developing objectives based on input from interested parties (Guerrero et al., 2017), developing alternative restoration scenarios (Singh et al., 2019), developing and evaluating tradeoffs quantitatively (Failing et al., 2013; Martin et al., 2018), as well as developing methods that warrant adaptive management (Lyons et al., 2008; Peterson and Duarte, 2020).

This study applies SDM to evaluate and prioritize a discrete set of wetland restoration alternatives across a large landscape. This approach is similar to the so-called reserve design problem, where a minimum set of spatial planning units are selected from a larger feasible set of options to meet biodiversity targets, among other objectives (Pressey et al., 1993). Many studies use mathematical programming to “design” the number of possible planning units based on a multi-objective function subject to quantitative factors that constrain the search for optimal alternatives (e.g., complementarity, irreplaceability; Possingham et al., 2000). This strictly analytical decision process has been widely employed across the world but is limited in regard to its applicability. Some reviews draw attention to the lack of non-quantitative and context-specific factors included in many decision situations, the lack of co-designed planning products that explicitly and transparently reveal decision maker assumptions, and possible confusion about whether data and outputs are used in the final decision-making process (Knight et al., 2006; Knight and Cowling, 2007; Tani and Parnell, 2013).

We take a more deliberative and hands-on approach to assist decision makers in the search for restoration planning units where constraints and tradeoffs are respectively applied and evaluated directly by decision makers. Our approach more closely resembles spatial opportunity mapping in that we aim to understand the many factors that may lead to action and incorporate them into the selection of areas where action is suitable for implementation (Knight et al., 2010). By doing this, resources may be allocated for targeted outreach to facilitate wetland restoration in these areas. Many studies have used opportunity mapping to identify high-priority planning units (e.g., Nelson et al., 2009; Jellinek, 2016), but applied studies using SDM are needed to demonstrate broad applicability and relevance toward effectively clarifying assumptions and preferences and promoting user input in a spatial planning process.

## Case Study

The Chesapeake Bay is the largest estuary in the United States, and its watershed has some of the highest rates of coastal population growth and sea-level rise (Bilkovic et al., 2019). The watershed covers over 165,000 km^2^ and seven jurisdictions: Delaware, Maryland, New York, Pennsylvania, Virginia, West Virginia, and District of Columbia. Like many other coastal systems around the world, the estuary faces serious water quality challenges threatening fisheries, aquaculture, and recreational economies. Eutrophication has been driven by human-induced nitrogen, phosphorus, and sediment runoff, contributing to algal blooms and hypoxia in the estuary and its tidal tributaries (Kemp et al., 2005). The jurisdictions are currently under a 2010–2025 mandate to reduce nutrient and sediment and restore water quality standards for dissolved oxygen, chlorophyll a, water clarity, and underwater grasses (USEPA, 2010). Agricultural land uses comprise 19% of the watershed and contributes 48% of the nitrogen to the estuary, making it an area of focus for research and implementation (Kleinman et al., 2019). The challenge to meet the mandate is complicated by the impacts of climate change including increased frequency of heavy rain events and intervening drought periods, leading to the need for greater water storage capacity and assisted habitat and species migration (Trenberth, 2011).

**Fig. 1:**
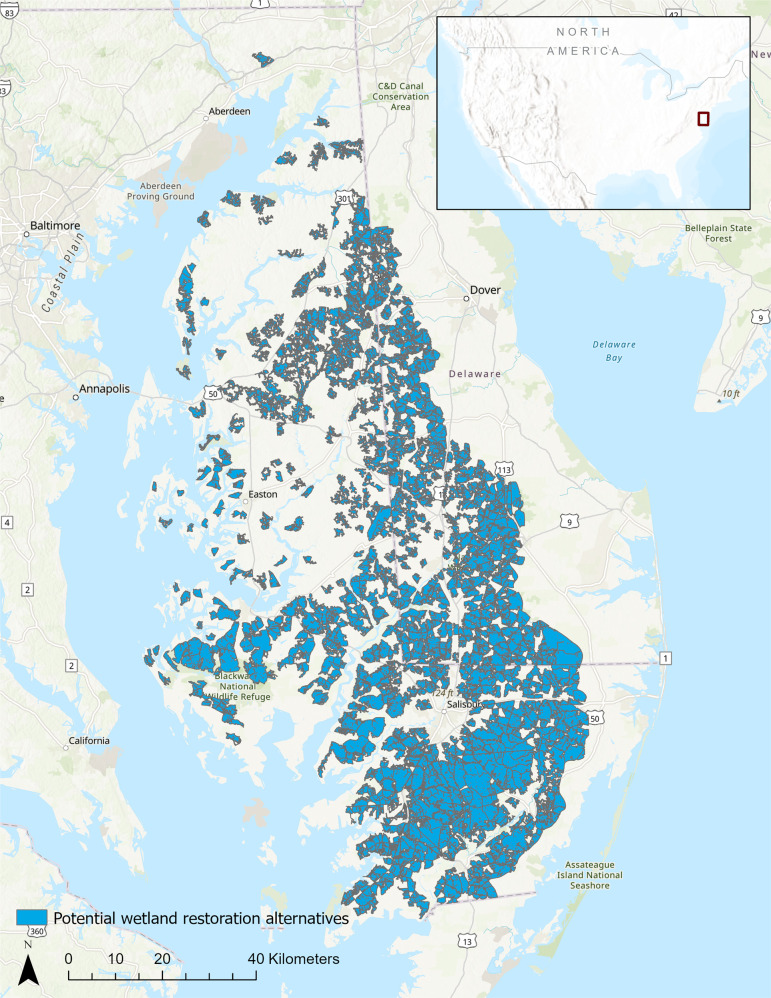
Potential wetland restoration alternatives on Delaware and Eastern Shore of Maryland portions of the Chesapeake Bay watershed. The alternatives are polygons that meet several screening criteria for a potentially restorable area, including contiguous areas of natural and agricultural land use, size, and predicted water table depth (Supplementary material)

A team at The Nature Conservancy—a global conservation organization—implemented a decision analysis, including facilitation support from the lead author, spatial analysis and methodological support by the co-authors, and decision-making support from a multi-agency partnership between The Nature Conservancy and U.S. Department of Agriculture Natural Resources Conservation Service, U.S. Fish and Wildlife Service, Maryland Department of Natural Resources, and Ducks Unlimited. The partnership developed a decision statement that guided the research: Where is the greatest opportunity for large-scale wetland restoration in the study area? The decision statement is purposefully broad and may require several layers of important decisions at different scales of analysis. The study area was the Delaware and Eastern Shore of Maryland portions of the Chesapeake Bay watershed (Fig. 1) because of its high concentration of agricultural land uses and high levels of wetland densities (Cheng et al., 2020) and losses (Tiner and Finn, 1986).

The decision context presented in this study included seeking potentially restorable areas that were greater than 300 acres in size and with opportunity to improve water quality and climate resiliency. Wetlands play a critical role in mitigating water quality impairments and human- and climate-induced weather events, leading to enhanced biodiversity and human well-being (Mitsch and Gosselink, 2000; Brander et al., 2006). Wetland restoration that is downgradient of agricultural lands provides a natural filter for nutrients and sediment and thus represents an important strategy to improve water quality (Woltemade, 2000; Hansen et al., 2018; Jordan et al., 2003; O’Green et al., 2010). Restored wetlands also reduce risks from drought or flooding by increasing terrestrial water storage (Arkema et al., 2013). However, not all wetlands provide the same functions, and meeting Chesapeake Bay goals require a targeted approach to identify which locations can provide the greatest and most reliable opportunities.

This research focused on biophysical targeting of possible wetland restoration and rehabilitation in agricultural landscapes where there are opportunities to restore marginal cropland and improve the function of degraded wetlands in forests. Our focus assumes that resource allocation efficiencies are in landscape-scale restoration. Site-scale studies or restoration-specific analyses tend to be extremely variable (Jordan et al., 1997), and the partnership wanted to screen large contiguous areas where wetland restoration can remove as much pollution as possible, so that landowner engagement and outreach can be more targeted.

In the remaining sections, we outline and describe an SDM process. Objectives and criteria for evaluating possible wetland restoration options were identified through elicitation and facilitation. Large-scale restoration alternatives were identified through spatial analysis. Multi-criteria ranking methods and principal component analysis modeled the strength-of-preference value of the alternatives. High-priority alternatives were identified and further screened for tradeoffs across the landscape.

## Methods

### Objectives and Criteria

Objectives form the basis from which possible decision alternatives can be identified and compared. In many consultations between the authors and partnership members, two objectives were identified to improve water quality and climate resilience in the study area (Table 1). The water quality objective was further clarified with sub-objectives regarding nutrient load inputs, agricultural sources, and wetland function, whereas the climate resilience objective was further clarified into sub-objectives regarding water storage capacity and species movement.

**Table 1:** Objectives and criteria for evaluating wetland restoration alternatives

Objective	Sub-objective	Criterion (measure)	Preferred direction	Reference
Water quality	Nutrient load inputs	Total nitrogen (kilograms/yr)	Increase	Chesapeake Assessment Scenario Tool
		Total phosphorus (kilograms/yr)	Increase	Chesapeake Assessment Scenario Tool
	Agricultural sources	Proportion of contributing land use area (percent)	Increase	Chesapeake Conservancy, 2013/2014 High-Resolution Land Use and U.S. Department of Agriculture 2019 Cropland Data Layer
	Wetland function	Proportion of ecoydrologically active area (percent)	Increase	The Nature Conservancy
		Presence/absence of floodplain	Yes/No	Flood Emergency Management Agency, 100-Year Effective Floodplain
Climate resilience	Water storage capacity	Proportion of hydrologic soil groups with high storage potential and residence time (percent)	Increase	Natural Resources Conservation Service (SSURGO)
	Species movement	Proportion rated “above average” climate flow (percent)	Increase	Anderson et al. (2016)

See Supplementary material for more information on each criterion

It is important to note here the nuances of using value-focused thinking to facilitate conversations from knowledgeable participants. Answers to general questions like “what are you aiming to achieve” are often unclear and messy. Two facilitation techniques were used to elicit the objectives and sub-objectives. First, it is often the case that decision makers have a hard time separating means from ends and need some procedural aid in converging on the fundamental objectives to guide the decision (Bond et al. 2008). In this study, we asked questions like “why is that important” to converge on the water quality and climate resilience objectives. Second, objectives can have multiple meanings in different decision contexts, and they often need to be specified to be clearer and more transparent about what needs to be achieved, so that causal factors and alternatives can directly contribute to desired changes (Keeney, 2007). The water quality and climate resilience objectives were viewed as a model of multiple decision-maker values (Keeney and von Winterfeldt, 2007), and we asked questions like “what do you mean by that” or “what aspects of that objective are important” to clarify the sub-objectives.

Seven criteria were developed as performance measures for the sub-objectives. This study was constrained by the information that could be gathered at the scale of analysis. Criteria selection was informed by deliberative facilitation techniques that included asking unexpected hypothetical questions to challenge thinking. Questions like “if all else were equal, would you choose one of two options based on differences in this measure” helped to elucidate possible criteria measures, as well as reviews of published reports, input from the partnership, and spatial data from regional organizations.

Decision maker preferences for evaluating the decision were to prioritize restoration alternatives with the highest criteria values, assuming that these amounts provided the best opportunity or value to achieve the objectives through restoration (Table 1, column 4). This approach to defining objectives and meaningful criteria is different than identifying all possible causal factors or complex ecological relationships between causal factors. Rather, this approach was implemented to distinguish the desirability of decision options as conveyed by decision makers. A brief overview of the criteria is provided in this section with more detailed information in the Supplementary material.

Total nitrogen (TN) and total phosphorus (TP) refer to the modeled amounts of nutrient loads entering a potentially restorable alternative from contributing land use sources. To measure these criteria, a watershed was delineated for each potentially restorable alternative using the Batch Watershed Delineation for Polygons program from the Esri Arc Hydro toolkit (ArcMap 10.6). Within each of the delineated watersheds, a land use analysis was performed using the Chesapeake Conservancy’s 1-meter High Resolution Land Use Dataset (www.chesapeakeconservancy.org), enhanced with additional areas of cropland from the United States Department of Agriculture Cropland Data Layer. The area of three land use sectors—agriculture, developed, and natural—were calculated within each delineated watershed. These values were used as inputs for the loading rate calculations.

Loading rate estimates can be highly variable and depend on model assumptions. Two different TN and TP loading rates for each land use sector were gathered from the Chesapeake Scenario Assessment Tool web portal (https://cast.chesapeakebay.net/). The loading rate estimates are based on different model scenarios, assuming existing or future nutrient-removing practices across the Chesapeake Bay watershed. The 2019 Progress scenario, hereafter 2019 scenario, assumed loading rates based on practices that are currently implemented across the watershed through 2019. The Phase III Watershed Implementation Plan scenario, hereafter 2025 scenario, projected loading rates based on future implementation of practices across the watershed through 2025. The six different loading rates, three for each land use sector per scenario, were then multiplied by the acres of each land use sector found in each watershed per alternative and converted to kilograms per year. This process resulted in two sets of TN and TP values per alternative and were used to understand how sensitive the results were to differences in loading inputs.

Targeting restoration in places with the opportunity to filter nutrients from agricultural areas was important to decision makers. The area of three land use sectors, as calculated above, were used to calculate a proportion of agricultural land use (AG) criterion. AG was calculated as the percent of total land use in the contributing watershed per alternative. Agricultural lands are the dominant managed land use in the region and were used to prioritize alternatives with anthropogenic sources of nutrients rather than from natural sources, such as forest and wetlands.

Ecohydrologically active areas (EHA) are areas across the landscape where there is the potential for interaction between the plant rooting zone and the groundwater table, and thus where wetland biogeochemical processes are more likely to occur and influence water quality through effects on nutrient dynamics. The only required model input is high-resolution, hydrologically-enforced topography data (LiDAR-derived). EHA were mapped using the hydrology toolbox, using a script that drew on widely available tools across geospatial software packages to analyze these data. Stream networks were derived using the single-flow direction (D8) algorithm in ArcGIS Pro and applied to 1-meter resolution topography data. Relative elevations were calculated as a grid by attributing each pixel with the elevation of the nearest surface water feature located along a downstream flowpath, and then subtracting that elevation from the focus cell value. EHA were measured as land areas within 0.5-meter elevation of the nearest down-gradient surface water feature, per alternative (Murphy et al., 2008; Jencso et al., 2009).

Floodplains (FP) have a high retention rate for nitrogen and phosphorus pollutants (Gordon et al., 2020). The presence or absence of the Federal Emergency Management Agency’s effective 100-year floodplain was identified inside each alternative. This criterion’s binary feature was primarily important in the search for desirable alternatives.

Wetlands can safeguard against climate-induced vulnerabilities such as variations in precipitation and surface water runoff (Pörtner et al., 2022). We estimated water storage capacity (WS) based on hydrologic soil groups, which are characterized by storage potential and residence time. Dual class hydrologic soil groups classified as A/D are sandy and have high storage potential but low residence time. Groups classified as B/D are loamy and have good storage potential and moderate residence time. Groups classified as C/D are fine textured and have low total storage potential but high residence time. This criterion mapped and calculated the proportion of land that was designated hydrologic soil classifications A/D, B/D, and C/D per alternative, using the SSURGO database, as suggested by the state soil scientist at the Natural Resources Conservation Service for Maryland and Delaware.

The climate flow (CF) criterion measures direction and intensity of plant and animal movement or “flow” patterns across the region in response to climate change (Anderson et al., 2016). Areas of high climate flow have less resistance to species movement, more natural cover, and are weighted toward important climate gradients. Climate flow was calculated using a landscape permeability model based on anthropogenic resistance and climate gradient factors (Anderson et al., 2016). This criterion mapped and calculated the proportion of land that was designated “above average” climate flow per alternative.

### Restoration Alternatives

Under a value-focused thinking approach to SDM, decision alternatives are typically developed as a response to objectives and criteria (Gregory et al., 2012). We took a hybrid approach in this study and developed the alternatives slightly independently of the objectives and criteria because the alternatives had to be based in part on spatial information that could be gathered at the scale of analysis. For this reason, spatial analysis was used to delineate 964 potential restoration alternatives (Fig. 1). The team extracted and mapped agricultural land use (excluding productive agriculture on prime farmland soils) and natural land cover (including upland and wetland habitat, excluding tidal wetlands) across the analysis area, primarily using the Chesapeake Conservancy’s 1-meter High Resolution Land Use Dataset. Potential wetland restoration alternatives consisted of contiguous landscape patches of the mapped agricultural land use and natural land cover that were at least 121 hectares (300 acres) in size and contained at least 61 hectares (150 acres) of ecohydrologically active area (as calculated to develop that criterion; see above). See the Supplementary material for a stepwise procedure for delineating the alternatives in ArcGIS Pro.

### Multi-criteria Ranking

The spatial analysis resulted in criteria measures for the 964 alternatives (Tables S1, S2). This is a very large multivariate dataset that requires some evaluation and sensitivity analysis to distinguish desirable from undesirable options in a prioritization or ranking sense.

Multi-criteria ranking methods model decision maker preferences and value judgements to infer a rank ordering of alternatives. Two ranking methods were implemented to account for sensitivity in aggregating criteria: a compensatory value function based on multi-attribute value theory (Dyer and Sarin 1979) and a non-compensatory distance function based on compromise programming (Zeleny 1974). Compensation refers to whether good criteria compensate for poor criteria when they are aggregated (Martin and Mazzotta 2018). Each method involved two modeling assumptions. First, criteria measures were transformed to a common strength-of-preference scale while maintaining their differentiation across the alternatives, so that they could be combined. Second, weights were assigned to the criteria, so that the scaled values could be aggregated across the criteria into a single expected value per alternative.

A value function represents the value of a single alternative on the set of criteria. The functional form of the value function takes each criterion measure *z*_*ij*_ and transforms it to between 0 and 1 using a scaling function *x*_*ij*_ (e.g., linear):

1 $$x_{ij} = \frac{{z_{ij} - z_j^ \ast }}{{z_j^{ \ast \ast } - z_j^ \ast }}$$

for all criteria *j*, alternatives *i*; where $z_j^ \ast$ and $z_j^{ \ast \ast }$ are the “worst” and “best” values for each criterion across alternatives, respectively. In this case, higher values are more desirable than lower values. We used the value function *V*_*i*_ to aggregate the scaled criteria into an overall value per alternative *i*:

2 $$V_i = \mathop {\sum }\limits_{j = 1}^7 w_jx_{ij}$$

where *w*_*j*_ are weights that sum to one.

A distance function represents the distance between a single alternative and an ideal but non-feasible alternative in geometric space. The functional form of a distance function takes each criterion measure *z*_*ij*_ and transforms it to between 0 and 1 using a scaling function *x*_*ij*_ (e.g., linear):

3 $$x_{ij} = \frac{{z_j^{ \ast \ast } - z_{ij}}}{{z_j^{ \ast \ast } - z_j^ \ast }}$$

for all criteria *j*, alternatives *i*; where $z_j^ \ast$ and $z_j^{ \ast \ast }$ are the “worst” and “best” values for each criterion across alternatives, respectively. In this case, lower values are more desirable than higher values because decision makers seek to minimize the distance between each criterion measure and its ideal or maximum measure. We used the distance function *D*_*i*_ to aggregate the scaled criteria measures into an overall distance per alternative *i*:

4 $$D_i = \mathop {\sum }\limits_{j = 1}^7 w_j^px_{ij}^p$$

where *w*_*j*_ are weights that sum to one; *p* is a distance or *L*_*p*_ norm in geometric space. Distance norm values of 1 ≤ *p* ≤ ∞ weight deviations from the best value higher with greater distance, which controls the level of compensation (Euclidean distance is commonly used, where *p* = 2).

We considered both linear and non-linear scaling functions based in part on the frequencies of criteria measures across the alternatives (Figs. S1, S2). Risk neutrality implies that the strength-of-preference scale is linear as in Eqs. (1) and (2), risk aversion implies that the scale is concave, and risk proneness implies that the scale is convex. These scales can be approximated using elicitation procedures that may include direct probability judgements based on relevant beliefs in a proposition (e.g., frequencies, logical analysis, expert opinion), or indirect judgments based on hypothetical gambles with fixed or varying probabilities (von Winterfeldt and Edwards, 1986). We reviewed the frequencies and determined that less frequent and very large criteria measures for criteria TN, TP, and CF were less important and should not dominate the overall desirability of the alternatives. This risk-averse attitude means that preferences were marginally decreasing for less-frequent and very large criteria measures. Therefore, a 0-1 non-linear risk-averse scale was assigned to those criteria using their cumulative density functions, or the probabilities that an observed value was less than or equal to a criterion measure. The shapes of the cumulative density functions of criteria TN, TP, and CF were largely concave, corresponding to risk aversion (Figs. S3, S4). These scaled values were used as input into Eqs. (2) and (3) for the value and distance functions, respectively. For the remaining criteria AG, EHA, FP, and WS, we assumed a linear risk-neutral scaling function and implemented Eqs. (1) and (3) for the value and distance functions, respectively.

Weights are parameters in the value and distance functions that measure the contribution of each single-criterion value to the expected value per alternative (Dyer and Sarin, 1979). There are many ways to elicit criteria weights depending on the preference scales used in the elicitation, such as ordinal, interval, and ratio scales (von Winterfeldt and Edwards, 1986; Danielson and Ekenberg, 2016). Recent research has revealed potential decision-maker confusion when eliciting weights in a hierarchical sense with objectives and criteria (Bessette et al. 2019; Martin, 2021). For this reason and based on decision maker preferences, sensitivity analysis included water quality and climate resilience weighting scenarios to emphasize both objectives. For each weighting scenario, a higher proportion of weight was given to an objective in seven sensitivity iterations, between 60 and 90% in increments of 5%. To illustrate, one iteration of the water quality weighting scenario assigned a weight of 60% to the five water quality criteria (each apportioned weight was 0.6/5 = 0.12). The remaining 40% was then apportioned between the two climate resilience criteria (each apportioned weight was 0.4/2 = 0.2). Seven sensitivity iterations were performed per weighting scenario per ranking method.

### Principal Component Analysis

We previously implemented principal component analysis to understand the underlying structure of the criteria and alternatives (Martin et al., 2022; general steps are in the Supplementary material). The functional form of a principal component *Z*_*ni*_ takes each criterion measure *Z*_*ij*_ and transforms it to between −1 and 1 using a linear scaling function *x*_*ij*_:

5 $$x_{ij} = \frac{{z_{ij} - \overline {z_j} }}{{s_j}}$$

for all criteria *j*, alternatives *i*; where $\overline {z_j}$ is criterion mean and *s*_*j*_ is its standard deviation. The first principal component *Z*_1*i*_ aggregates the scaled criteria values into an overall value, sometimes referred to as a Z-score, per alternative *i*:

6 $$Z_{1i} = \mathop {\sum }\limits_{n = 1}^l v_{n1}x_{ij}$$

where coefficients *v*_*n*1_ are elements of the eigenvector associated with the dominant eigenvalue *λ*_1_ from covariance matrix *A* (Supplementary material).

That analysis spread the alternatives onto a plane of maximum variation in TN, which represented 99% of the variation in the data set. The linear combination of principal eigenvalues and standardized criteria (*Z*_1_-scores) were consistent with inherent tradeoffs between criteria EHA, WS, CF, and sometimes AG, and criteria TN and TP (Martin et al., 2022). These tradeoffs were associated with a specific preference structure for the nutrient loading criteria, without requiring the more difficult task of assessing strength-of-preference values or weights using ranking methods (Martin et al., 2022). Decision makers were willing to consider this assumption, and we organized the alternatives in accordance with the lowest *Z*_1_-scores associated with the principal component (Supplementary Material from Martin et al., 2022).

### Mapping and Tradeoffs

Maps were developed to visualize and prioritize alternatives based on the ranking methods and principal component analysis. Decision makers desired many options for engagement and outreach specialists to consider across the landscape, and iteration and best professional judgment resulted in the assumption to map at least 10% of the 964 potentially restorable wetlands. We selected the top 100 ranked alternatives per weighting scenario, as averaged by their value and distance functions over seven sensitivity iterations. Alternatives considered to be water quality priorities included the non-overlapping alternatives that ranked in the top 100 under the water quality weighting scenario as well as the 100 alternatives with the lowest *Z*_1_-scores. Alternatives considered to be climate resilience priorities included the non-overlapping alternatives that ranked in the top 100 under the climate resilience weighting scenario. Alternatives considered to be highest priority included the overlapping alternatives that ranked in the top 100 under all weighting scenarios. Alternatives that fell into these three categories were mapped. Two maps were developed corresponding to the different nutrient loading data scenarios (see above). We annotated the maps to identify clusters of planning units of potentially restorable areas to perform targeted outreach. Tradeoffs among the hand-picked units, hereafter referred to as regions, were evaluated using consequence tables.

## Results

The methods applied in this study identified many water quality and climate resilience priorities in the study area. These priorities were informed by a deliberative and iterative SDM process that focused on spatial data inputs, preferences and judgments, and sensitivity analysis. Unless otherwise noted, we present results from implementing the methods using data from the 2025 nutrient loading scenario because there was no significant variation in the results based on the different loading inputs.

### Priority alternatives

The ranking and mapping effort resulted in several hundred priority alternatives out of a total of 964 alternatives identified across the study area. According to initial results using the 2025 nutrient loading scenario, 106 alternatives were identified as water quality priorities, 76 alternatives were identified as climate resilience priorities, and 40 alternatives were identified as priorities under both objectives. These results are consistent with the 2019 scenario, with the exception of slightly fewer climate resilience priorities identified, slightly more water quality priorities identified, and slightly more priorities identified under both objectives.

**Fig. 2:**
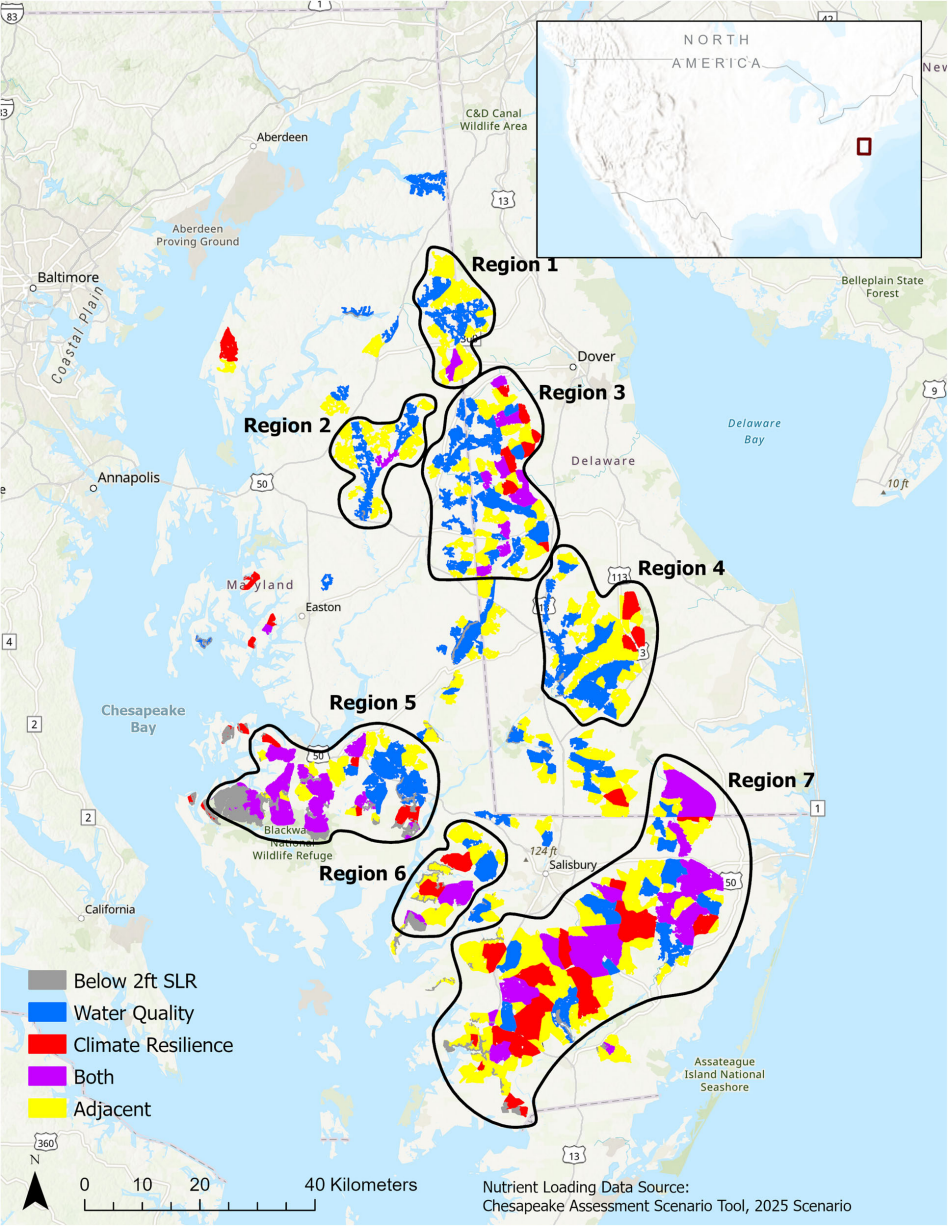
Priority alternatives across the study area under 2025 Scenario

After initial reviews of the results, we annotated the map by assigning three additional constraints based on organizing tasks and leveraging partnership investments in putting restoration on the ground. First, decision makers wanted to consider the potential connectivity and added benefit of restoration between priority alternatives across the landscape. Therefore, alternatives adjacent to a priority alternative were mapped. Second, they wanted to exclude alternatives below an elevation of two feet relative to mean sea level, as these areas are currently undergoing conversion to tidal wetlands with sea level rise and ineligible for enrollment in many conservation easement programs. Third and based on the previous two constraints, we identified regions of priority alternatives for further planning and analysis. These assumptions resulted in a final list of 184 priority alternatives in seven regions under the 2025 scenario (Fig. 2) and 180 alternatives in the same seven regions under the 2019 scenario (Fig. S5), not accounting for priority alternatives outside of the regions or adjacent alternatives inside of the regions.

### Tradeoffs

**Fig. 3:**
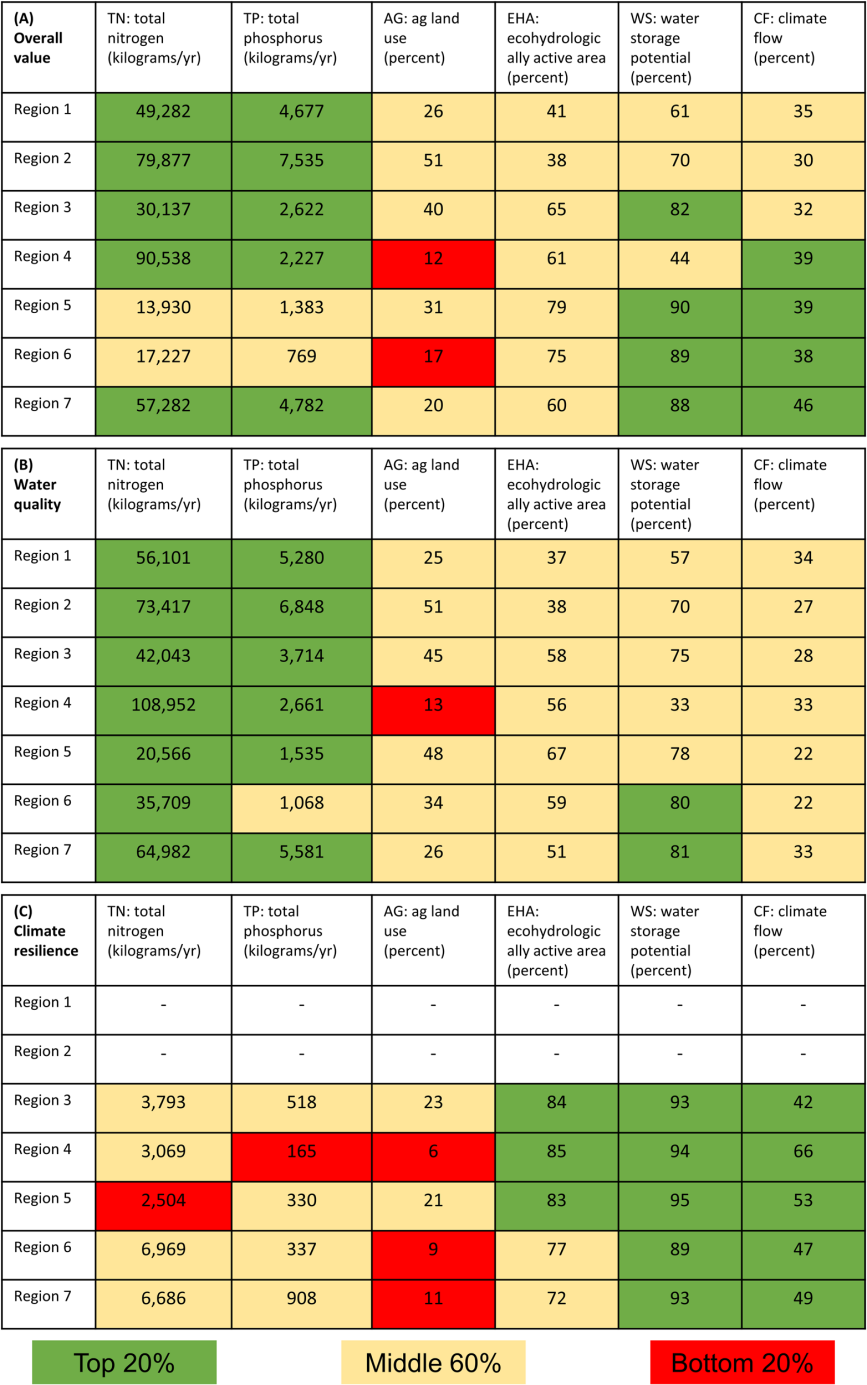
Consequences table for tradeoff analysis. Consequence table revealing tradeoffs among regions and their priority alternatives regarding **A** overall criteria measures (both water quality and climate resilience priority alternatives), **B** water quality values, and **C** climate resilience values. Regions 1 and 2 did not contain any priority alternatives for climate resilience and were therefore not analyzed in (C)

Tradeoffs were evaluated to distinguish the desirability of the regions for targeted landowner outreach. The average criteria measures of priority alternatives inside each region were used to construct consequence tables (Gregory et al., 2012) regarding potential water quality, climate resilience, and overall value (*value* in terms of average criteria measures) in each region (Fig. 3). Overall value regarded priority alternatives for water quality, climate resilience, and both water quality and climate resilience. A two-step process simplified the consequence tables. First, the average measures were color-coded based on their placement in the upper 20%, middle 60%, and lower 20%, while considering that preferences were linear for some criteria (AG, EHA, WS) and nonlinear for others (TN, TP, CF). This step placed a value judgment on the ranges of average criteria measures and allowed elimination of potentially irrelevant criteria whose values were similar across the regions. Second, the dominance concept was used to evaluate the regions based on their color-coded values. *Dominance* refers to elimination of less desirable regions that are worse than others on at least one criterion and the same as or worse on the remaining criteria. Regions that are not worse or better over the criteria, as evaluated by their color-coded placement, are non-dominated and therefore worthy of further consideration because they trade off value between criteria. In other words, regions identified as dominated were outperformed by other regions over all criteria, regions identified as non-dominated traded off value on some but not all criteria and are therefore desirable, and regions that dominated all other regions are the most desirable.

According to overall value, Region 7 dominated Regions 1, 2, 3, 4, 5, and 6 (Fig. 3A). To illustrate, Region 7 dominated Regions 1 and 2 because it was equally as valuable over criteria TN, TP, AG, and EHA and higher in value over criteria WS and CF. In fact, the EHA criterion was considered to be irrelevant because its value was similar across all regions. Though this criterion was an important attribute of water quality, we found no significant differences among any of the regions for that criterion (Fig. 3A), and therefore it was not helpful in distinguishing the desirability among the regions. Likewise, Region 7 dominated Regions 3, 4, 5, and 6 because it was equally as valuable for some criteria but higher in value for the others. To summarize, there were no instances where Region 7 was worse in value as compared to another region and some instances where Region 7 was better in value in comparisons to another region, as evaluated by their color-coded placement.

We considered other instances of dominance. For example, Region 3 dominated Regions 1 and 2 because it was equally as valuable over criteria TN, TP, AG, EHA, and CF and higher in value over criterion WS. Region 5 dominated Region 6 according to this logic as well. In summary, Region 7 was identified as a desirable option, and Regions 1, 2, and 5 were less desirable options in the analysis of overall value.

According to water quality value, Region 7 dominated Regions 1, 2, 3, 4, 5, and 6, and criteria TN and EHA were found to be irrelevant (Fig. 3B). Regions 1, 2, 3, and 5 were all similar regarding both water quality and climate resilience value. These regions dominated Region 4 (lower AG value) but traded off value in comparison to Region 6 (higher WS value but lower TP value). In summary, Region 7 was a desirable option and Region 4 was a less desirable option in the analysis of water quality value.

According to climate resilience value, Region 3 dominated Regions 4, 5, 6, and 7, and criteria WS and CF were found to be irrelevant (Fig. 3C; there were no priority alternatives for climate resilience in Regions 1 and 2). Regions 4, 5, 6, and 7 were non-dominated because they all traded off value in certain criteria. In summary, Region 3 was a desirable option in the analysis of climate resilience value.

## Discussion

This study used SDM to evaluate a decision of where to target landowner engagement and increase wetland restoration across a large landscape based on water quality and climate resilience interests. The analysis introduced clarity and rigor based on its ability to break down the decision into related parts: specifying value-focused objectives and criteria, specifying potentially restorable wetland alternatives, and integrating preferences and analytical methods to evaluate the alternatives on the set of criteria.

After careful deliberation, which included some of the above iterations based on viewing preliminary results, the multi-agency partnership made a decision to perform initial outreach and engagement in Regions 3, 5, and 7 across the study area. Although Region 5 did not dominate all others on an objective, it was not dominated outright over both objectives and there were many high-priority alternatives for both objectives (Fig. 2). An additional insight discussed among the partnership was that Region 5 is currently an area of active engagement, which made it a promising option to better target that engagement. These insights did not have as much to do with the tradeoff analysis as it did with a broader view of the mapping effort. The partnership was reassured in this decision as the analysis recorded assumptions in a clear and easy-to-communicate process, which guarded against implicit biases (von Winterfeldt and Edwards, 1986), emphasized a value-focused approach to facilitation and gathering (spatial) data and other relevant information (Keeney, 1992), and incorporated variation and uncertainty in the values, data, and preferences used in it (Gregory et al., 2012). In summary, SDM reduced the likelihood that personal or organizational biases influenced the deliberations.

The decision has informed further targeted outreach and engagement decisions. The regions that were decided have been divided among the partnership agencies to initiate feasibility studies, including outreach and behavioral research to analyze landowner interest in wetland restoration on their properties. A sub-team of the partnership is currently focusing on developing a working knowledge of individual landowner views, knowledge about, and interest in pursuing wetland restoration on their properties. They are implementing behavior change interventions (Richenback et al., 2017) to initiate engagement and discuss landowner eligibility for restoration programs in each priority region. As mentioned earlier, outreach specialists are currently working in Region 3 and actively responding to farmer and farm landowners interest in restoration opportunities.

This study has focused primarily on revealing a set of assumptions expressed through an organizational decision process. It is important to note the nuances in carrying out this type of process and how different assumptions could lead to different outcomes. The easiest illustration to this effect is that different interests matter to different people. As mentioned earlier, the mapping and tradeoff exercises led to different insights among the partnership. Likewise, we recognize that a different set of objectives and criteria may be identified by other decision-making groups to clarify what matters in a large-scale restoration planning decision. Other interests could be valuable for selecting restoration opportunities in other contexts, such as social benefits (Martin et al., 2018) or carbon sequestration (Villa and Bernal, 2018). We emphasize the importance of decision makers and decision-making groups, including interest groups and other potential project partners, actively participating in the analysis and making assumptions explicit. This can be developed in the presence or absence of a designated decision facilitator, but we think the facilitator is a critically important link between decision makers and available information. Facilitators must make use of best elicitation practices and techniques, including but not limited to ones that deliver value-focused information (Keeney, 1992), and they should consider navigating the process in a way that does not inject their own biases or contribute to the content of the discussion (Phillips and Phillips, 1993).

Additionally, the assumptions are subject to further research to better understand conditions across the study area. For example, abilities to estimate the water storage criterion may improve beyond the soils data used at the time of the analysis. Future iterations on the decision can reveal other methods to evaluate water storage more accurately, such as estimating volume of potential water storage per alternative. These data were not available at the scale of the decision considered. Likewise, other data and uncertainties that were not part of the study as presented could change the analysis. SDM, however, is indifferent to varying data and assumptions, which makes the approach robust to different environmental management contexts.

Structured decision making cannot guarantee that all important factors are included in the analysis. Rather, it aims to concentrate thinking on the more difficult aspects of a decision. To illustrate, ranking methods can streamline the process of evaluating numerous options on many objectives. Ranking methods can utilize simple or rigorous approaches as needed, and there are advantages and disadvantages to each approach (Martin and Mazzotta 2018; Martin, 2021). SDM aims to elicit relevant input from decision makers in a meaningful manner, so that participants’ assumptions and modeling results can be discussed openly and honestly. Similarly, SDM allows decision makers to provide a consistent rationale about tradeoffs. The case study used a consequence table and tradeoff rules to simplify that process, but other tools and assumptions could be useful in other situations (Bower et al., 2018). The key takeaway is that gathering and presenting information in a structured and transparent manner can go a long way toward improving decision makers’ trust in the analysis (Arvai and Gregory, 2003). These and other features can benefit restoration planning even when there are conflicts between ecological and social costs and benefits (Gann et al., 2019). Revealing and reflecting on these assumptions could be an emphasized in future wetland restoration research moving forward.

## Conclusions

The SDM process presented in this article used methods that were logically consistent with decision-maker preferences. In practice, they incited thoughtful, open, and reasonable deliberation among decision makers. This approach can yield better results than intuition-driven decision-making approaches (Arvai et al., 2001; McDaniels, 2019), but we recognize that any decision may or may not lead to good outcomes. On the contrary, SDM can improve peoples’ ability to think strategically in complex situations, which is a critical building block to achieving better outcomes. In general, the methods applied enhanced a shared understanding and satisfaction with what the partnership was trying to achieve. The analysis supported many forms of learning, in particular: (i) what values and objectives were important determinants to the decision; (ii) technical aspects of the ecological systems and how they related to decision-relevant objectives; and (iii) improved transparency and relationships building within the planning team and with partners. These conclusions are reflective because no structured survey or experiment was included to evaluate SDM, but this is an area of future research.

## Supplementary Information

Electronic Supplemental Material

Electronic Supplemental Material

Electronic Supplemental Material
