# Supplementary material for: Using Structured Decision Making to Evaluate Wetland Restoration Opportunities in the Chesapeake Bay Watershed
Source: Environ Manage. 2022 Oct 8;70(6):950–64. doi: 10.1007/s00267-022-01725-5 (PMC9622542; doi:10.1007/s00267-022-01725-5)

**Electronic Supplemental Material:** Using structured decision making to evaluate wetland restoration opportunities in the Chesapeake Bay watershed

**Journal**: Environmental Management

**Authors**: David M. Martin, Amy D. Jacobs, Chase McLean, Michelle R. Canick, Kathleen Boomer

**Correspondence**: David M. Martin: [David.Martin@tnc.org](about:blank), (301) 897.8570 (Main), (301) 897.0858 (Fax)

**Restoration alternatives**

The following steps were performed in ArcGIS Pro:

1. Extracted land use classes from 1-meter resolution Chesapeake Conservancy High-Resolution Land Use (<https://www.chesapeakeconservancy.org/conservation-innovation-center/high-resolution-data/land-use-data-project/>):

| Land Use Code | Land Use Name |
| --- | --- |
| 5 | Tidal Wetlands |
| 6 | Floodplain Wetlands |
| 7 | Other Wetlands |
| 8 | Forest |
| 10 | Mixed Open |
| 12 | Fractional Turf (medium) |
| 13 | Fractional Turf (large) |
| 16 | Cropland |

1. Add additional areas of cropland missing from the Chesapeake Conservancy data using USDA NASS Cropland Data Layer. The added cropland areas were assigned a Land Use Code of 18.
2. Remove tidal wetlands using USFWS National Wetlands Inventory. Chesapeake Conservancy data maps large areas of other land cover classes as tidal wetlands, particularly on the Lower Eastern Shore of Maryland, so we did not want to rely on that data source to remove tidal wetlands.
3. Remove roads using MD SHA Road Centerlines converted to a 3-meter resolution raster. Roads are included in the Chesapeake Conservancy land use, but they are not always continuous.
4. Remove agricultural land use (Conservancy Land Use (16) Cropland or (18) Additional Cropland) that is located on prime farmland soils (USDA NRCS SSURGO Farmland Class = All Areas are Prime Farmland).
5. Create contiguous patches using the Region Group geoprocessing tool.
6. Convert regions to multipart polygons.
7. Remove polygons less than 300 acres in size.
8. Remove polygons with less than 150 acres of ecohydrologically active area.

**Objectives and criteria**

Source for Maryland county DEMs: <https://imap.maryland.gov/Pages/lidar-dem-download-files.aspx>. Source for Delaware county DEMs: <https://apps.nationalmap.gov/downloader/#/4/37.99999999999935/-94.9999999999984/usgs_topo/elevation-products-three-dep/one-meter-dem>. All DEMs were downloaded at 1-meter resolution and those that were above or below this resolution were resampled to 1-meter in ArcGIS Pro software.

For watersheds that spanned multiple HUC8 boundaries, the loading rate for each HUC was attributed to the area that fell within that HUC. The CAST system only has values for areas that flow into the Chesapeake Bay. Portions of delineated watersheds that fell outside of this area required some manual assignment of the nearest HUC within the Chesapeake Bay watershed for calculating loading rates. In these cases, we assigned these areas the loading rate for the adjacent HUC8 in which the portion of the watershed flowed into.

**Principal component analysis**

The following methods are based on previous research; see Martin et al (*in review*) for more details. The general steps to principal component analysis are as follows:

1. Criterion mean: $\bar{c_{j}}=\frac{1}{964}\sum_{i=1}^{964} z_{ij}$
2. Distance between observed and mean: $D_{ij}=z_{ij}-\bar{c_{j}}$
3. Criteria variance: $var\left( j \right)=\frac{1}{963}\sum_{i=1}^{964} D_{ij}^{2}$
4. Criteria standard deviation: $stdv\left( j \right)=\sqrt{var(j)}$
5. Criteria covariance: $cov\left( i,j \right)=\frac{1}{963}\sum_{i=1}^{964} \left( z_{ij}-\bar{c_{j}} \right)\left( z_{ij}-\bar{c_{j}} \right)$
6. Covariance matrix: $A=\begin{matrix} var(1) & cov(1,2) & \ldots& cov(1,7) \\ cov(2,1) & var(2) & \ldots& cov(2,7) \\ \vdots& \vdots& \ddots& \vdots\\ cov(7,1) & cov(7,2) & \ldots& var(7) \end{matrix}$

Eigenvalue analysis of the covariance matrix *A* is applied to minimize redundancy and maximize variation in the dataset (Shlens, 2014). Eigenvalue analysis calculates a vector $\vec{v}$ that satisfies $A\vec{v}=\lambda\vec{v}$, where $\vec{v}$ are the $n=1,\ldots,l$ eigenvectors of covariance matrix A and λ are the corresponding eigenvalues, sometimes referred to as characteristic roots. The functional form of a principal component $Z_{ni}$ initially takes each single-criterion value $z_{ij}$ and transforms it to between -1 and 1 using a linear scaling function $x_{ij}$:

|  | $x_{ij}=\frac{z_{ij}-\bar{z_{j}}}{s_{j}}$ | (1) |
| --- | --- | --- |

for all criteria *j*, alternatives *i*; where $\bar{z_{j}}$ is criterion mean and $s_{j}$ is its standard deviation. The first principal component $Z_{1i}$ aggregates the scaled criteria values into an overall value, sometimes referred to as a Z-score, per alternative *i*:

|  | $Z_{1i}=\sum_{n=1}^{l} v_{n1}x_{ij}$ | (2) |
| --- | --- | --- |

where coefficients $v_{n1}$ are elements of the eigenvector associated with the dominant eigenvalue $\lambda_{1}$ from covariance matrix *A*.

Shlens J (2014) A tutorial on principal component analysis. arXiv preprint. <https://arxiv.org/abs/1404.1100>

**Fig. S1** Frequency distributions per criterion under 2019 Scenario


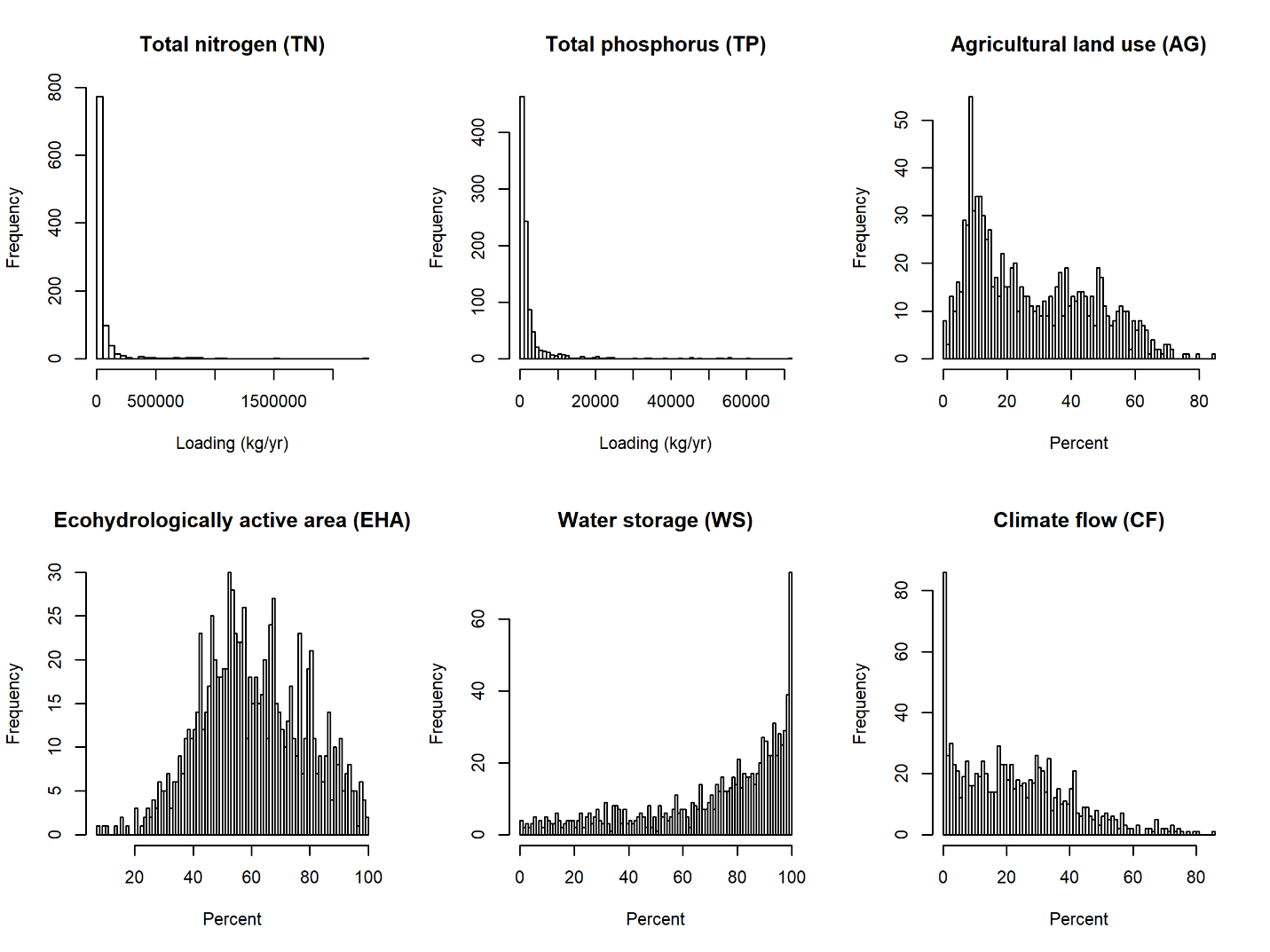


**Fig. S2** Frequency distributions per criterion under 2025 Scenario


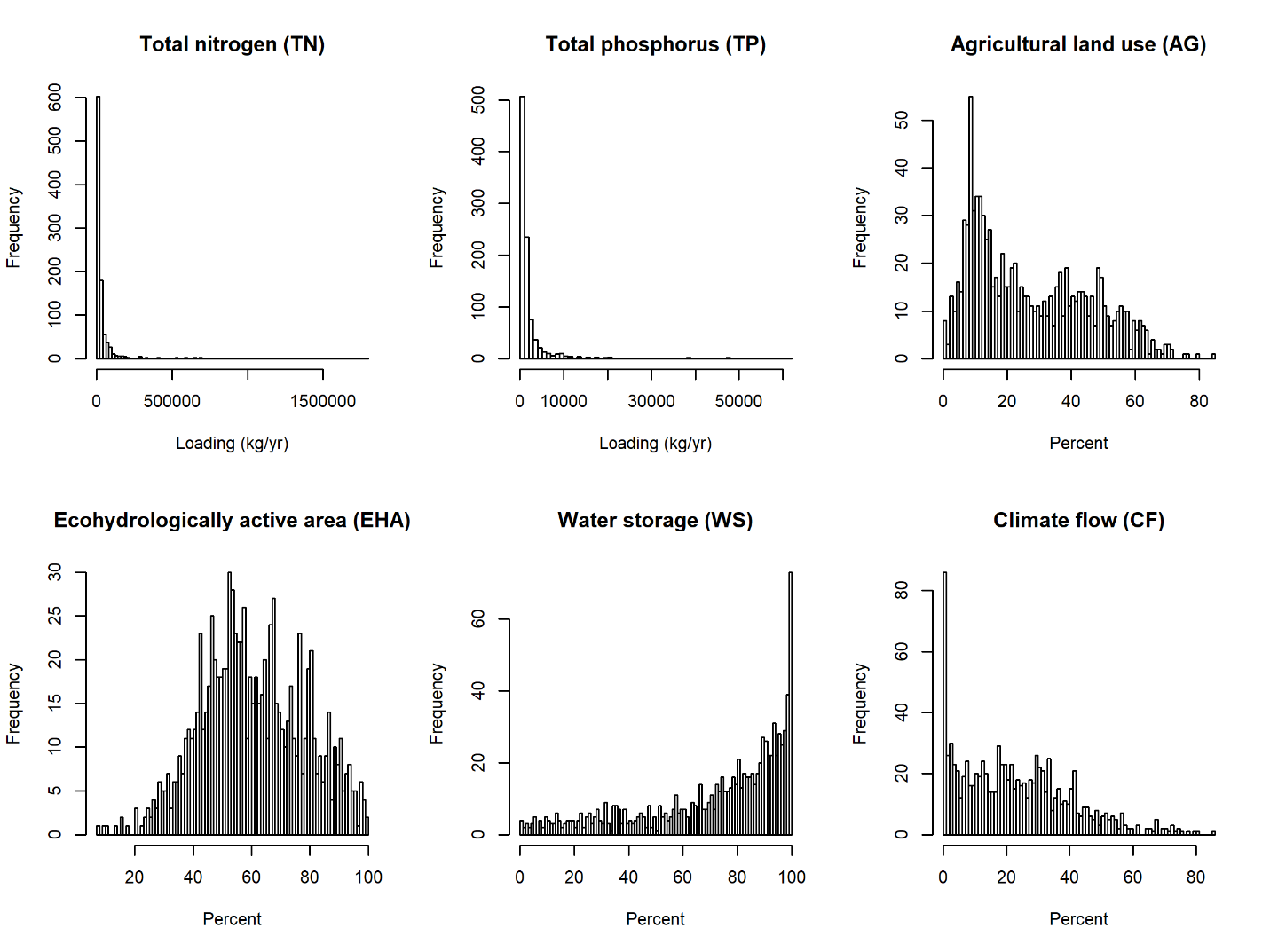


**Fig. S3** Nonlinear scaling function per TN, TP, CF criteria under 2019 Scenario


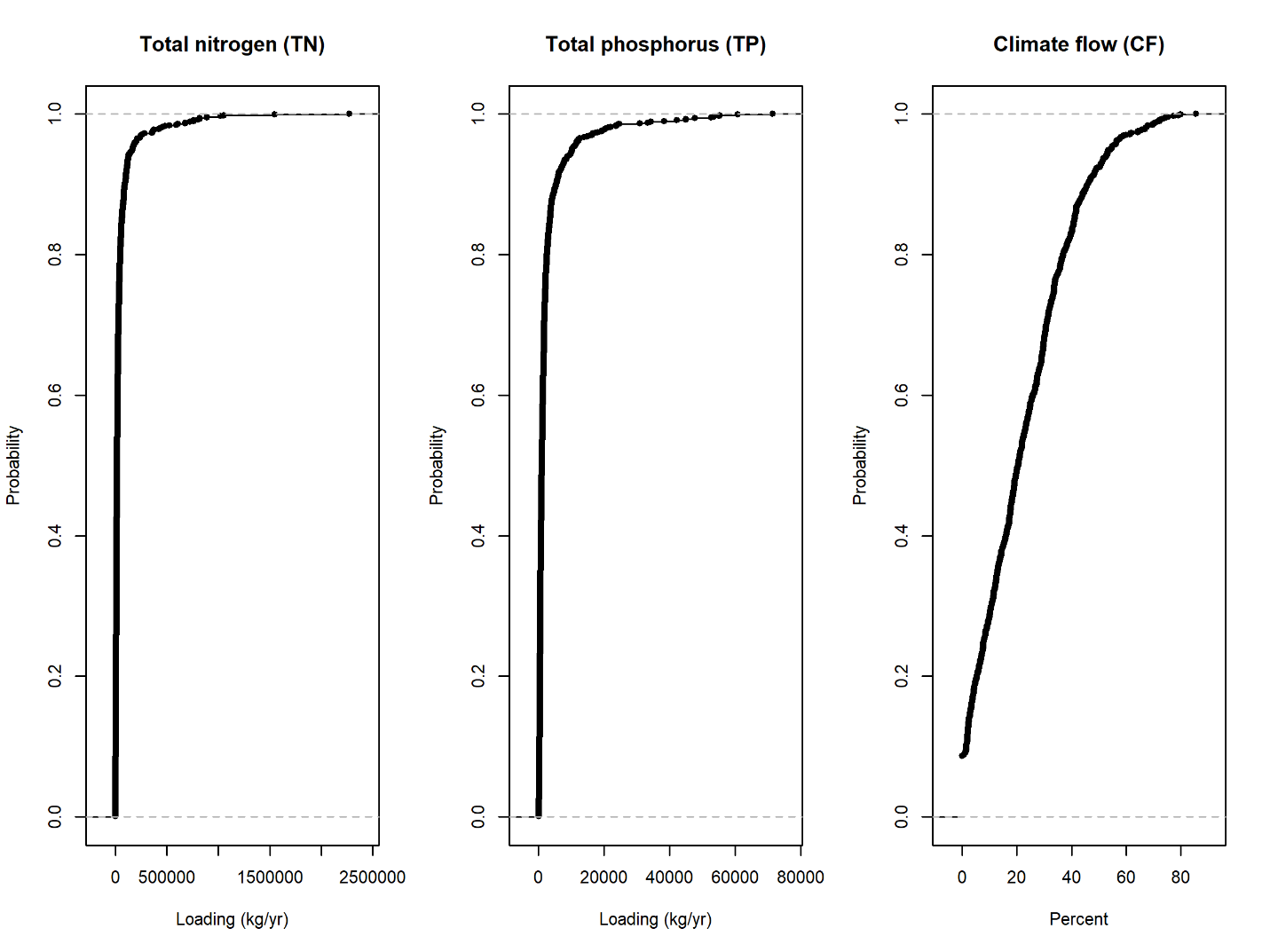


**Fig. S4** Nonlinear scaling function per TN, TP, CF criteria under 2025 Scenario


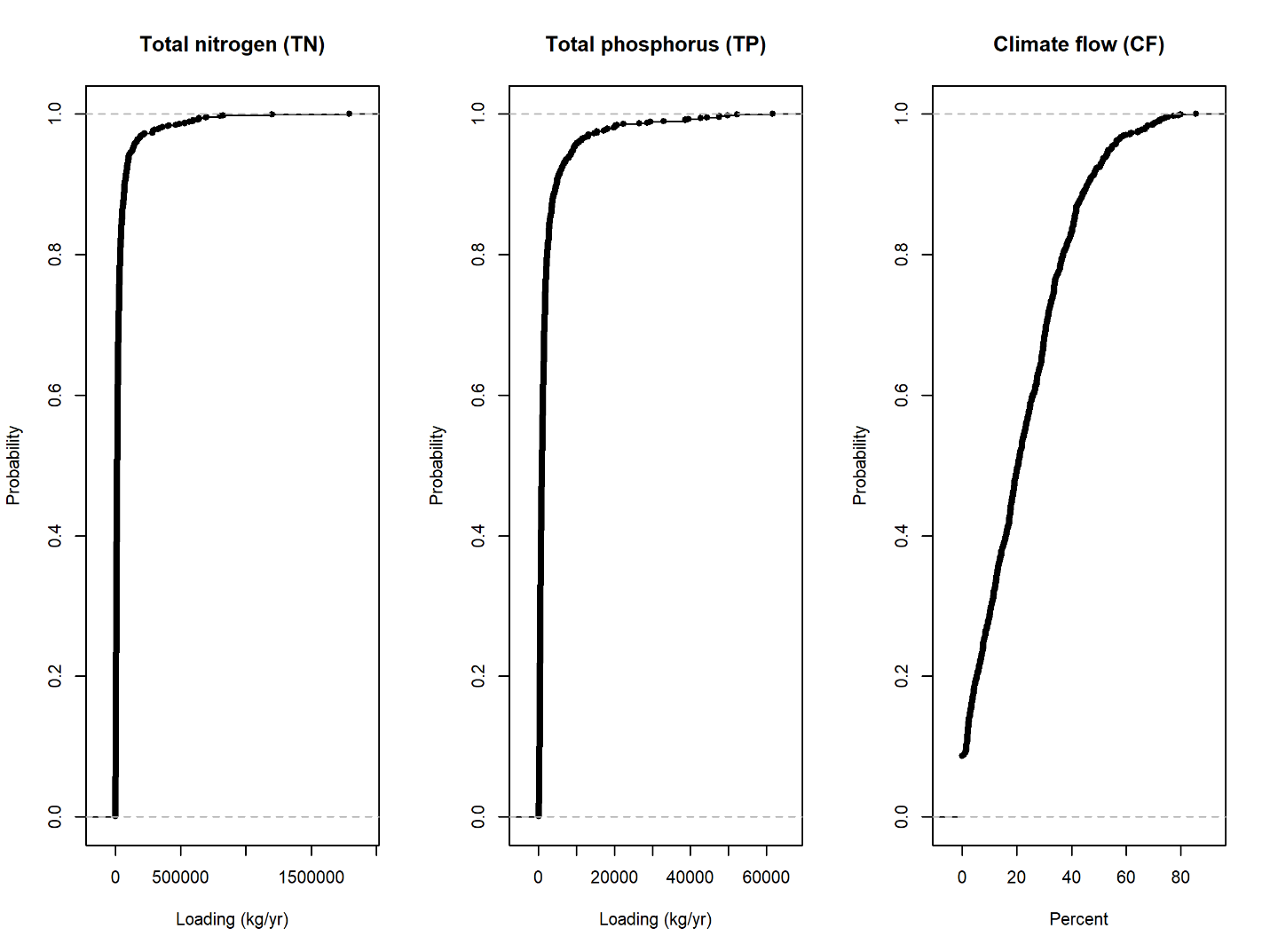


**Fig. S5** Priority alternatives across the study area under 2019 Scenario


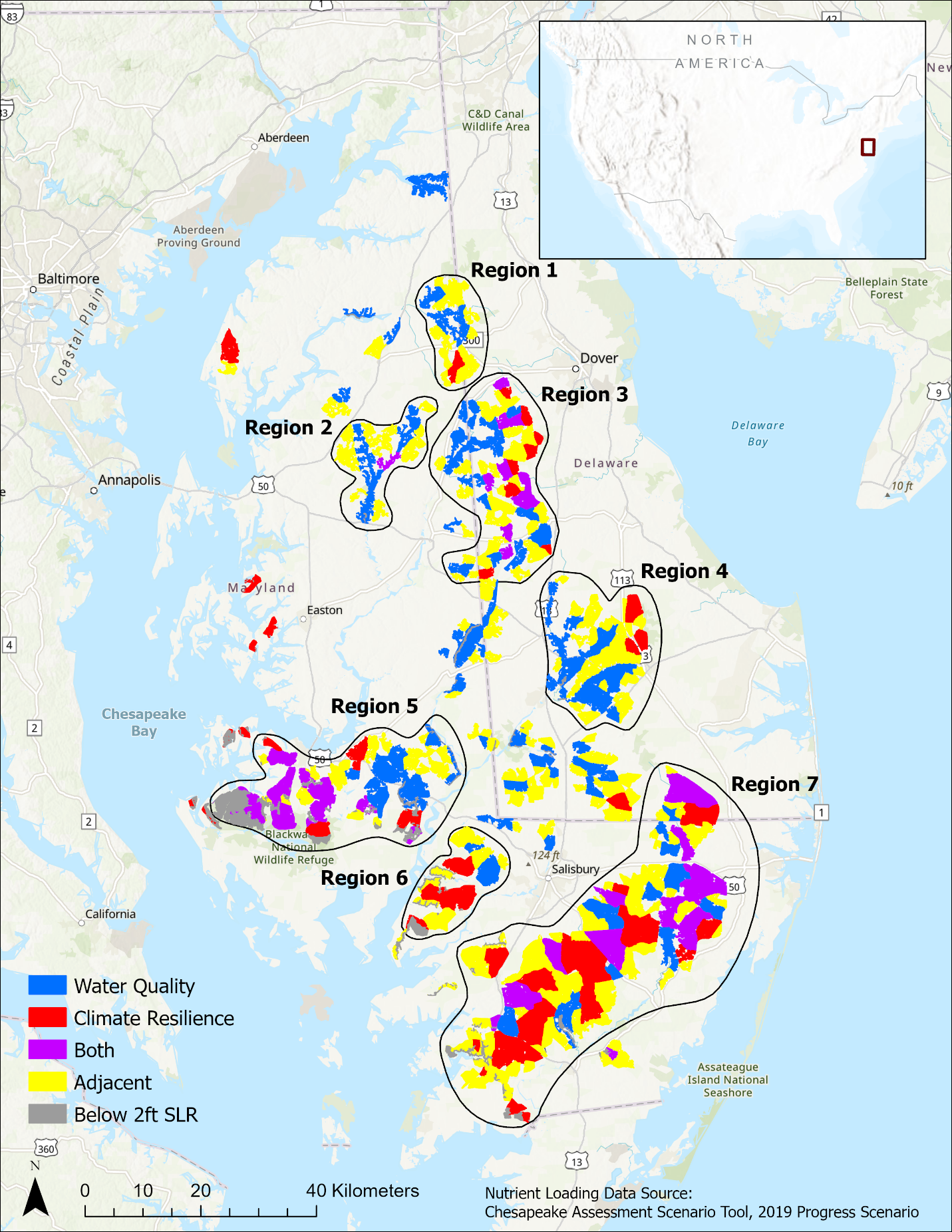

Supplement: Supplementary file 1 — Electronic Supplemental Material [file 267_2022_1725_MOESM1_ESM.docx]
