# Supplementary material for: Using Structured Decision Making to Evaluate Wetland Restoration Opportunities in the Chesapeake Bay Watershed
Source: Environ Manage. 2022 Oct 8;70(6):950–64. doi: 10.1007/s00267-022-01725-5 (PMC9622542; doi:10.1007/s00267-022-01725-5)
Supplement: Supplementary file 2 — Electronic Supplemental Material [file 267_2022_1725_MOESM2_ESM.pdf]

Electornic Supplemental Material: Using structured decision making to evaluate wetland restoration opportunities in the Chesapeake Bay watershed
Journal: Environmental Management
Authors: David M. Martin, Amy D. Jacobs, Chase McLean, Michelle R. Canick, Kathleen Boomer
Correspondence: David M. Martin: David.Martin@tnc.org, (301) 897.8570 (Main), (301) 897.0858 (Fax)

Table S1 Criteria measures per alternative, 2019 Scenario

Alternative	TN	TP	AG	EHA	FP	WS	CF
1	2,435.62	622.11	1.84	92.98	1.00	97.89	46.20
2	1,885.71	243.67	7.43	99.06	1.00	100.00	72.63
3	3,851.36	626.90	13.06	97.79	1.00	99.18	32.43
4	4,402.26	560.00	17.79	90.16	1.00	97.79	24.22
5	2,049.77	558.01	2.88	95.90	1.00	99.50	43.42
6	1,321.63	306.19	5.07	93.90	1.00	99.33	45.36
7	730.08	124.90	0.27	99.47	1.00	100.00	67.98
8	2,645.84	403.10	16.91	85.58	1.00	96.29	61.25
9	5,632.53	713.82	15.93	97.22	1.00	98.89	53.25
10	5,630.98	761.65	12.20	95.21	1.00	99.30	80.01
11	1,447.58	332.40	4.66	98.75	1.00	93.55	50.68
12	1,763.61	570.26	1.76	93.16	1.00	99.87	56.63
13	4,222.90	431.20	43.88	88.20	1.00	99.50	5.65
14	1,280.49	298.28	10.55	93.11	1.00	99.17	34.57
15	6,681.11	744.78	16.75	82.68	1.00	88.30	22.22
16	945.60	142.56	2.64	88.19	1.00	99.88	36.45
17	1,343.02	345.15	8.43	86.21	1.00	99.26	51.49
18	4,080.56	680.61	0.63	95.10	1.00	98.62	77.43
19	5,146.33	707.75	6.56	90.58	1.00	96.44	71.42
20	7,695.81	1,066.17	5.82	93.16	1.00	99.82	52.61
21	2,101.30	145.67	55.71	59.77	1.00	86.57	7.62
22	2,939.11	199.55	56.40	63.95	1.00	88.26	8.78
23	13,485.74	1,767.54	10.47	92.15	1.00	98.64	53.25
24	9,504.65	985.04	16.85	86.49	1.00	96.46	41.47
25	1,897.19	197.60	22.37	45.83	1.00	63.33	27.40
26	16,552.32	1,278.82	44.17	84.33	1.00	95.34	24.35
27	9,074.68	1,016.48	35.93	64.45	1.00	98.79	7.46
28	1,691.84	177.39	21.35	97.42	1.00	100.00	33.73
29	3,629.56	738.99	3.93	94.35	1.00	99.82	70.43
30	2,210.90	714.46	0.64	81.33	1.00	94.36	53.34
31	34,422.26	5,300.57	17.23	93.20	1.00	99.90	67.08
32	1,410.34	145.89	22.52	92.36	1.00	99.09	39.77
33	2,126.51	253.43	14.76	88.39	1.00	95.26	35.06
34	2,804.15	257.59	26.47	76.27	1.00	91.06	37.07
35	1,818.09	412.75	9.55	93.27	1.00	97.79	26.86
36	6,409.04	624.88	18.31	83.37	1.00	95.59	54.39
37	3,774.13	58.13	79.55	53.20	1.00	99.99	2.53
38	1,658.00	199.97	3.35	87.16	1.00	98.21	85.60
39	10,892.69	890.23	39.37	81.01	1.00	89.81	47.88
40	1,303.96	241.53	18.06	91.73	1.00	100.00	18.58
41	13,315.75	970.40	50.25	89.84	1.00	98.57	18.94
42	13,181.04	1,591.02	25.76	89.98	1.00	99.72	34.46
43	6,781.77	537.54	41.57	82.81	1.00	99.95	3.47
44	53,163.59	4,388.25	38.15	82.06	1.00	99.07	30.56
45	27,402.14	1,830.28	46.68	53.28	1.00	72.88	19.57
46	4,784.40	392.16	34.22	80.28	0.00	98.34	9.77
47	15,035.87	1,122.09	46.61	73.15	1.00	93.74	29.63
48	2,543.27	225.79	51.22	78.61	1.00	99.64	10.89
49	13,746.84	1,259.14	30.01	73.26	1.00	89.59	28.96
50	4,061.94	375.80	29.43	61.12	0.00	98.98	16.25
51	8,997.48	875.12	32.69	77.88	0.00	97.53	18.83
52	15,828.53	2,037.04	22.34	88.52	1.00	98.51	32.29
53	2,445.02	257.33	25.22	69.57	0.00	97.43	30.03
54	1,842.03	165.67	29.04	85.23	0.00	94.58	55.55
55	65,568.58	4,490.25	44.60	67.78	1.00	83.96	32.52
56	3,956.48	731.46	17.88	70.77	1.00	98.56	40.44
57	4,733.52	787.31	22.01	88.37	1.00	99.35	36.75
58	2,406.33	280.37	37.66	77.14	1.00	84.80	44.34
59	3,175.46	213.88	17.02	73.35	0.00	76.77	18.24
60	24,331.29	1,971.60	37.67	70.89	1.00	90.78	20.64
61	2,865.11	519.95	18.80	85.78	1.00	98.88	65.14
62	21,092.12	1,636.82	41.23	50.54	0.00	71.00	5.49
63	10,773.79	756.85	35.32	52.56	0.00	73.69	30.67
64	2,117.84	197.85	44.25	90.99	1.00	99.37	12.45
65	2,393.03	189.58	42.78	67.95	0.00	68.24	15.17
66	18,797.96	571.27	24.53	51.63	1.00	75.55	13.56
67	1,230.27	86.01	2.15	54.96	0.00	93.62	19.61
68	12,195.73	537.77	51.99	70.53	0.00	77.53	16.37
69	30,637.70	2,071.28	52.59	57.92	1.00	63.90	20.09
70	8,466.25	1,012.81	22.96	74.21	1.00	90.72	37.96
71	5,379.27	429.48	38.78	61.74	0.00	97.46	27.57
72	2,347.41	66.90	27.23	55.84	0.00	36.66	4.68
73	8,578.76	187.91	43.54	40.20	0.00	64.00	5.65
74	9,747.93	611.99	61.28	68.69	0.00	91.64	7.09
75	58,764.47	1,209.50	48.82	20.23	1.00	40.70	6.38
76	14,775.69	352.53	38.10	56.73	1.00	38.59	45.97
77	23,548.21	388.11	70.92	40.64	1.00	5.66	0.00
78	16,026.91	257.80	76.56	46.39	0.00	31.98	8.93
79	2,838.87	223.88	57.82	47.03	1.00	92.14	1.94
80	1,770.51	172.37	48.29	62.84	1.00	89.33	8.00
81	16,479.73	1,055.63	63.96	25.46	1.00	59.66	0.00
82	9,828.87	194.98	71.36	48.10	0.00	14.93	0.00
83	3,762.45	300.44	55.36	40.66	1.00	73.79	1.94
84	53,209.97	966.51	60.08	48.61	1.00	14.61	0.00
85	1,339.53	386.50	4.82	98.09	0.00	100.00	21.15

Correlation	*TN*	*TP*	*AG*	*EHA*	*FP*	*WS*	*CF*
TN	1						
TP	0.8171887	1					
AG	0.0135898	0.0447293	1				
EHA	-0.157392	-0.190954	-0.344739	1			
FP	0.1435206	0.1415856	-0.271029	0.1430425	1		
WS	-0.182283	-0.073581	0.0084118	0.42511	-0.04344	1	
CF	0.2258599	0.2951816	-0.317258	0.0965154	0.1299926	0.1687358	1

86	6,684.99	635.71	48.04	63.78	1.00	92.12	18.77
87	18,560.26	309.53	69.61	44.18	0.00	18.84	16.32
88	764.55	247.61	0.25	97.01	0.00	100.00	33.93
89	7,759.62	115.48	84.37	71.82	0.00	21.35	0.00
90	50,505.72	913.25	61.63	33.09	1.00	35.47	20.04
91	17,923.01	415.69	48.47	43.86	0.00	11.04	0.00
92	38,342.14	719.62	56.82	46.56	1.00	36.23	14.24
93	18,706.61	404.48	44.51	42.05	0.00	58.64	23.29
94	24,623.75	641.14	32.41	35.59	1.00	36.11	53.45
95	1,070.99	74.86	2.28	51.80	0.00	59.60	39.50
96	2,001.73	236.04	35.27	78.61	1.00	99.92	10.87
97	4,297.37	160.53	17.24	76.68	0.00	77.98	40.32
98	5,274.30	112.63	46.06	80.40	0.00	75.10	11.13
99	3,320.34	297.58	50.09	53.92	0.00	88.83	0.00
100	19,123.10	388.92	49.33	33.87	1.00	30.99	26.91
101	6,762.85	488.82	50.61	49.24	0.00	76.57	9.16
102	9,629.08	179.75	57.26	57.57	0.00	63.45	2.73
103	11,955.12	274.90	40.18	31.01	0.00	54.94	20.95
104	9,726.81	154.50	75.53	73.71	0.00	79.91	1.74
105	3,711.80	109.56	22.95	60.57	0.00	5.09	21.89
106	2,743.71	278.63	45.12	48.73	1.00	99.56	9.68
107	10,773.22	870.57	49.86	46.89	1.00	55.23	1.43
108	3,917.97	355.68	49.94	31.78	1.00	99.66	22.11
109	10,620.38	199.95	56.30	66.67	1.00	75.25	10.29
110	7,216.62	177.06	35.18	53.40	0.00	63.39	41.74
111	1,643.23	256.77	25.47	66.98	1.00	98.42	39.80
112	52,504.62	1,076.62	48.13	39.74	1.00	3.46	34.32
113	12,763.37	231.25	60.36	65.34	0.00	64.66	21.27
114	4,583.84	607.30	31.38	51.20	0.00	78.32	29.61
115	1,474.26	318.71	14.23	80.52	1.00	99.24	45.66
116	1,413.26	216.54	27.04	64.17	1.00	96.52	5.10
117	1,880.67	217.69	31.48	63.66	0.00	93.91	0.00
118	5,397.90	336.00	52.01	66.17	0.00	78.06	22.76
119	8,998.98	783.64	51.44	17.62	1.00	49.07	0.88
120	3,524.77	535.36	25.69	43.42	0.00	68.62	29.58
121	1,313.22	308.57	11.39	76.65	1.00	98.83	41.89
122	1,214.33	228.08	13.81	83.54	0.00	99.37	18.09
123	46,393.06	878.15	61.91	55.55	1.00	75.90	13.19
124	1,024.15	240.27	7.96	61.87	0.00	86.83	39.94
125	13,779.47	256.70	63.46	56.56	1.00	73.81	7.26
126	11,380.13	218.88	54.42	74.26	1.00	93.29	28.97
127	2,078.08	268.93	27.27	66.45	1.00	99.31	7.92
128	49,821.52	1,009.11	49.82	57.51	1.00	62.00	2.56
129	7,992.96	259.09	58.88	66.93	1.00	79.61	21.88
130	3,116.88	496.72	14.20	80.39	0.00	95.91	20.10
131	47,493.84	988.98	54.72	52.94	1.00	48.61	37.11
132	13,051.21	279.58	49.71	52.17	1.00	85.04	3.21
133	1,098.38	157.84	19.22	76.87	1.00	96.24	42.56
134	818.59	210.41	0.62	90.80	0.00	100.00	19.95
135	701,585.01	15,125.51	46.19	52.99	1.00	37.09	57.66
136	12,353.72	806.53	50.32	41.14	1.00	91.58	2.50
137	10,357.66	201.41	53.43	46.92	0.00	82.24	13.19
138	9,354.03	185.03	52.12	45.14	1.00	77.78	10.39
139	2,393.65	272.20	28.80	55.35	1.00	88.02	44.22
140	8,692.55	716.47	50.16	50.41	1.00	57.84	7.82
141	23,074.85	519.75	54.73	41.24	1.00	78.00	15.94
142	15,931.96	1,157.84	56.05	31.41	1.00	27.31	56.27
143	1,040.08	218.13	6.60	79.67	0.00	96.01	43.15
144	7,031.77	653.11	36.79	42.92	1.00	85.32	32.30
145	11,447.90	628.27	66.65	35.21	1.00	56.41	0.00
146	10,634.42	782.70	46.13	51.08	1.00	61.95	18.98
147	3,064.47	391.07	35.56	41.13	1.00	67.07	51.36
148	3,774.34	193.28	67.23	52.13	1.00	92.47	7.68
149	6,213.14	118.74	55.37	52.48	0.00	84.22	0.00
150	1,777.59	276.00	16.03	71.19	0.00	96.42	30.74
151	11,706.90	738.15	62.11	50.19	1.00	67.30	15.24
152	4,710.50	336.31	60.14	46.58	0.00	75.33	19.13
153	36,658.24	752.16	50.56	42.99	1.00	42.88	33.63
154	3,537.28	365.74	32.90	40.87	1.00	84.82	3.15
155	11,414.19	220.67	56.60	48.05	0.00	85.12	7.62
156	4,689.91	559.42	35.91	41.74	0.00	35.30	26.30
157	4,917.26	373.19	57.98	43.59	1.00	49.30	12.03
158	1,311.98	191.34	18.61	56.40	1.00	75.11	24.78
159	8,649.90	517.86	65.20	39.76	1.00	57.92	14.58
160	4,180.36	93.03	46.95	47.15	0.00	80.27	12.22
161	7,628.64	467.14	62.33	47.66	0.00	87.14	11.56
162	9,241.04	195.09	47.08	64.08	0.00	90.68	3.70
163	3,951.22	323.31	54.06	43.77	0.00	73.86	29.85
164	5,015.25	322.26	52.84	26.48	1.00	51.01	8.58
165	5,160.81	122.52	38.64	61.61	1.00	87.00	13.47
166	16,604.46	491.66	29.23	64.71	1.00	87.73	38.52
167	15,773.43	1,091.29	49.01	59.35	1.00	72.47	48.15
168	4,924.47	333.07	49.71	75.95	0.00	88.70	21.66
169	38,251.71	3,334.18	48.25	42.47	1.00	38.96	33.78
170	13,973.77	397.10	32.40	47.99	1.00	93.50	33.45
171	7,181.16	126.55	63.07	74.42	1.00	99.21	5.64
172	2,311.68	357.95	26.97	79.08	0.00	78.22	33.81
173	3,635.60	288.35	54.38	51.80	0.00	79.00	28.00
174	4,744.44	624.87	17.95	39.28	1.00	69.42	52.67
175	191,767.07	4,010.77	48.50	40.24	1.00	35.57	33.59
176	26,231.71	540.99	48.95	61.16	1.00	93.87	31.30

177	13,983.08	842.10	67.98	32.94	1.00	77.81	4.04
178	5,663.63	456.42	51.08	42.33	0.00	59.14	10.66
179	10,136.81	253.03	33.65	48.60	1.00	58.63	14.31
180	29,487.84	2,611.28	47.32	44.66	0.00	44.02	36.69
181	6,614.21	136.57	47.91	71.12	1.00	88.11	9.57
182	10,824.07	883.58	49.14	46.62	1.00	66.45	11.54
183	3,113.29	299.31	47.26	49.33	0.00	63.57	20.25
184	1,192.91	201.91	7.76	55.09	0.00	85.55	40.25
185	31,337.20	606.34	55.60	51.76	1.00	93.36	32.20
186	9,173.90	192.93	48.23	66.77	1.00	95.75	19.19
187	17,832.42	371.94	48.02	65.55	1.00	97.32	15.75
188	12,421.45	771.25	57.87	15.69	1.00	38.33	40.78
189	13,897.51	278.71	50.99	73.13	1.00	96.82	23.72
190	2,917.42	80.22	28.32	76.75	1.00	95.88	30.49
191	19,133.22	383.58	53.36	60.16	1.00	89.17	19.78
192	7,487.16	303.25	49.95	65.13	1.00	96.11	12.95
193	169,983.09	3,607.32	47.45	49.39	1.00	86.52	24.61
194	4,328.04	117.50	28.06	79.15	1.00	91.71	42.93
195	8,709.89	488.17	61.14	36.01	0.00	92.39	2.90
196	41,089.64	910.06	42.28	37.79	1.00	97.70	3.53
197	4,448.25	98.31	43.23	77.35	1.00	63.64	16.57
198	5,492.97	533.18	44.48	40.36	0.00	82.46	17.08
199	7,411.29	158.68	44.61	59.88	1.00	83.24	12.39
200	10,490.47	659.77	62.63	52.97	1.00	80.52	30.42
201	38,533.86	2,528.60	62.37	24.55	1.00	86.44	9.10
202	58,850.49	3,544.93	65.51	35.24	1.00	51.11	33.16
203	10,892.13	203.45	65.03	53.75	1.00	98.96	17.58
204	2,695.62	411.96	24.58	44.45	0.00	65.58	7.85
205	7,450.33	833.92	34.65	39.13	0.00	81.11	24.73
206	14,424.93	328.25	41.39	71.37	1.00	96.38	42.30
207	49,385.53	3,916.90	52.07	25.80	1.00	34.54	51.75
208	6,961.64	614.47	49.65	47.91	0.00	82.51	14.00
209	32,888.43	2,152.33	63.72	42.21	1.00	66.70	9.45
210	28,930.11	1,615.18	62.00	20.03	0.00	25.05	24.78
211	8,262.80	754.17	36.18	45.16	0.00	74.21	31.83
212	25,913.90	602.45	38.59	75.82	1.00	99.26	26.55
213	20,613.55	1,446.12	49.40	43.27	0.00	60.67	18.48
214	7,554.13	149.97	51.68	78.28	1.00	99.13	17.42
215	10,816.28	228.31	50.68	66.96	1.00	94.98	20.76
216	14,151.77	752.44	62.32	26.38	0.00	59.23	2.32
217	23,163.61	1,714.92	54.93	44.05	1.00	25.98	11.31
218	11,280.07	748.07	57.22	79.19	1.00	95.62	1.50
219	10,336.57	564.69	69.33	64.63	1.00	70.59	22.71
220	25,714.11	1,706.41	59.88	42.32	1.00	55.06	20.82
221	9,789.37	927.51	41.70	44.38	0.00	73.96	46.57
222	21,773.67	1,371.09	62.46	50.01	1.00	57.75	19.16
223	7,460.16	166.64	41.75	80.88	1.00	98.82	25.66
224	55,300.88	1,327.21	40.88	67.53	1.00	99.29	22.86
225	18,229.96	474.51	39.84	73.85	1.00	95.31	25.07
226	6,427.76	566.09	48.93	53.51	1.00	72.82	7.64
227	10,088.96	995.17	45.56	77.44	1.00	94.77	21.03
228	5,143.99	95.74	57.67	79.47	1.00	89.99	4.46
229	275,271.85	21,665.12	53.18	41.30	1.00	52.36	51.74
230	6,102.44	647.81	42.09	84.55	1.00	97.12	24.24
231	6,871.95	406.05	68.63	46.33	0.00	79.88	28.99
232	5,536.37	338.96	64.28	60.98	0.00	64.59	11.95
233	22,108.81	1,250.07	60.88	39.19	0.00	51.29	23.11
234	6,922.10	718.53	35.41	91.20	1.00	98.81	21.07
235	19,516.99	447.32	42.78	81.08	1.00	96.22	47.37
236	22,984.16	567.83	43.04	67.69	1.00	99.18	29.84
237	3,633.55	363.02	31.23	73.60	1.00	97.84	19.95
238	32,017.15	2,545.00	53.56	35.55	1.00	64.91	12.27
239	63,052.82	4,458.82	57.77	45.98	1.00	53.82	21.44
240	8,714.97	260.71	38.86	83.15	1.00	98.80	35.87
241	6,055.60	563.59	46.57	53.90	0.00	75.45	17.91
242	7,916.25	604.60	54.77	52.90	1.00	80.79	2.96
243	27,032.15	2,192.42	50.23	52.31	1.00	87.04	14.44
244	82,012.94	5,800.49	57.01	33.21	1.00	69.57	24.54
245	4,443.90	375.17	39.54	35.29	1.00	80.06	4.47
246	17,487.78	1,299.19	55.49	43.91	0.00	88.92	20.08
247	20,444.59	1,713.60	48.55	47.16	1.00	90.47	35.04
248	2,673.34	228.39	48.09	73.15	0.00	81.14	2.65
249	161,824.62	14,005.99	49.67	41.71	1.00	72.25	54.14
250	6,238.17	191.92	57.76	76.27	1.00	92.80	4.21
251	4,940.90	309.68	63.92	48.84	0.00	83.64	12.81
252	7,897.91	823.43	42.03	44.59	1.00	64.97	8.60
253	11,473.10	756.04	38.03	82.01	1.00	90.48	36.36
254	59,178.53	4,201.14	56.52	39.97	1.00	67.19	19.28
255	7,826.17	590.84	56.97	68.58	1.00	94.39	13.87
256	6,948.19	825.56	36.96	45.58	0.00	75.12	28.66
257	6,920.91	511.25	59.12	59.52	0.00	82.85	20.50
258	4,526.52	385.22	51.86	79.25	1.00	97.31	13.79
259	4,099.77	363.76	48.66	86.12	1.00	93.04	4.54
260	26,402.74	1,735.70	49.18	23.18	1.00	41.30	17.20
261	2,461.70	398.68	22.76	63.50	0.00	76.80	12.70
262	7,391.50	513.01	59.34	46.79	0.00	91.43	17.44
263	220,695.12	24,181.12	37.77	34.83	1.00	39.42	41.33
264	10,829.76	694.37	65.23	41.20	0.00	79.52	15.61
265	8,083.19	1,160.44	29.62	78.15	1.00	97.93	45.74
266	3,880.36	575.65	27.16	86.99	1.00	95.20	48.41
267	3,778.02	395.12	39.20	45.60	0.00	76.07	17.11

268	3,336.94	337.30	41.63	73.46	1.00	94.43	0.00
269	10,770.73	626.66	63.52	41.50	0.00	93.27	6.54
270	9,106.19	572.42	54.69	31.43	0.00	80.67	19.18
271	5,508.49	973.41	20.92	52.91	0.00	80.17	42.61
272	42,434.51	3,042.68	55.14	27.70	0.00	86.43	18.50
273	25,487.88	1,902.14	54.47	27.95	0.00	81.45	27.37
274	6,360.47	1,044.29	22.40	83.22	1.00	92.41	34.56
275	9,079.44	597.58	61.95	43.19	0.00	89.07	9.48
276	14,344.18	1,060.44	57.05	51.63	0.00	87.50	7.73
277	25,794.01	2,994.72	35.99	53.69	1.00	85.07	33.25
278	120,792.76	10,918.59	48.30	43.18	1.00	78.09	25.25
279	165,400.69	19,134.88	35.32	44.15	1.00	31.66	42.23
280	20,844.05	1,389.91	51.63	28.16	0.00	73.09	11.27
281	7,548.59	473.47	56.15	38.89	0.00	89.57	0.00
282	9,444.84	1,660.75	18.00	76.61	1.00	93.27	31.00
283	3,880.50	621.12	24.08	59.55	0.00	84.70	10.44
284	12,835.50	2,155.57	19.96	72.56	1.00	82.57	21.88
285	80,125.26	10,018.79	31.95	41.69	1.00	43.25	36.42
286	3,255.21	839.11	6.58	86.31	1.00	85.52	48.91
287	26,232.23	2,633.24	41.50	68.46	1.00	76.98	30.37
288	13,457.87	1,293.06	43.50	56.35	1.00	89.80	0.00
289	5,317.03	580.68	38.21	47.02	0.00	68.47	35.24
290	18,520.95	1,032.60	61.35	29.73	0.00	65.46	10.02
291	54,885.60	5,579.70	43.06	38.58	1.00	86.39	2.32
292	12,307.37	1,839.35	27.78	50.19	1.00	75.70	27.39
293	4,866.49	418.49	45.15	52.31	0.00	92.19	21.92
294	25,298.12	1,501.32	58.08	30.74	0.00	66.27	7.48
295	44,762.40	6,315.99	26.25	55.62	1.00	83.94	32.37
296	25,647.09	3,499.98	27.86	68.36	1.00	95.28	0.00
297	5,525.86	1,330.94	8.58	89.14	1.00	91.81	33.99
298	13,284.01	933.22	59.17	48.04	0.00	96.17	13.92
299	1,306.25	395.82	2.78	90.01	1.00	89.33	48.51
300	5,706.23	905.65	24.41	67.78	1.00	72.64	30.01
301	39,114.58	3,849.10	44.60	51.84	1.00	85.42	25.46
302	10,732.85	739.84	52.00	27.57	0.00	74.69	12.98
303	17,920.28	2,759.61	23.45	68.73	1.00	91.57	12.21
304	55,360.35	5,530.97	41.08	54.95	1.00	78.19	31.24
305	6,990.29	1,256.90	19.25	62.40	1.00	96.96	16.75
306	47,759.01	4,729.26	41.35	55.03	1.00	79.00	21.90
307	1,960.42	279.84	25.12	69.70	0.00	76.63	25.93
308	18,291.54	1,680.90	46.76	30.84	0.00	85.04	14.76
309	48,954.48	2,767.57	60.94	29.91	1.00	58.65	0.00
310	6,220.56	747.51	35.78	40.81	0.00	98.46	19.82
311	28,151.67	2,874.86	42.99	50.62	1.00	89.19	6.92
312	33,759.94	3,580.92	37.26	72.99	1.00	97.26	30.88
313	6,481.84	582.33	47.26	64.62	1.00	89.56	10.32
314	2,118.23	324.21	20.45	72.84	0.00	95.46	28.64
315	89,998.49	9,661.45	35.42	44.39	1.00	56.88	38.40
316	7,067.37	849.18	33.38	83.51	1.00	99.13	31.53
317	4,467.82	260.35	59.31	36.92	0.00	82.67	0.00
318	3,432.81	547.44	20.88	49.44	0.00	70.12	31.46
319	6,824.60	542.44	46.89	65.72	1.00	86.85	2.97
320	73,642.98	7,434.71	38.07	51.75	1.00	72.13	18.18
321	2,301.16	353.63	21.77	80.21	1.00	91.37	29.71
322	6,442.71	637.30	44.84	63.85	0.00	96.12	3.19
323	13,581.82	1,678.96	30.67	75.00	1.00	99.80	26.64
324	5,518.14	764.40	26.18	82.56	1.00	94.41	40.32
325	10,572.58	865.96	47.30	57.68	0.00	96.41	26.50
326	9,445.01	878.11	45.97	68.91	0.00	94.23	32.99
327	8,410.01	905.44	38.45	79.41	1.00	91.79	7.71
328	5,907.96	679.26	36.81	59.02	0.00	77.33	29.80
329	15,782.60	1,942.61	34.86	41.31	1.00	87.64	14.61
330	7,090.11	811.68	35.72	46.68	0.00	74.28	7.84
331	5,374.18	703.09	30.01	48.67	0.00	84.67	13.16
332	6,804.97	823.51	32.68	87.45	1.00	97.98	5.00
333	6,246.11	597.38	40.25	72.10	1.00	99.48	21.88
334	3,632.90	276.53	53.06	80.29	1.00	99.19	1.69
335	2,003.82	322.39	21.57	67.26	1.00	80.80	41.88
336	3,516.72	367.99	26.11	68.21	0.00	88.41	10.09
337	3,567.17	456.31	32.65	84.17	1.00	98.52	36.96
338	6,591.33	504.87	45.31	20.15	0.00	65.22	17.18
339	4,029.47	344.16	40.73	52.62	0.00	78.34	23.32
340	5,717.54	827.76	23.47	68.61	1.00	93.63	35.75
341	9,029.00	1,058.76	24.10	58.61	0.00	77.95	46.57
342	14,848.00	1,797.28	30.86	83.77	1.00	97.87	31.05
343	13,229.73	1,351.12	42.18	51.34	0.00	92.60	18.22
344	29,115.09	1,689.26	59.59	36.16	0.00	59.40	19.40
345	6,137.25	713.68	36.69	53.49	1.00	85.78	23.98
346	4,800.75	229.61	70.38	49.08	0.00	87.06	0.00
347	8,676.77	604.40	53.04	67.93	1.00	89.37	0.00
348	4,787.29	579.37	24.82	63.63	0.00	78.38	44.03
349	6,412.19	884.44	25.26	63.72	1.00	89.78	15.07
350	4,614.42	528.54	32.23	80.69	1.00	86.65	23.78
351	7,779.30	766.47	34.01	31.57	0.00	77.33	33.43
352	9,980.61	1,147.33	28.30	61.96	1.00	84.35	41.88
353	4,409.55	344.80	45.05	49.88	0.00	90.18	32.23
354	9,459.40	995.86	31.25	47.80	0.00	79.76	30.67
355	3,682.52	361.80	29.20	55.69	1.00	74.36	11.86
356	3,860.55	407.05	36.67	85.87	1.00	92.74	28.91
357	4,565.22	541.61	26.60	56.55	1.00	76.71	40.77
358	6,214.77	608.04	33.20	53.18	0.00	63.14	41.52

359	21,979.42	1,892.92	40.71	42.81	0.00	75.38	20.72
360	18,342.76	1,550.53	38.53	48.27	1.00	60.48	17.59
361	9,387.25	597.01	55.93	31.68	0.00	82.69	3.35
362	6,166.26	522.72	41.15	47.59	0.00	72.49	32.49
363	77,609.05	4,520.38	59.49	28.02	0.00	65.66	18.73
364	3,873.57	275.59	46.91	78.61	1.00	74.96	19.48
365	3,396.17	241.01	48.88	77.14	1.00	95.40	4.90
366	54,707.22	4,696.59	39.73	52.86	1.00	73.06	33.77
367	66,814.49	5,541.19	41.60	37.23	1.00	70.04	37.71
368	5,484.79	413.98	43.71	67.74	1.00	93.73	29.70
369	9,393.96	818.61	36.62	51.01	1.00	75.66	34.18
370	33,203.75	2,579.29	42.81	47.52	1.00	68.84	23.42
371	36,604.29	2,871.28	42.45	42.32	1.00	66.19	11.57
372	4,181.32	439.74	29.70	61.77	1.00	83.16	29.02
373	16,518.87	1,264.54	43.27	50.77	1.00	72.21	16.72
374	4,281.93	493.15	24.03	56.28	1.00	60.59	17.87
375	5,757.97	525.73	33.01	62.32	1.00	70.89	8.20
376	2,782.11	378.99	14.84	72.23	1.00	32.15	36.09
377	4,036.70	698.55	10.07	48.23	1.00	32.91	67.62
378	2,303.70	281.90	22.25	64.08	1.00	42.07	16.58
379	2,476.25	443.93	8.01	55.03	1.00	34.55	74.56
380	25,948.65	573.15	43.27	15.08	1.00	54.98	31.26
381	8,328.28	183.17	44.03	67.31	1.00	93.38	17.30
382	13,687.51	314.72	40.10	67.91	1.00	73.04	37.70
383	25,778.87	629.86	35.72	50.04	1.00	80.97	29.51
384	16,329.53	409.14	33.96	55.86	1.00	78.00	22.69
385	11,482.72	980.28	14.72	59.72	1.00	99.07	46.40
386	5,878.10	310.03	45.99	80.30	0.00	98.80	25.37
387	21,110.21	1,422.71	31.86	66.39	0.00	97.84	25.51
388	3,460.95	103.98	39.25	78.13	0.00	97.38	8.03
389	165,081.60	10,723.73	38.20	38.90	1.00	87.28	64.27
390	3,609.61	256.78	32.89	69.52	1.00	94.47	3.98
391	3,008.80	160.58	55.08	54.30	0.00	98.42	9.82
392	9,696.31	568.93	40.51	51.38	1.00	98.09	29.12
393	9,562.88	312.87	40.60	67.01	0.00	97.10	22.85
394	10,370.87	533.73	44.22	46.84	0.00	96.74	17.62
395	268,036.66	17,431.49	37.50	61.59	1.00	92.97	56.36
396	20,360.89	1,406.36	36.34	59.94	1.00	98.92	40.41
397	308,851.49	20,425.31	36.65	51.09	1.00	92.28	67.77
398	7,426.00	490.53	38.42	68.61	1.00	99.74	11.53
399	14,737.27	978.79	37.98	66.38	1.00	99.53	29.34
400	370,845.89	25,087.79	35.31	46.13	1.00	79.52	72.16
401	5,123.61	405.11	27.60	45.98	1.00	93.74	31.93
402	18,409.50	1,359.46	31.13	38.44	1.00	95.21	39.83
403	12,526.34	922.94	31.25	53.48	1.00	96.73	47.07
404	8,411.60	763.59	19.69	70.47	0.00	98.50	41.11
405	21,137.26	1,546.01	31.82	36.08	0.00	69.34	38.27
406	2,861.43	101.53	29.38	86.50	0.00	99.96	25.22
407	16,808.05	1,277.15	31.69	61.29	1.00	95.73	44.64
408	465,327.87	32,442.42	33.82	57.21	1.00	60.40	58.69
409	10,731.64	1,692.46	4.19	56.44	1.00	83.42	53.27
410	53,585.52	5,209.88	16.90	47.87	1.00	72.51	69.79
411	76,734.02	7,905.81	14.90	50.05	1.00	80.07	55.06
412	15,440.70	921.39	49.84	37.42	1.00	81.65	41.19
413	3,648.48	512.54	6.58	56.12	0.00	87.75	32.89
414	8,688.98	790.11	20.06	54.73	0.00	94.69	22.53
415	8,144.32	691.95	24.87	47.62	0.00	74.59	12.68
416	6,861.91	562.14	24.87	41.19	1.00	98.39	14.35
417	7,644.36	772.53	17.02	65.00	1.00	93.39	41.23
418	8,973.90	799.97	22.73	52.34	1.00	65.85	16.53
419	25,086.06	3,259.26	8.12	64.46	1.00	85.16	67.05
420	5,554.98	619.82	13.08	69.94	1.00	76.36	8.53
421	2,673.61	224.04	21.12	35.20	1.00	49.06	4.91
422	21,293.34	2,028.66	18.59	52.60	1.00	89.48	42.99
423	4,852.73	400.31	23.65	46.31	1.00	78.54	23.66
424	12,253.97	807.68	39.11	42.86	1.00	17.96	21.00
425	9,234.25	359.48	18.70	66.29	0.00	83.24	12.69
426	27,117.87	2,268.64	23.56	37.62	1.00	59.19	27.43
427	84,907.30	9,246.52	12.95	33.72	1.00	68.50	52.81
428	1,833.23	331.08	0.44	66.35	1.00	90.21	44.09
429	18,791.94	2,313.05	9.67	41.68	1.00	78.06	72.22
430	2,201.17	152.84	11.96	47.26	0.00	88.46	28.86
431	19,202.10	1,035.82	61.25	37.85	1.00	19.55	0.00
432	1,905.40	356.16	0.39	59.37	0.00	63.49	27.88
433	4,737.81	688.67	6.04	62.20	1.00	80.52	44.11
434	4,141.35	249.71	24.48	46.62	1.00	80.49	15.95
435	13,242.09	1,156.68	21.41	42.92	1.00	66.33	28.95
436	8,246.02	787.86	18.48	38.36	1.00	57.07	31.81
437	11,535.55	890.06	27.12	23.47	1.00	32.34	30.52
438	3,403.27	199.32	51.25	44.66	0.00	66.58	0.00
439	8,271.39	1,284.38	4.85	61.22	1.00	81.28	56.01
440	3,186.18	253.70	28.41	28.12	1.00	50.10	33.72
441	5,053.52	347.58	36.48	50.41	1.00	80.75	17.59
442	1,653.22	107.71	42.59	55.28	0.00	89.69	22.88
443	24,994.35	2,191.63	22.72	43.92	1.00	71.02	33.99
444	4,018.79	514.76	9.53	43.55	0.00	55.34	14.27
445	11,254.46	893.25	28.81	39.16	1.00	53.12	37.17
446	5,777.29	376.43	42.29	47.08	1.00	88.75	26.67
447	5,300.77	470.13	21.75	29.88	1.00	49.08	27.91
448	5,366.64	588.52	13.15	34.80	1.00	43.10	23.27
449	8,995.19	799.69	22.76	33.25	1.00	51.65	33.17

450	5,670.62	429.37	31.82	59.76	1.00	65.99	1.96
451	7,660.18	521.17	38.65	34.87	1.00	82.72	11.12
452	5,504.55	359.38	36.18	53.88	0.00	84.12	17.44
453	15,040.62	956.90	42.39	30.07	1.00	79.94	10.57
454	22,028.23	1,442.12	40.51	28.48	1.00	86.43	46.94
455	3,273.55	280.82	20.77	46.02	1.00	82.45	35.82
456	1,507.88	131.43	18.06	62.28	1.00	79.32	29.91
457	12,359.45	696.24	40.14	48.44	0.00	90.28	12.60
458	20,311.11	1,258.86	39.39	39.04	1.00	91.03	15.04
459	6,205.95	444.50	31.57	31.49	1.00	87.72	17.68
460	4,334.67	271.75	43.43	59.62	0.00	87.44	10.33
461	3,473.57	210.33	46.44	47.89	1.00	93.83	4.31
462	11,026.47	497.04	48.53	38.79	0.00	94.49	6.25
463	5,651.76	435.69	20.82	53.77	1.00	80.59	2.31
464	5,397.18	526.30	13.61	89.84	0.00	93.33	26.73
465	3,713.75	275.61	28.79	57.62	1.00	92.13	22.20
466	8,491.59	591.57	34.32	40.54	1.00	84.93	6.55
467	3,413.28	114.07	46.90	59.34	0.00	89.57	0.00
468	7,969.86	775.47	15.62	73.80	1.00	89.34	32.59
469	1,212.95	94.98	29.58	80.83	0.00	89.52	0.00
470	6,919.23	390.80	55.98	34.76	1.00	47.92	0.00
471	5,481.32	260.08	39.39	56.40	0.00	94.75	3.69
472	8,929.09	756.85	22.17	40.24	1.00	45.48	11.72
473	8,131.71	557.89	36.50	48.54	1.00	84.94	2.45
474	20,646.67	1,435.78	34.67	42.00	1.00	45.68	27.26
475	19,529.90	1,517.58	27.78	49.46	1.00	71.50	23.93
476	6,374.58	416.96	40.63	47.67	1.00	71.24	1.55
477	2,777.51	179.80	42.48	69.86	1.00	39.92	0.00
478	3,761.29	248.87	39.74	49.80	1.00	34.11	2.24
479	1,611.24	127.11	28.69	80.13	1.00	83.64	40.72
480	36,600.94	766.86	46.64	35.78	1.00	35.36	8.78
481	46,825.33	2,662.89	49.79	49.45	0.00	97.43	3.69
482	165,554.84	10,751.21	38.23	45.27	1.00	93.57	40.93
483	8,023.22	460.00	43.29	52.34	0.00	88.44	0.00
484	8,869.33	314.22	17.57	67.93	0.00	92.82	41.11
485	8,725.74	186.35	45.91	70.33	0.00	52.09	1.94
486	5,453.00	189.97	25.00	55.32	0.00	66.29	33.65
487	5,822.02	288.88	71.13	67.24	0.00	96.26	0.00
488	4,785.73	274.01	55.81	53.52	0.00	93.31	1.30
489	18,816.72	1,040.70	50.76	62.25	0.00	93.96	0.00
490	9,993.26	320.03	30.12	75.10	0.00	25.26	9.04
491	9,232.46	337.17	16.55	51.87	1.00	79.52	21.10
492	10,125.44	228.33	40.74	62.42	0.00	88.23	10.57
493	27,764.19	1,991.58	19.43	54.59	1.00	7.73	24.83
494	3,430.36	287.78	18.87	47.78	0.00	83.51	38.29
495	14,234.70	1,617.44	11.90	44.71	0.00	28.66	24.51
496	7,643.91	284.98	15.54	52.29	1.00	70.46	17.33
497	8,331.18	547.40	39.00	32.25	0.00	90.67	3.43
498	54,585.02	3,575.28	37.16	34.27	1.00	91.32	23.17
499	16,609.10	1,168.62	33.03	45.66	1.00	85.98	10.57
500	11,903.56	839.91	33.71	38.48	0.00	76.75	15.50
501	4,088.59	274.84	36.10	69.24	0.00	97.84	27.59
502	48,062.92	3,179.46	36.32	35.84	1.00	94.66	15.41
503	14,485.52	931.72	38.39	57.20	1.00	4.59	0.69
504	10,414.83	522.96	44.97	60.56	0.00	4.29	0.00
505	32,377.11	2,368.73	21.53	42.55	1.00	11.08	26.57
506	2,772.94	414.41	2.71	53.20	0.00	78.17	50.25
507	3,832.47	206.14	66.45	76.01	0.00	0.00	0.00
508	47,496.92	949.84	50.77	43.02	0.00	25.61	9.44
509	11,614.81	422.87	54.69	55.68	0.00	10.10	6.63
510	84,266.50	1,738.78	48.10	42.74	1.00	21.70	21.94
511	10,622.56	727.08	34.83	38.24	0.00	84.16	19.08
512	14,191.00	267.46	56.43	56.41	0.00	51.47	9.97
513	23,108.16	1,491.68	36.96	50.41	1.00	86.91	2.47
514	2,461.37	210.31	18.75	42.47	0.00	89.38	13.75
515	3,643.19	180.90	69.23	53.23	0.00	96.12	0.00
516	5,663.39	514.59	26.29	41.65	0.00	80.55	36.29
517	9,839.75	274.04	27.41	55.18	1.00	83.91	27.43
518	11,817.13	732.14	53.43	57.36	0.00	3.81	1.62
519	11,327.18	245.31	43.82	64.58	0.00	73.69	3.46
520	79,170.14	5,716.29	26.72	37.02	1.00	12.89	27.73
521	6,290.22	349.01	48.56	60.93	0.00	99.04	0.00
522	238,585.56	15,563.96	37.80	46.06	1.00	90.56	29.80
523	3,752.83	352.41	20.55	48.74	0.00	35.22	6.69
524	8,418.12	662.89	33.43	63.02	1.00	5.04	0.00
525	4,709.20	374.79	36.33	50.43	0.00	2.20	9.59
526	15,544.27	1,023.19	39.01	57.13	1.00	94.36	31.43
527	27,190.28	860.99	22.34	36.70	1.00	47.53	21.23
528	5,695.09	284.02	70.56	67.21	0.00	91.71	0.00
529	12,175.76	385.64	22.46	66.72	1.00	90.81	25.03
530	6,050.64	330.79	56.76	59.71	0.00	92.42	8.45
531	6,019.00	266.94	51.16	69.12	0.00	0.00	2.14
532	9,837.12	216.27	52.20	66.47	0.00	1.09	2.53
533	6,250.65	381.14	59.13	63.36	0.00	1.64	0.00
534	9,659.61	280.26	25.99	49.76	1.00	62.36	8.57
535	16,961.14	1,128.14	38.13	38.13	0.00	83.29	19.30
536	13,632.94	1,251.10	18.63	49.94	0.00	84.32	24.91
537	2,625.16	233.09	23.19	45.05	0.00	19.90	14.43
538	32,865.04	645.01	53.89	44.46	0.00	53.97	0.00
539	9,849.75	764.29	26.36	37.85	0.00	81.95	20.57
540	41,381.45	849.65	49.41	66.93	0.00	80.26	26.00

541	6,560.17	541.01	28.68	54.40	1.00	7.08	0.00
542	309,009.98	20,435.17	36.69	39.19	1.00	88.22	36.29
543	1,923.88	225.80	6.52	36.44	0.00	36.48	29.00
544	6,742.48	212.15	30.75	43.66	0.00	15.00	13.57
545	15,883.57	370.91	45.99	53.34	0.00	36.73	10.29
546	1,423.69	145.86	12.92	44.13	0.00	22.34	6.44
547	51,356.95	1,127.48	43.62	52.83	1.00	78.39	11.47
548	3,609.38	103.34	25.03	46.84	0.00	61.61	0.00
549	8,195.31	285.73	18.83	74.14	0.00	87.81	10.37
550	3,244.83	289.53	21.92	48.04	0.00	90.83	41.84
551	3,835.19	282.85	30.26	45.31	0.00	92.64	6.16
552	1,055.08	50.45	7.79	74.86	0.00	89.34	12.74
553	46,194.19	3,452.79	29.84	46.93	1.00	53.41	40.27
554	19,892.94	1,667.33	24.39	32.97	0.00	23.06	37.25
555	5,240.47	157.53	33.70	54.39	0.00	47.74	21.52
556	3,634.99	195.03	60.26	42.69	0.00	84.99	1.62
557	3,419.51	429.83	9.11	57.25	0.00	80.62	28.12
558	38,221.73	1,434.81	25.02	66.03	1.00	82.48	32.01
559	3,381.35	279.79	28.15	54.67	0.00	4.85	1.66
560	6,766.93	406.64	61.75	51.00	0.00	7.30	0.00
561	370,586.10	25,040.63	35.40	37.78	1.00	81.85	49.02
562	4,677.87	306.36	36.83	46.87	0.00	89.78	3.84
563	13,792.99	1,141.73	23.85	57.42	0.00	93.66	41.84
564	8,851.31	435.73	8.56	79.12	1.00	94.15	38.87
565	31,268.75	2,464.61	25.94	45.77	1.00	82.54	48.46
566	4,348.97	243.54	55.82	53.81	0.00	93.04	9.19
567	16,057.18	1,388.33	21.94	48.54	1.00	95.68	48.55
568	7,417.62	478.96	38.49	46.24	1.00	8.65	10.08
569	6,110.36	450.24	32.34	54.58	0.00	79.83	18.40
570	12,197.46	361.75	26.47	40.08	1.00	43.32	40.79
571	1,130.76	130.47	0.50	64.06	0.00	46.18	29.29
572	6,282.35	413.19	41.34	64.74	0.00	88.59	13.89
573	3,546.47	296.08	30.99	49.71	0.00	2.42	8.15
574	9,376.20	801.16	24.61	62.75	1.00	84.78	45.49
575	38,250.38	3,331.13	21.51	65.16	1.00	55.32	51.22
576	11,399.03	1,281.22	13.24	47.87	0.00	90.31	52.20
577	5,386.44	206.83	15.95	29.99	1.00	45.62	29.75
578	4,795.91	461.46	21.42	61.13	0.00	28.60	31.01
579	11,195.56	1,026.46	5.91	87.41	1.00	98.61	40.90
580	50,374.77	4,655.88	18.87	45.96	1.00	80.29	39.71
581	8,555.48	783.17	19.91	42.11	1.00	76.81	1.89
582	3,454.35	257.45	37.76	40.54	1.00	5.10	10.92
583	4,536.65	466.53	18.75	55.51	1.00	72.70	1.95
584	7,852.04	395.11	8.69	73.88	1.00	84.53	54.96
585	2,244.98	159.95	40.54	57.53	0.00	7.56	7.15
586	1,920.65	162.92	24.43	52.46	0.00	80.25	25.88
587	2,972.62	287.21	17.53	54.83	0.00	5.38	15.50
588	5,472.53	545.40	20.77	64.63	0.00	85.27	14.79
589	4,979.68	489.42	17.06	49.72	0.00	57.26	37.81
590	10,985.13	1,035.31	26.09	56.85	1.00	82.00	30.49
591	7,018.36	642.59	20.64	49.67	0.00	13.67	19.23
592	2,550.27	215.33	25.16	64.90	0.00	94.86	1.89
593	6,427.34	676.93	17.43	76.51	0.00	91.28	28.31
594	2,166.98	181.44	27.57	58.23	0.00	44.14	17.87
595	3,366.31	277.16	24.84	42.52	0.00	28.02	17.79
596	5,984.38	211.32	18.13	35.72	1.00	53.09	33.40
597	2,248.48	204.95	19.44	53.24	0.00	42.70	15.97
598	3,512.51	354.23	22.35	66.07	0.00	93.83	16.40
599	6,474.03	539.19	2.23	90.44	1.00	99.55	27.51
600	4,626.83	551.17	8.89	70.88	1.00	94.09	22.02
601	3,812.75	292.70	42.26	68.56	0.00	95.73	7.82
602	9,299.30	969.22	10.16	88.89	1.00	98.70	47.20
603	2,492.64	269.04	16.78	75.81	1.00	95.71	16.51
604	1,508.06	160.44	19.39	92.72	1.00	99.64	17.30
605	4,325.15	422.44	12.48	59.75	1.00	68.93	29.80
606	5,887.63	774.88	5.24	97.07	1.00	99.40	45.33
607	4,835.37	496.27	10.63	67.33	1.00	85.07	7.62
608	18,255.60	1,702.03	19.90	71.29	0.00	93.48	3.50
609	3,292.94	317.86	21.77	73.72	0.00	87.17	19.91
610	4,001.31	301.74	44.78	69.26	1.00	82.38	1.67
611	10,289.09	747.21	43.43	50.56	1.00	62.06	5.62
612	1,619.98	139.09	18.51	63.60	1.00	70.26	0.00
613	12,903.43	1,475.61	12.59	67.10	0.00	92.72	23.58
614	1,467.50	173.88	7.23	95.14	0.00	98.47	9.88
615	6,889.45	824.37	10.66	91.33	0.00	98.71	11.70
616	37,909.51	3,941.44	15.39	74.78	1.00	91.90	18.06
617	8,043.93	1,126.06	5.49	92.61	0.00	99.70	59.95
618	4,807.71	465.44	21.92	58.47	1.00	72.38	21.27
619	20,180.61	2,362.93	6.35	60.14	1.00	92.17	30.65
620	9,184.42	772.39	29.90	64.40	1.00	81.90	12.30
621	15,190.62	1,826.36	6.36	79.56	0.00	91.70	43.96
622	1,399.59	153.37	6.34	85.98	1.00	85.73	0.00
623	3,378.09	241.83	8.18	58.53	0.00	73.90	21.03
624	3,307.77	275.27	9.67	79.62	1.00	94.01	15.88
625	3,972.88	453.95	11.81	88.84	0.00	93.47	22.82
626	12,699.15	1,418.41	4.43	54.49	0.00	82.20	35.78
627	5,727.47	812.96	7.71	88.14	1.00	97.01	17.33
628	39,004.53	4,054.61	7.30	81.67	1.00	92.92	32.48
629	4,844.11	621.72	6.18	90.04	1.00	100.00	17.18
630	8,659.76	779.48	8.88	82.35	0.00	90.90	54.73
631	8,659.91	650.63	14.06	54.67	1.00	67.97	4.94

632	24,544.99	2,247.69	14.13	46.35	1.00	38.88	14.29
633	27,179.14	2,840.33	7.15	76.15	1.00	88.89	3.01
634	1,778.05	196.14	18.61	91.02	1.00	100.00	17.08
635	1,958.45	256.72	14.72	92.38	1.00	100.00	27.74
636	9,604.06	1,112.16	9.29	57.32	1.00	83.04	6.84
637	1,549.04	184.32	5.35	76.13	1.00	94.81	16.21
638	4,678.79	364.18	13.55	48.91	1.00	74.32	25.89
639	813.01	128.98	4.95	98.64	1.00	99.54	25.55
640	48,528.78	4,998.17	8.72	98.67	1.00	74.53	8.29
641	51,131.83	5,272.02	8.96	81.44	1.00	94.22	36.20
642	44,753.02	4,692.52	7.68	93.20	1.00	93.48	13.40
643	3,267.38	247.12	13.36	69.98	1.00	78.77	0.00
644	3,454.28	312.30	8.29	75.52	1.00	89.39	18.50
645	5,446.69	450.19	9.36	72.83	1.00	95.79	20.01
646	5,563.43	414.67	9.95	64.98	0.00	91.14	22.35
647	11,142.97	873.29	6.95	79.73	0.00	97.77	25.01
648	14,609.34	1,797.35	4.63	93.61	0.00	98.99	56.53
649	2,397.23	280.70	16.40	94.38	1.00	99.07	70.20
650	1,606.22	130.08	15.53	79.74	0.00	99.86	4.49
651	3,131.89	303.00	6.66	68.06	1.00	85.58	29.49
652	3,647.25	427.85	12.69	84.30	0.00	99.31	45.96
653	1,470.69	143.16	7.22	94.19	1.00	99.34	0.00
654	6,609.61	623.95	12.76	69.75	0.00	84.87	29.35
655	13,121.60	936.76	11.11	54.37	0.00	73.56	18.91
656	11,082.60	744.58	10.75	51.29	0.00	66.37	7.42
657	7,956.36	835.38	11.38	75.22	0.00	97.19	46.30
658	1,756.04	226.59	7.59	88.36	1.00	95.48	54.96
659	5,269.60	579.06	6.71	81.92	1.00	91.64	17.73
660	10,406.48	1,039.74	8.68	76.36	0.00	97.60	41.53
661	3,235.14	209.59	8.99	79.25	0.00	94.59	27.38
662	6,704.61	682.56	7.12	75.70	1.00	91.39	20.87
663	17,353.97	1,734.93	10.56	70.85	1.00	87.50	29.96
664	20,641.14	1,845.95	9.13	64.17	1.00	92.39	36.03
665	26,365.17	2,626.01	12.18	52.74	1.00	89.71	30.19
666	2,915.67	291.61	16.87	90.21	1.00	98.75	2.12
667	3,310.67	301.67	11.05	80.76	0.00	99.38	10.65
668	613.56	91.41	11.72	89.45	1.00	100.00	48.40
669	19,394.07	1,536.68	9.85	56.67	1.00	88.98	33.96
670	2,976.41	173.67	6.49	65.45	1.00	99.83	26.72
671	4,843.49	462.45	12.06	73.35	1.00	97.92	27.25
672	977.07	115.67	14.18	82.33	1.00	96.72	13.06
673	21,477.80	1,693.12	10.37	74.04	1.00	90.53	38.14
674	6,118.79	484.82	8.82	57.71	1.00	87.01	14.77
675	3,867.40	363.51	11.00	79.72	1.00	99.09	33.77
676	2,185.51	189.65	21.66	90.28	1.00	99.88	7.28
677	5,321.66	523.44	9.98	86.68	1.00	98.77	29.42
678	3,727.10	305.41	11.40	72.52	1.00	97.69	11.24
679	2,640.39	137.82	16.81	75.67	1.00	98.88	0.00
680	2,426.01	246.66	10.85	86.98	1.00	93.33	31.90
681	7,440.36	609.85	14.30	73.00	1.00	90.29	12.24
682	1,795.53	170.41	24.71	97.88	1.00	99.10	20.52
683	1,651.21	182.88	15.74	94.73	1.00	98.56	18.08
684	3,053.53	300.52	9.39	78.67	1.00	98.78	13.78
685	7,158.84	600.67	26.07	58.75	1.00	90.23	14.86
686	5,360.26	638.60	6.02	86.66	1.00	95.85	36.38
687	5,238.76	435.39	8.40	95.19	1.00	99.82	15.69
688	5,912.80	512.48	33.93	87.23	1.00	92.35	11.71
689	2,436.01	184.22	44.16	91.92	1.00	96.35	3.59
690	2,705.09	207.38	16.53	90.10	1.00	100.00	1.58
691	3,435.46	468.63	3.72	78.11	1.00	88.67	41.74
692	2,688.72	197.77	15.61	81.67	1.00	99.64	6.29
693	5,394.27	386.84	11.02	79.37	1.00	99.56	0.00
694	1,819.59	160.82	14.44	81.06	1.00	98.38	24.14
695	91,124.70	9,203.90	5.65	39.39	1.00	56.29	43.61
696	57,918.85	6,689.40	5.47	46.12	1.00	61.88	56.45
697	29,499.79	3,171.01	6.63	42.94	1.00	72.05	51.60
698	14,594.16	1,351.94	19.37	52.85	1.00	77.11	5.10
699	13,760.00	1,201.97	21.04	54.61	0.00	74.56	7.69
700	404,718.56	27,579.00	12.25	58.30	1.00	73.47	57.83
701	16,754.09	295.38	11.25	45.71	1.00	31.14	4.13
702	5,211.56	85.42	6.47	50.29	1.00	14.61	2.31
703	12,937.53	264.51	13.12	57.78	1.00	41.91	1.88
704	7,192.68	130.41	13.08	77.90	1.00	60.74	0.00
705	23,083.99	510.13	6.67	50.42	1.00	46.05	31.66
706	16,642.14	410.50	8.43	76.86	1.00	80.64	33.53
707	3,702.67	84.10	11.00	81.78	1.00	90.05	0.00
708	9,403.97	154.42	5.75	72.01	1.00	66.22	1.06
709	4,393.94	145.66	49.54	88.71	1.00	69.54	0.00
710	2,959.71	83.89	12.43	86.46	1.00	78.63	14.42
711	9,616.17	227.94	12.77	90.05	1.00	89.87	12.49
712	6,838.65	206.63	12.90	63.94	1.00	72.33	13.52
713	4,749.41	117.38	15.71	79.29	1.00	54.88	2.88
714	21,925.23	794.74	19.19	76.98	1.00	22.70	12.47
715	2,824.11	68.17	12.42	96.36	1.00	56.31	2.20
716	24,106.75	699.30	13.87	67.09	1.00	26.31	4.07
717	8,081.63	240.95	10.27	80.68	1.00	69.00	20.68
718	6,236.78	191.75	10.05	59.69	1.00	95.64	0.00
719	9,554.68	615.32	13.22	50.02	1.00	86.17	11.49
720	6,616.30	150.34	7.90	62.94	1.00	88.45	6.99
721	8,872.66	330.21	8.94	60.95	1.00	89.84	38.61
722	7,675.27	125.67	7.62	65.69	1.00	45.93	4.89

723	9,963.88	624.07	12.94	50.62	1.00	96.22	0.00
724	75,384.50	4,916.10	10.62	46.41	1.00	95.78	18.57
725	5,280.19	282.73	14.39	60.74	1.00	93.60	0.00
726	7,816.72	237.33	10.15	45.94	1.00	35.17	0.00
727	20,466.84	525.96	13.72	48.82	1.00	31.24	11.71
728	8,736.79	152.56	14.63	66.93	1.00	19.44	1.54
729	2,883.29	156.51	16.11	63.63	1.00	98.48	0.00
730	15,478.31	366.42	12.94	58.10	1.00	56.88	30.09
731	24,236.07	526.00	9.42	86.01	1.00	85.82	12.66
732	14,667.74	363.04	22.84	84.98	1.00	57.29	0.00
733	10,846.97	388.21	8.47	61.12	1.00	97.26	51.00
734	41,854.75	894.85	10.18	86.14	1.00	69.82	6.01
735	69,333.68	4,580.43	10.78	37.42	1.00	90.15	29.31
736	4,688.37	248.62	9.43	76.30	1.00	99.87	4.25
737	18,291.72	433.77	22.12	85.72	1.00	68.52	0.00
738	14,500.86	958.59	13.79	46.94	1.00	98.39	0.00
739	27,561.71	582.41	9.91	71.00	1.00	29.48	2.13
740	3,315.27	258.05	7.75	51.49	1.00	96.60	0.00
741	3,019.62	220.32	6.88	51.51	1.00	95.26	19.02
742	14,733.09	917.72	12.10	57.23	1.00	98.80	23.36
743	8,663.52	173.27	22.44	80.02	1.00	58.20	0.00
744	44,069.29	1,087.16	12.55	52.02	1.00	24.06	10.13
745	12,694.45	388.37	8.88	58.13	1.00	82.70	49.26
746	21,244.18	411.48	10.59	56.15	1.00	37.55	0.00
747	32,009.53	2,226.16	8.68	57.38	1.00	94.35	4.52
748	30,503.96	783.36	23.15	53.37	1.00	34.24	19.16
749	40,896.79	808.63	10.43	67.32	1.00	13.63	0.00
750	6,961.73	202.91	5.42	77.03	1.00	96.28	30.65
751	37,712.93	699.76	11.33	46.67	1.00	27.80	5.54
752	13,399.88	779.74	1.08	89.00	1.00	99.87	74.20
753	7,111.63	477.69	8.98	71.74	1.00	89.34	0.00
754	37,562.92	779.23	20.31	66.82	1.00	33.61	0.00
755	3,001.06	266.24	5.90	47.99	1.00	95.53	21.70
756	38,167.58	784.04	20.01	68.62	1.00	44.86	0.00
757	39,981.54	815.72	11.73	63.69	1.00	10.66	0.00
758	5,149.12	107.50	22.41	76.68	1.00	57.82	2.01
759	11,323.94	199.28	21.01	63.86	1.00	8.31	0.00
760	19,490.97	328.95	11.39	76.89	1.00	82.27	2.39
761	31,791.09	527.43	13.88	80.89	1.00	31.64	0.00
762	6,865.48	118.59	17.21	57.28	1.00	16.55	0.00
763	48,616.07	1,196.36	13.29	66.50	1.00	15.30	29.04
764	42,073.59	1,000.34	21.46	73.95	1.00	11.94	3.19
765	22,518.82	643.13	6.21	73.79	1.00	47.12	26.94
766	21,853.75	489.22	16.23	38.91	1.00	34.38	45.14
767	16,983.50	263.54	13.48	78.30	1.00	40.15	0.00
768	56,638.91	1,558.27	8.69	54.63	1.00	32.00	38.98
769	9,070.39	210.26	25.84	60.17	1.00	9.14	0.00
770	6,314.12	276.65	3.56	66.61	1.00	93.01	36.00
771	27,575.75	720.66	8.32	58.02	1.00	75.27	6.05
772	83,122.62	1,736.77	19.68	62.08	1.00	31.62	2.09
773	111,578.58	2,822.00	8.58	57.29	1.00	37.55	29.57
774	17,913.97	1,020.70	9.48	70.47	1.00	91.60	6.29
775	193,264.21	4,671.30	10.57	53.96	1.00	28.62	29.04
776	52,637.69	3,129.89	6.68	81.28	1.00	99.23	69.72
777	22,827.44	542.32	6.52	33.67	1.00	21.64	56.45
778	3,167.20	90.91	5.18	62.79	1.00	43.46	17.77
779	6,234.04	141.94	7.05	52.61	1.00	78.70	41.29
780	3,950.23	132.78	4.44	42.57	1.00	52.90	50.55
781	6,501.64	158.25	18.70	73.37	1.00	10.14	6.67
782	14,764.53	374.26	10.96	52.13	1.00	24.45	27.65
783	10,751.96	246.54	33.21	62.05	1.00	77.56	0.00
784	20,202.36	438.66	26.42	60.06	1.00	16.76	2.16
785	21,824.16	384.28	11.76	55.49	1.00	61.19	1.19
786	12,409.34	353.73	4.76	65.44	1.00	27.22	23.93
787	24,165.44	578.85	21.13	57.38	1.00	35.65	43.03
788	7,542.50	105.91	3.11	56.86	1.00	0.00	0.00
789	5,992.77	99.79	9.38	60.69	1.00	71.15	4.60
790	10,366.79	259.37	33.83	60.89	1.00	22.82	1.75
791	37,968.75	727.87	16.64	48.10	1.00	26.48	1.92
792	22,150.49	631.81	11.35	53.04	1.00	38.13	24.41
793	9,919.02	187.45	20.06	67.46	1.00	51.74	0.00
794	2,994.13	85.36	12.72	37.06	1.00	29.19	21.83
795	9,420.00	331.47	4.59	84.02	1.00	71.24	50.80
796	4,137.59	81.60	10.29	66.99	1.00	67.97	22.24
797	7,996.51	193.20	20.38	57.01	1.00	35.47	30.04
798	15,026.22	300.84	11.20	61.40	1.00	64.89	21.37
799	8,824.07	277.58	10.89	76.96	1.00	74.25	30.05
800	4,645.34	92.51	14.71	62.47	1.00	69.77	4.34
801	9,036.71	176.10	11.23	70.51	1.00	70.46	13.19
802	4,942.25	92.99	9.35	57.13	1.00	34.34	2.16
803	5,844.86	135.21	30.56	55.57	1.00	71.62	23.53
804	27,970.65	483.63	15.61	44.49	1.00	42.08	0.00
805	15,629.00	317.68	9.29	79.17	1.00	83.14	17.24
806	26,477.39	562.03	16.28	49.38	1.00	28.65	8.37
807	3,548.21	120.54	5.47	68.84	1.00	80.96	25.66
808	37,400.63	833.18	20.06	56.55	1.00	25.17	9.38
809	8,189.53	200.16	4.68	52.65	1.00	66.45	0.00
810	13,419.45	310.13	15.44	55.15	1.00	10.87	12.77
811	59,534.48	1,140.35	11.42	55.90	1.00	17.61	16.14
812	13,405.53	226.51	10.69	53.37	1.00	25.31	4.22
813	36,255.72	639.89	12.15	63.66	1.00	9.27	0.00

814	44,880.66	847.12	9.47	57.60	1.00	43.47	5.42
815	17,281.98	263.45	7.79	54.62	1.00	70.67	0.00
816	6,743.71	40.75	2.95	65.30	1.00	67.74	0.00
817	11,068.25	202.81	14.80	41.87	1.00	47.27	0.00
818	208,310.16	5,315.39	12.09	57.86	1.00	38.61	33.14
819	19,285.31	368.83	14.15	46.20	1.00	39.65	0.00
820	78,050.24	1,566.14	11.19	49.09	1.00	17.52	24.38
821	4,415.46	105.98	19.56	67.17	1.00	28.41	0.00
822	5,686.05	117.14	19.98	80.36	1.00	77.39	3.92
823	15,814.32	245.20	8.47	67.39	1.00	76.35	0.00
824	203,695.31	5,137.48	11.19	59.83	1.00	12.70	43.49
825	105,877.71	2,087.11	23.57	53.41	1.00	30.10	23.83
826	9,417.93	172.94	8.84	85.06	1.00	83.21	20.11
827	11,841.06	252.02	14.94	56.75	1.00	60.54	18.25
828	3,262.34	67.88	2.51	86.65	1.00	90.67	21.98
829	8,312.54	172.54	11.14	56.16	1.00	13.49	5.50
830	9,583.25	197.36	14.21	94.32	1.00	97.35	8.73
831	11,761.90	251.59	10.91	89.03	1.00	91.82	7.00
832	8,047.06	172.15	4.53	76.66	1.00	34.75	33.23
833	7,067.32	141.49	11.45	70.96	1.00	67.73	31.55
834	11,970.62	203.50	4.47	67.09	1.00	90.26	3.95
835	95,750.11	2,788.40	10.94	52.21	1.00	38.85	57.41
836	15,052.68	330.93	9.18	72.61	1.00	69.30	19.23
837	16,511.38	404.23	8.57	74.89	1.00	65.66	33.81
838	9,184.05	223.07	29.10	61.52	1.00	27.70	12.74
839	17,482.18	305.79	18.93	53.76	1.00	28.00	16.11
840	12,264.60	287.18	8.44	71.22	1.00	18.75	28.52
841	479,149.30	11,180.75	12.98	51.75	1.00	21.58	36.98
842	113,315.52	3,184.77	8.40	56.89	1.00	22.75	28.25
843	12,082.38	224.04	9.02	70.90	1.00	84.12	29.94
844	4,774.27	118.89	13.19	55.00	1.00	74.54	40.29
845	3,444.80	89.98	9.44	74.56	1.00	70.42	23.22
846	9,449.33	154.24	10.31	81.27	1.00	78.70	0.00
847	41,718.05	1,166.34	11.62	60.86	1.00	44.71	36.67
848	346,392.78	7,462.45	14.89	65.10	1.00	52.92	58.08
849	3,692.01	126.94	7.80	71.13	1.00	78.51	35.48
850	17,414.01	330.80	12.25	68.92	1.00	83.96	17.51
851	4,627.92	163.96	23.14	79.76	1.00	87.80	35.54
852	8,331.39	205.53	11.68	76.25	1.00	81.24	34.07
853	7,450.07	198.17	8.39	54.36	1.00	70.01	17.63
854	31,573.45	1,079.03	10.57	58.76	1.00	57.07	47.05
855	8,140.73	184.07	8.63	66.25	1.00	2.64	2.99
856	329,856.30	7,130.38	14.71	56.06	1.00	41.50	26.79
857	94,787.95	2,784.24	8.49	56.74	1.00	13.16	34.69
858	11,181.35	230.08	6.77	59.15	1.00	24.95	13.01
859	9,106.98	213.04	13.21	62.50	1.00	31.49	40.19
860	11,175.43	202.55	7.53	63.33	1.00	91.50	3.19
861	5,909.08	198.03	6.87	72.31	1.00	66.54	28.66
862	13,871.29	272.56	6.25	50.28	1.00	20.65	22.70
863	6,325.71	303.96	9.67	80.38	1.00	95.15	73.06
864	3,612.90	104.65	17.88	78.74	1.00	12.01	3.47
865	5,427.89	117.62	15.35	65.34	1.00	56.78	18.27
866	57,672.63	1,960.10	8.41	54.59	1.00	15.34	33.66
867	17,774.20	324.29	10.96	39.59	1.00	53.25	1.35
868	10,671.67	210.43	7.14	73.42	1.00	88.94	17.80
869	6,561.15	122.74	14.85	71.65	1.00	88.83	8.47
870	5,462.92	118.70	13.25	72.71	1.00	24.27	9.07
871	404,326.66	8,784.44	9.37	54.57	1.00	52.97	79.14
872	6,783.43	129.80	11.65	55.43	1.00	95.63	12.54
873	3,109.07	183.67	4.02	89.32	1.00	92.25	75.35
874	46,935.75	1,607.89	8.71	56.28	1.00	30.21	48.84
875	111,345.39	2,669.53	11.63	55.45	1.00	26.14	38.65
876	15,338.84	574.79	8.80	54.75	1.00	29.42	41.12
877	12,907.98	256.37	15.39	55.71	1.00	74.61	13.16
878	9,118.95	403.51	5.32	83.30	1.00	90.57	31.68
879	3,116.80	71.96	19.16	79.53	1.00	95.15	12.19
880	6,270.74	153.03	10.46	45.78	1.00	47.20	27.58
881	9,261.71	156.30	7.48	80.19	1.00	66.74	1.97
882	36,277.81	1,121.86	8.61	59.45	1.00	18.23	8.12
883	7,459.97	181.54	7.61	67.12	1.00	86.72	39.48
884	6,598.40	205.69	8.78	67.63	1.00	55.75	10.80
885	3,758.41	229.75	2.81	68.27	1.00	85.73	41.42
886	130,106.76	2,803.63	16.30	61.49	1.00	47.39	39.33
887	16,383.41	455.67	12.33	61.86	1.00	40.76	30.34
888	37,723.06	908.25	12.75	51.72	1.00	48.94	4.24
889	4,906.52	99.31	11.57	76.14	1.00	90.12	13.36
890	1,230.63	53.87	3.94	83.58	1.00	87.47	42.24
891	5,846.86	116.86	6.53	76.45	1.00	44.18	11.44
892	13,110.92	358.25	8.61	62.53	1.00	64.94	6.08
893	346,518.94	7,504.67	9.40	57.64	1.00	77.79	41.47
894	23,668.13	546.11	8.57	53.32	1.00	81.30	44.77
895	22,840.88	546.59	10.72	44.84	1.00	74.69	31.17
896	11,232.75	288.95	15.89	69.32	1.00	51.64	5.39
897	5,516.23	119.20	14.21	64.67	1.00	90.15	31.08
898	7,070.37	177.97	14.47	66.72	1.00	45.82	28.26
899	6,649.27	327.66	9.49	60.13	1.00	79.04	41.25
900	28,703.62	760.14	8.07	64.52	1.00	49.54	20.64
901	2,134.77	93.36	11.48	80.23	1.00	89.11	38.12
902	2,003.91	68.11	9.39	80.09	1.00	83.16	28.02
903	13,079.03	260.06	12.86	67.80	1.00	86.54	16.58
904	4,746.16	223.53	11.07	85.83	1.00	80.14	6.20

905	57,604.53	1,322.65	13.51	57.23	1.00	34.89	5.10
906	23,479.48	623.45	13.55	64.04	1.00	63.05	37.02
907	10,812.86	271.41	8.52	76.80	1.00	74.24	33.87
908	1,870.10	117.80	8.45	85.60	1.00	91.96	53.22
909	6,858.88	157.32	7.97	47.03	1.00	87.07	31.63
910	3,021.56	199.23	2.30	83.58	1.00	97.18	64.28
911	31,317.87	752.88	11.33	49.69	1.00	57.52	2.30
912	4,219.00	66.77	12.77	86.73	1.00	60.08	0.00
913	13,023.70	347.19	12.29	74.77	1.00	69.99	35.36
914	11,941.66	285.90	8.36	74.92	1.00	66.87	37.35
915	7,314.70	171.43	8.03	78.69	1.00	74.81	2.11
916	1,584.85	59.98	3.99	72.84	1.00	29.34	24.37
917	1,368.56	47.32	3.23	49.53	1.00	90.78	32.75
918	1,031,668.21	23,909.74	16.21	58.14	1.00	22.94	49.20
919	6,289.67	119.58	23.50	69.91	1.00	0.25	0.00
920	364,535.11	8,262.36	15.71	37.04	1.00	9.23	18.67
921	12,632.72	228.53	21.11	61.46	1.00	47.55	7.07
922	76,932.32	1,628.20	14.69	60.69	1.00	17.07	6.04
923	8,259.30	170.97	12.64	67.27	1.00	23.83	3.69
924	13,368.22	247.78	13.38	66.62	1.00	20.16	2.64
925	39,766.31	969.64	8.56	54.23	1.00	22.75	30.62
926	7,177.93	183.40	12.07	52.39	1.00	24.81	18.54
927	7,836.56	231.14	18.39	56.62	1.00	59.06	30.18
928	3,927.81	288.79	2.80	69.95	1.00	79.48	44.62
929	5,638.83	299.52	7.98	54.95	1.00	92.11	12.03
930	18,074.47	1,112.76	11.85	67.07	1.00	95.59	0.00
931	4,605.69	85.43	10.55	80.19	1.00	96.15	3.98
932	7,722.63	103.46	10.48	92.41	1.00	100.00	24.01
933	115,523.63	9,123.32	8.85	30.99	1.00	58.54	39.64
934	27,922.33	3,660.66	8.51	42.21	1.00	40.75	61.84
935	4,359.84	428.54	8.46	38.53	1.00	56.24	61.56
936	4,487.69	429.07	8.34	40.46	1.00	64.54	59.11
937	4,220.60	473.69	6.99	38.14	1.00	58.14	31.49
938	4,959.03	395.50	10.45	53.53	1.00	81.21	19.76
939	5,410.36	570.01	13.92	53.53	1.00	81.84	41.80
940	2,136.04	287.01	3.89	30.88	1.00	74.84	65.73
941	12,398.03	1,017.69	4.63	52.10	1.00	82.99	11.69
942	8,675.03	999.44	2.94	70.50	1.00	90.99	52.81
943	3,274.39	303.65	7.54	69.86	0.00	93.58	66.89
944	2,271.52	204.34	3.46	54.92	0.00	82.37	51.05
945	53,782.66	2,421.64	14.48	24.16	1.00	31.60	23.40
946	9,702.81	623.07	4.34	22.72	1.00	18.62	30.81
947	14,124.11	1,361.51	9.71	36.81	1.00	57.46	50.71
948	15,857.00	640.23	8.29	52.68	0.00	44.19	7.80
949	96,752.47	8,763.53	9.72	28.44	1.00	36.19	31.83
950	30,671.44	1,793.51	8.96	26.52	1.00	54.90	25.00
951	36,526.02	4,820.74	8.38	24.73	1.00	36.06	35.16
952	1,931.05	247.01	6.26	61.52	0.00	76.13	35.73
953	15,571.60	1,161.87	10.39	26.02	1.00	27.82	40.68
954	21,587.71	1,236.15	7.59	10.41	1.00	13.05	17.90
955	4,005.57	860.06	7.02	42.63	1.00	57.03	19.79
956	15,541.22	1,178.93	8.91	52.76	0.00	66.59	34.01
957	12,902.07	728.38	8.90	42.27	0.00	72.41	39.36
958	9,090.82	496.21	7.01	45.39	0.00	70.38	25.62
959	14,382.89	1,258.84	13.09	28.97	1.00	51.84	28.44
960	19,067.77	1,398.89	14.76	29.18	1.00	13.58	44.14
961	32,827.66	1,580.48	9.09	9.52	1.00	19.04	18.67
962	93,652.27	5,179.14	18.62	7.79	1.00	9.60	19.06
963	6,480.91	482.95	6.28	34.94	1.00	16.84	38.82
964	32,768.35	2,450.33	20.55	13.93	1.00	9.36	45.49
