# Supplementary material for: Using Structured Decision Making to Evaluate Wetland Restoration Opportunities in the Chesapeake Bay Watershed
Source: Environ Manage. 2022 Oct 8;70(6):950–64. doi: 10.1007/s00267-022-01725-5 (PMC9622542; doi:10.1007/s00267-022-01725-5)
Supplement: Supplementary file 3 — Electronic Supplemental Material [file 267_2022_1725_MOESM3_ESM.pdf]

Electornic Supplemental Material: Using structured decision making to evaluate wetland restoration opportunities in the Chesapeake Bay watershe
Journal: Environmental Management
Authors: David M. Martin, Amy D. Jacobs, Chase McLean, Michelle R. Canick, Kathleen Boomer
Correspondence: David M. Martin: David.Martin@tnc.org, (301) 897.8570 (Main), (301) 897.0858 (Fax)

Table S2 Criteria measures per alternative, 2025 Scenario

Alternative	TN	TP	AG	EHA	FP	WS	CF
1	2,211.92	585.56	1.84	92.98	1.00	97.89	46.20
2	1,719.87	227.83	7.43	99.06	1.00	100.00	72.63
3	3,347.82	584.45	13.06	97.79	1.00	99.18	32.43
4	3,785.14	518.42	17.79	90.16	1.00	97.79	24.22
5	1,836.63	526.08	2.88	95.90	1.00	99.50	43.42
6	1,182.23	288.27	5.07	93.90	1.00	99.33	45.36
7	695.38	117.83	0.27	99.47	1.00	100.00	67.98
8	2,267.20	374.79	16.91	85.58	1.00	96.29	61.25
9	4,878.15	660.32	15.93	97.22	1.00	98.89	53.25
10	4,960.52	708.01	12.20	95.21	1.00	99.30	80.01
11	1,297.78	312.34	4.66	98.75	1.00	93.55	50.68
12	1,560.85	536.65	1.76	93.16	1.00	99.87	56.63
13	3,442.41	402.03	43.88	88.20	1.00	99.50	5.65
14	1,107.43	280.50	10.55	93.11	1.00	99.17	34.57
15	5,791.64	685.34	16.75	82.68	1.00	88.30	22.22
16	886.32	134.09	2.64	88.19	1.00	99.88	36.45
17	1,163.28	324.52	8.43	86.21	1.00	99.26	51.49
18	3,878.11	641.51	0.63	95.10	1.00	98.62	77.43
19	4,698.45	661.66	6.56	90.58	1.00	96.44	71.42
20	7,066.41	998.27	5.82	93.16	1.00	99.82	52.61
21	1,648.68	126.55	55.71	59.77	1.00	86.57	7.62
22	2,311.01	173.29	56.40	63.95	1.00	88.26	8.78
23	12,001.54	1,641.79	10.47	92.15	1.00	98.64	53.25
24	8,142.43	903.28	16.85	86.49	1.00	96.46	41.47
25	1,602.93	180.08	22.37	45.83	1.00	63.33	27.40
26	13,256.71	1,129.33	44.17	84.33	1.00	95.34	24.35
27	7,410.42	933.21	35.93	64.45	1.00	98.79	7.46
28	1,436.95	161.98	21.35	97.42	1.00	100.00	33.73
29	3,300.20	693.81	3.93	94.35	1.00	99.82	70.43
30	1,976.11	672.83	0.64	81.33	1.00	94.36	53.34
31	29,521.24	4,955.12	17.23	93.20	1.00	99.90	67.08
32	1,191.83	132.96	22.52	92.36	1.00	99.09	39.77
33	1,851.51	233.66	14.76	88.39	1.00	95.26	35.06
34	2,363.07	233.91	26.47	76.27	1.00	91.06	37.07
35	1,587.87	388.57	9.55	93.27	1.00	97.79	26.86
36	5,443.83	569.29	18.31	83.37	1.00	95.59	54.39
37	2,865.11	44.61	79.55	53.20	1.00	99.99	2.53
38	1,501.72	181.08	3.35	87.16	1.00	98.21	85.60
39	8,804.11	791.52	39.37	81.01	1.00	89.81	47.88
40	1,109.70	226.88	18.06	91.73	1.00	100.00	18.58
41	10,540.90	849.48	50.25	89.84	1.00	98.57	18.94
42	11,153.90	1,486.29	25.76	89.98	1.00	99.72	34.46
43	5,462.53	476.53	41.57	82.81	1.00	99.95	3.47
44	43,276.39	3,917.63	38.15	82.06	1.00	99.07	30.56
45	21,760.07	1,595.83	46.68	53.28	1.00	72.88	19.57
46	3,956.36	352.20	34.22	80.28	0.00	98.34	9.77
47	12,010.09	987.86	46.61	73.15	1.00	93.74	29.63
48	2,053.08	209.84	51.22	78.61	1.00	99.64	10.89
49	11,446.79	1,139.30	30.01	73.26	1.00	89.59	28.96
50	3,414.98	342.18	29.43	61.12	0.00	98.98	16.25
51	7,390.33	790.46	32.69	77.88	0.00	97.53	18.83
52	13,672.12	1,914.19	22.34	88.52	1.00	98.51	32.29
53	2,059.12	236.10	25.22	69.57	0.00	97.43	30.03
54	1,536.87	149.77	29.04	85.23	0.00	94.58	55.55
55	52,399.34	3,941.72	44.60	67.78	1.00	83.96	32.52

56	3,371.18	687.20	17.88	70.77	1.00	98.56	40.44
57	3,998.07	738.99	22.01	88.37	1.00	99.35	36.75
58	1,978.13	261.98	37.66	77.14	1.00	84.80	44.34
59	2,651.14	188.79	17.02	73.35	0.00	76.77	18.24
60	19,846.87	1,760.14	37.67	70.89	1.00	90.78	20.64
61	2,434.72	488.34	18.80	85.78	1.00	98.88	65.14
62	17,088.10	1,454.38	41.23	50.54	0.00	71.00	5.49
63	8,709.02	672.74	35.32	52.56	0.00	73.69	30.67
64	1,739.47	184.70	44.25	90.99	1.00	99.37	12.45
65	1,917.79	167.68	42.78	67.95	0.00	68.24	15.17
66	15,027.98	462.78	24.53	51.63	1.00	75.55	13.56
67	1,076.47	70.19	2.15	54.96	0.00	93.62	19.61
68	9,543.22	458.41	51.99	70.53	0.00	77.53	16.37
69	24,329.02	1,809.23	52.59	57.92	1.00	63.90	20.09
70	7,211.12	940.76	22.96	74.21	1.00	90.72	37.96
71	4,419.52	390.84	38.78	61.74	0.00	97.46	27.57
72	1,866.44	54.00	27.23	55.84	0.00	36.66	4.68
73	6,677.59	149.49	43.54	40.20	0.00	64.00	5.65
74	7,667.96	529.38	61.28	68.69	0.00	91.64	7.09
75	45,357.34	951.03	48.82	20.23	1.00	40.70	6.38
76	11,508.60	279.35	38.10	56.73	1.00	38.59	45.97
77	17,935.25	299.60	70.92	40.64	1.00	5.66	0.00
78	12,195.37	199.61	76.56	46.39	0.00	31.98	8.93
79	2,272.99	207.38	57.82	47.03	1.00	92.14	1.94
80	1,429.25	160.31	48.29	62.84	1.00	89.33	8.00
81	13,212.21	975.63	63.96	25.46	1.00	59.66	0.00
82	7,544.43	157.97	71.36	48.10	0.00	14.93	0.00
83	3,030.10	278.78	55.36	40.66	1.00	73.79	1.94
84	40,843.24	756.17	60.08	48.61	1.00	14.61	0.00
85	1,173.65	363.60	4.82	98.09	0.00	100.00	21.15
86	5,413.94	591.58	48.04	63.78	1.00	92.12	18.77
87	14,113.28	237.95	69.61	44.18	0.00	18.84	16.32
88	685.47	233.23	0.25	97.01	0.00	100.00	33.93
89	5,877.14	88.17	84.37	71.82	0.00	21.35	0.00
90	38,686.28	712.85	61.63	33.09	1.00	35.47	20.04
91	14,267.90	354.13	48.47	43.86	0.00	11.04	0.00
92	29,497.80	564.79	56.82	46.56	1.00	36.23	14.24
93	14,548.25	321.54	44.51	42.05	0.00	58.64	23.29
94	19,371.55	513.10	32.41	35.59	1.00	36.11	53.45
95	935.05	61.01	2.28	51.80	0.00	59.60	39.50
96	1,658.51	220.89	35.27	78.61	1.00	99.92	10.87
97	3,456.32	128.83	17.24	76.68	0.00	77.98	40.32
98	4,072.63	88.47	46.06	80.40	0.00	75.10	11.13
99	2,687.28	276.76	50.09	53.92	0.00	88.83	0.00
100	14,830.31	308.71	49.33	33.87	1.00	30.99	26.91
101	5,567.67	456.14	50.61	49.24	0.00	76.57	9.16
102	7,413.75	141.34	57.26	57.57	0.00	63.45	2.73
103	9,323.69	218.77	40.18	31.01	0.00	54.94	20.95
104	7,378.18	118.24	75.53	73.71	0.00	79.91	1.74
105	3,030.09	91.27	22.95	60.57	0.00	5.09	21.89
106	2,228.52	259.57	45.12	48.73	1.00	99.56	9.68
107	8,808.34	811.21	49.86	46.89	1.00	55.23	1.43
108	3,168.45	330.77	49.94	31.78	1.00	99.66	22.11
109	8,195.85	157.89	56.30	66.67	1.00	75.25	10.29
110	5,694.94	142.96	35.18	53.40	0.00	63.39	41.74
111	1,375.02	240.71	25.47	66.98	1.00	98.42	39.80
112	40,962.26	863.66	48.13	39.74	1.00	3.46	34.32
113	9,791.22	180.72	60.36	65.34	0.00	64.66	21.27
114	3,808.82	568.68	31.38	51.20	0.00	78.32	29.61
115	1,257.98	299.30	14.23	80.52	1.00	99.24	45.66
116	1,176.70	202.86	27.04	64.17	1.00	96.52	5.10

117	1,584.42	204.37	31.48	63.66	0.00	93.91	0.00
118	4,251.94	306.06	52.01	66.17	0.00	78.06	22.76
119	7,273.13	728.39	51.44	17.62	1.00	49.07	0.88
120	2,956.45	502.07	25.69	43.42	0.00	68.62	29.58
121	1,128.09	289.97	11.39	76.65	1.00	98.83	41.89
122	1,052.36	214.43	13.81	83.54	0.00	99.37	18.09
123	35,744.55	702.14	61.91	55.55	1.00	75.90	13.19
124	900.37	226.31	7.96	61.87	0.00	86.83	39.94
125	10,536.70	202.03	63.46	56.56	1.00	73.81	7.26
126	8,778.80	172.51	54.42	74.26	1.00	93.29	28.97
127	1,757.54	246.98	27.27	66.45	1.00	99.31	7.92
128	38,562.43	798.29	49.82	57.51	1.00	62.00	2.56
129	6,210.22	224.39	58.88	66.93	1.00	79.61	21.88
130	2,704.26	456.94	14.20	80.39	0.00	95.91	20.10
131	36,653.72	791.06	54.72	52.94	1.00	48.61	37.11
132	10,108.69	223.62	49.71	52.17	1.00	85.04	3.21
133	936.44	144.14	19.22	76.87	1.00	96.24	42.56
134	743.99	196.21	0.62	90.80	0.00	100.00	19.95
135	544,912.36	12,051.43	46.19	52.99	1.00	37.09	57.66
136	10,234.71	703.79	50.32	41.14	1.00	91.58	2.50
137	7,993.65	158.79	53.43	46.92	0.00	82.24	13.19
138	7,207.69	145.31	52.12	45.14	1.00	77.78	10.39
139	2,003.50	245.34	28.80	55.35	1.00	88.02	44.22
140	7,071.01	666.67	50.16	50.41	1.00	57.84	7.82
141	17,868.67	423.23	54.73	41.24	1.00	78.00	15.94
142	12,910.98	1,075.32	56.05	31.41	1.00	27.31	56.27
143	918.95	202.11	6.60	79.67	0.00	96.01	43.15
144	5,842.28	582.07	36.79	42.92	1.00	85.32	32.30
145	9,221.34	580.38	66.65	35.21	1.00	56.41	0.00
146	8,565.89	721.27	46.13	51.08	1.00	61.95	18.98
147	2,513.82	365.38	35.56	41.13	1.00	67.07	51.36
148	3,049.14	161.46	67.23	52.13	1.00	92.47	7.68
149	4,768.51	92.67	55.37	52.48	0.00	84.22	0.00
150	1,529.04	253.12	16.03	71.19	0.00	96.42	30.74
151	9,442.61	681.78	62.11	50.19	1.00	67.30	15.24
152	3,773.16	311.08	60.14	46.58	0.00	75.33	19.13
153	28,328.64	595.51	50.56	42.99	1.00	42.88	33.63
154	2,943.40	327.66	32.90	40.87	1.00	84.82	3.15
155	8,786.23	174.12	56.60	48.05	0.00	85.12	7.62
156	3,871.26	523.16	35.91	41.74	0.00	35.30	26.30
157	3,948.97	345.83	57.98	43.59	1.00	49.30	12.03
158	1,120.37	174.88	18.61	56.40	1.00	75.11	24.78
159	6,952.85	477.41	65.20	39.76	1.00	57.92	14.58
160	3,239.46	74.37	46.95	47.15	0.00	80.27	12.22
161	6,115.44	430.90	62.33	47.66	0.00	87.14	11.56
162	7,169.78	154.92	47.08	64.08	0.00	90.68	3.70
163	3,187.05	300.20	54.06	43.77	0.00	73.86	29.85
164	4,124.63	279.30	52.84	26.48	1.00	51.01	8.58
165	4,036.48	97.97	38.64	61.61	1.00	87.00	13.47
166	13,101.44	398.23	29.23	64.71	1.00	87.73	38.52
167	12,666.89	999.98	49.01	59.35	1.00	72.47	48.15
168	3,918.45	304.39	49.71	75.95	0.00	88.70	21.66
169	31,062.39	3,101.37	48.25	42.47	1.00	38.96	33.78
170	10,968.44	321.47	32.40	47.99	1.00	93.50	33.45
171	5,518.75	99.40	63.07	74.42	1.00	99.21	5.64
172	1,918.93	335.16	26.97	79.08	0.00	78.22	33.81
173	2,938.04	267.79	54.38	51.80	0.00	79.00	28.00
174	4,131.20	574.19	17.95	39.28	1.00	69.42	52.67
175	148,697.98	3,190.79	48.50	40.24	1.00	35.57	33.59
176	20,263.53	426.48	48.95	61.16	1.00	93.87	31.30
177	11,157.47	774.96	67.98	32.94	1.00	77.81	4.04

178	4,614.86	424.89	51.08	42.33	0.00	59.14	10.66
179	8,043.55	205.75	33.65	48.60	1.00	58.63	14.31
180	23,943.95	2,428.85	47.32	44.66	0.00	44.02	36.69
181	5,143.02	108.84	47.91	71.12	1.00	88.11	9.57
182	8,861.46	823.67	49.14	46.62	1.00	66.45	11.54
183	2,524.89	278.65	47.26	49.33	0.00	63.57	20.25
184	1,073.69	187.91	7.76	55.09	0.00	85.55	40.25
185	24,127.69	478.18	55.60	51.76	1.00	93.36	32.20
186	7,106.23	153.15	48.23	66.77	1.00	95.75	19.19
187	13,811.36	294.60	48.02	65.55	1.00	97.32	15.75
188	10,073.49	659.20	57.87	15.69	1.00	38.33	40.78
189	10,710.03	218.78	50.99	73.13	1.00	96.82	23.72
190	2,329.17	65.30	28.32	76.75	1.00	95.88	30.49
191	14,743.44	303.29	53.36	60.16	1.00	89.17	19.78
192	5,877.33	267.74	49.95	65.13	1.00	96.11	12.95
193	131,939.42	2,873.97	47.45	49.39	1.00	86.52	24.61
194	3,480.98	96.74	28.06	79.15	1.00	91.71	42.93
195	7,091.17	414.74	61.14	36.01	0.00	92.39	2.90
196	32,150.24	729.97	42.28	37.79	1.00	97.70	3.53
197	3,451.74	77.75	43.23	77.35	1.00	63.64	16.57
198	4,491.68	497.32	44.48	40.36	0.00	82.46	17.08
199	5,799.62	127.60	44.61	59.88	1.00	83.24	12.39
200	8,450.54	610.61	62.63	52.97	1.00	80.52	30.42
201	30,934.40	2,331.16	62.37	24.55	1.00	86.44	9.10
202	47,052.09	3,268.60	65.51	35.24	1.00	51.11	33.16
203	8,332.14	160.81	65.03	53.75	1.00	98.96	17.58
204	2,271.11	386.59	24.58	44.45	0.00	65.58	7.85
205	6,181.74	773.25	34.65	39.13	0.00	81.11	24.73
206	11,193.62	259.25	41.39	71.37	1.00	96.38	42.30
207	40,178.76	3,644.45	52.07	25.80	1.00	34.54	51.75
208	5,650.26	571.80	49.65	47.91	0.00	82.51	14.00
209	26,327.26	1,985.45	63.72	42.21	1.00	66.70	9.45
210	23,495.02	1,368.53	62.00	20.03	0.00	25.05	24.78
211	6,906.97	673.88	36.18	45.16	0.00	74.21	31.83
212	20,391.78	486.17	38.59	75.82	1.00	99.26	26.55
213	16,953.75	1,261.22	49.40	43.27	0.00	60.67	18.48
214	5,830.68	118.13	51.68	78.28	1.00	99.13	17.42
215	8,324.81	180.59	50.68	66.96	1.00	94.98	20.76
216	11,564.61	638.17	62.32	26.38	0.00	59.23	2.32
217	18,800.99	1,593.70	54.93	44.05	1.00	25.98	11.31
218	9,003.28	689.23	57.22	79.19	1.00	95.62	1.50
219	8,276.61	520.37	69.33	64.63	1.00	70.59	22.71
220	20,599.53	1,575.95	59.88	42.32	1.00	55.06	20.82
221	8,013.48	847.04	41.70	44.38	0.00	73.96	46.57
222	17,545.84	1,269.11	62.46	50.01	1.00	57.75	19.16
223	5,834.94	133.50	41.75	80.88	1.00	98.82	25.66
224	43,145.63	1,067.21	40.88	67.53	1.00	99.29	22.86
225	14,259.39	386.42	39.84	73.85	1.00	95.31	25.07
226	5,229.81	527.10	48.93	53.51	1.00	72.82	7.64
227	8,196.62	926.71	45.56	77.44	1.00	94.77	21.03
228	3,952.87	74.99	57.67	79.47	1.00	89.99	4.46
229	223,325.76	20,115.85	53.18	41.30	1.00	52.36	51.74
230	4,975.51	603.85	42.09	84.55	1.00	97.12	24.24
231	5,480.44	374.10	68.63	46.33	0.00	79.88	28.99
232	4,450.42	313.36	64.28	60.98	0.00	64.59	11.95
233	17,973.92	1,068.27	60.88	39.19	0.00	51.29	23.11
234	5,735.12	670.41	35.41	91.20	1.00	98.81	21.07
235	15,207.32	358.71	42.78	81.08	1.00	96.22	47.37
236	17,981.59	464.22	43.04	67.69	1.00	99.18	29.84
237	3,037.44	338.43	31.23	73.60	1.00	97.84	19.95
238	25,930.60	2,365.07	53.56	35.55	1.00	64.91	12.27

239	50,884.88	4,132.68	57.77	45.98	1.00	53.82	21.44
240	6,851.87	217.03	38.86	83.15	1.00	98.80	35.87
241	4,937.96	525.23	46.57	53.90	0.00	75.45	17.91
242	6,411.40	561.66	54.77	52.90	1.00	80.79	2.96
243	21,992.28	2,038.36	50.23	52.31	1.00	87.04	14.44
244	66,388.67	5,366.56	57.01	33.21	1.00	69.57	24.54
245	3,701.91	333.40	39.54	35.29	1.00	80.06	4.47
246	14,135.97	1,174.63	55.49	43.91	0.00	88.92	20.08
247	16,651.66	1,593.61	48.55	47.16	1.00	90.47	35.04
248	2,186.84	212.91	48.09	73.15	0.00	81.14	2.65
249	131,559.86	13,029.12	49.67	41.71	1.00	72.25	54.14
250	5,455.89	132.93	57.76	76.27	1.00	92.80	4.21
251	3,968.31	286.29	63.92	48.84	0.00	83.64	12.81
252	6,465.86	768.49	42.03	44.59	1.00	64.97	8.60
253	9,841.72	253.26	38.03	82.01	1.00	90.48	36.36
254	47,951.73	3,888.37	56.52	39.97	1.00	67.19	19.28
255	6,305.59	548.00	56.97	68.58	1.00	94.39	13.87
256	5,714.78	771.53	36.96	45.58	0.00	75.12	28.66
257	5,553.73	473.51	59.12	59.52	0.00	82.85	20.50
258	3,661.66	358.08	51.86	79.25	1.00	97.31	13.79
259	3,336.15	338.74	48.66	86.12	1.00	93.04	4.54
260	21,950.52	1,519.88	49.18	23.18	1.00	41.30	17.20
261	2,078.16	374.21	22.76	63.50	0.00	76.80	12.70
262	5,959.05	475.47	59.34	46.79	0.00	91.43	17.44
263	182,450.67	22,617.38	37.77	34.83	1.00	39.42	41.33
264	8,654.18	641.04	65.23	41.20	0.00	79.52	15.61
265	6,744.04	1,085.25	29.62	78.15	1.00	97.93	45.74
266	3,174.02	539.48	27.16	86.99	1.00	95.20	48.41
267	3,123.04	369.51	39.20	45.60	0.00	76.07	17.11
268	2,744.17	315.06	41.63	73.46	1.00	94.43	0.00
269	8,656.15	567.08	63.52	41.50	0.00	93.27	6.54
270	7,461.19	493.83	54.69	31.43	0.00	80.67	19.18
271	4,643.77	913.37	20.92	52.91	0.00	80.17	42.61
272	34,480.01	2,815.41	55.14	27.70	0.00	86.43	18.50
273	20,690.25	1,765.60	54.47	27.95	0.00	81.45	27.37
274	5,360.82	980.17	22.40	83.22	1.00	92.41	34.56
275	7,301.00	553.04	61.95	43.19	0.00	89.07	9.48
276	11,575.11	983.64	57.05	51.63	0.00	87.50	7.73
277	21,370.36	2,800.73	35.99	53.69	1.00	85.07	33.25
278	98,215.91	10,158.56	48.30	43.18	1.00	78.09	25.25
279	137,212.01	17,911.38	35.32	44.15	1.00	31.66	42.23
280	17,125.30	1,206.96	51.63	28.16	0.00	73.09	11.27
281	6,179.20	428.19	56.15	38.89	0.00	89.57	0.00
282	8,091.19	1,561.54	18.00	76.61	1.00	93.27	31.00
283	3,259.31	582.58	24.08	59.55	0.00	84.70	10.44
284	10,942.19	2,025.78	19.96	72.56	1.00	82.57	21.88
285	66,764.57	9,386.90	31.95	41.69	1.00	43.25	36.42
286	2,856.20	789.85	6.58	86.31	1.00	85.52	48.91
287	21,597.27	2,460.18	41.50	68.46	1.00	76.98	30.37
288	11,051.77	1,207.21	43.50	56.35	1.00	89.80	0.00
289	4,391.80	543.01	38.21	47.02	0.00	68.47	35.24
290	15,077.08	877.54	61.35	29.73	0.00	65.46	10.02
291	44,877.99	5,204.64	43.06	38.58	1.00	86.39	2.32
292	10,243.26	1,722.97	27.78	50.19	1.00	75.70	27.39
293	3,986.70	381.28	45.15	52.31	0.00	92.19	21.92
294	20,644.32	1,285.57	58.08	30.74	0.00	66.27	7.48
295	37,660.47	5,928.97	26.25	55.62	1.00	83.94	32.37
296	21,458.41	3,283.83	27.86	68.36	1.00	95.28	0.00
297	4,717.66	1,252.54	8.58	89.14	1.00	91.81	33.99
298	10,699.68	858.89	59.17	48.04	0.00	96.17	13.92
299	991.09	372.59	2.78	90.01	1.00	89.33	48.51

300	4,790.14	849.39	24.41	67.78	1.00	72.64	30.01
301	31,917.10	3,587.93	44.60	51.84	1.00	85.42	25.46
302	8,791.36	647.01	52.00	27.57	0.00	74.69	12.98
303	15,062.19	2,591.34	23.45	68.73	1.00	91.57	12.21
304	45,668.28	5,169.55	41.08	54.95	1.00	78.19	31.24
305	5,828.03	1,180.34	19.25	62.40	1.00	96.96	16.75
306	39,396.66	4,420.08	41.35	55.03	1.00	79.00	21.90
307	1,660.29	262.85	25.12	69.70	0.00	76.63	25.93
308	14,923.08	1,559.29	46.76	30.84	0.00	85.04	14.76
309	39,877.79	2,358.76	60.94	29.91	1.00	58.65	0.00
310	5,149.28	691.31	35.78	40.81	0.00	98.46	19.82
311	23,008.59	2,681.06	42.99	50.62	1.00	89.19	6.92
312	28,067.43	3,352.87	37.26	72.99	1.00	97.26	30.88
313	5,291.71	542.74	47.26	64.62	1.00	89.56	10.32
314	1,807.91	300.37	20.45	72.84	0.00	95.46	28.64
315	75,235.51	9,055.95	35.42	44.39	1.00	56.88	38.40
316	5,887.30	795.50	33.38	83.51	1.00	99.13	31.53
317	3,641.33	222.31	59.31	36.92	0.00	82.67	0.00
318	2,930.19	514.65	20.88	49.44	0.00	70.12	31.46
319	5,638.01	506.86	46.89	65.72	1.00	86.85	2.97
320	61,329.94	6,962.87	38.07	51.75	1.00	72.13	18.18
321	1,963.81	332.48	21.77	80.21	1.00	91.37	29.71
322	5,274.55	582.10	44.84	63.85	0.00	96.12	3.19
323	11,407.55	1,575.09	30.67	75.00	1.00	99.80	26.64
324	4,666.40	717.86	26.18	82.56	1.00	94.41	40.32
325	8,656.77	779.26	47.30	57.68	0.00	96.41	26.50
326	7,680.85	809.48	45.97	68.91	0.00	94.23	32.99
327	6,950.72	846.78	38.45	79.41	1.00	91.79	7.71
328	4,879.06	634.88	36.81	59.02	0.00	77.33	29.80
329	13,024.82	1,811.66	34.86	41.31	1.00	87.64	14.61
330	5,882.21	759.39	35.72	46.68	0.00	74.28	7.84
331	4,496.45	658.84	30.01	48.67	0.00	84.67	13.16
332	5,680.11	771.74	32.68	87.45	1.00	97.98	5.00
333	5,188.36	559.18	40.25	72.10	1.00	99.48	21.88
334	2,957.58	257.27	53.06	80.29	1.00	99.19	1.69
335	1,702.91	302.89	21.57	67.26	1.00	80.80	41.88
336	3,022.38	335.44	26.11	68.21	0.00	88.41	10.09
337	2,960.17	427.19	32.65	84.17	1.00	98.52	36.96
338	5,436.96	443.63	45.31	20.15	0.00	65.22	17.18
339	3,327.37	317.60	40.73	52.62	0.00	78.34	23.32
340	4,871.61	778.21	23.47	68.61	1.00	93.63	35.75
341	7,717.39	964.04	24.10	58.61	0.00	77.95	46.57
342	12,482.74	1,686.54	30.86	83.77	1.00	97.87	31.05
343	10,828.96	1,259.79	42.18	51.34	0.00	92.60	18.22
344	23,761.77	1,446.36	59.59	36.16	0.00	59.40	19.40
345	5,046.53	666.21	36.69	53.49	1.00	85.78	23.98
346	3,876.49	190.16	70.38	49.08	0.00	87.06	0.00
347	7,114.88	563.06	53.04	67.93	1.00	89.37	0.00
348	4,055.93	525.83	24.82	63.63	0.00	78.38	44.03
349	5,445.76	831.21	25.26	63.72	1.00	89.78	15.07
350	3,832.78	488.04	32.23	80.69	1.00	86.65	23.78
351	6,488.75	686.18	34.01	31.57	0.00	77.33	33.43
352	8,369.39	1,059.79	28.30	61.96	1.00	84.35	41.88
353	3,629.16	302.92	45.05	49.88	0.00	90.18	32.23
354	7,949.04	898.85	31.25	47.80	0.00	79.76	30.67
355	3,152.15	328.31	29.20	55.69	1.00	74.36	11.86
356	3,215.89	375.38	36.67	85.87	1.00	92.74	28.91
357	3,865.01	492.83	26.60	56.55	1.00	76.71	40.77
358	5,211.02	545.64	33.20	53.18	0.00	63.14	41.52
359	18,200.96	1,679.96	40.71	42.81	0.00	75.38	20.72
360	15,369.83	1,388.08	38.53	48.27	1.00	60.48	17.59

361	7,640.74	512.83	55.93	31.68	0.00	82.69	3.35
362	5,095.74	462.43	41.15	47.59	0.00	72.49	32.49
363	63,263.59	3,864.71	59.49	28.02	0.00	65.66	18.73
364	3,210.96	241.90	46.91	78.61	1.00	74.96	19.48
365	2,793.68	210.38	48.88	77.14	1.00	95.40	4.90
366	45,519.74	4,183.12	39.73	52.86	1.00	73.06	33.77
367	55,417.03	4,916.24	41.60	37.23	1.00	70.04	37.71
368	4,565.82	365.64	43.71	67.74	1.00	93.73	29.70
369	7,891.24	732.01	36.62	51.01	1.00	75.66	34.18
370	27,623.62	2,280.75	42.81	47.52	1.00	68.84	23.42
371	30,453.50	2,540.28	42.45	42.32	1.00	66.19	11.57
372	3,527.96	396.80	29.70	61.77	1.00	83.16	29.02
373	13,746.90	1,117.52	43.27	50.77	1.00	72.21	16.72
374	3,671.97	449.33	24.03	56.28	1.00	60.59	17.87
375	4,884.16	473.49	33.01	62.32	1.00	70.89	8.20
376	2,456.86	350.22	14.84	72.23	1.00	32.15	36.09
377	3,564.01	646.47	10.07	48.23	1.00	32.91	67.62
378	1,977.26	257.27	22.25	64.08	1.00	42.07	16.58
379	2,209.30	412.13	8.01	55.03	1.00	34.55	74.56
380	20,135.98	453.32	43.27	15.08	1.00	54.98	31.26
381	6,429.87	143.64	44.03	67.31	1.00	93.38	17.30
382	10,683.72	250.80	40.10	67.91	1.00	73.04	37.70
383	20,282.57	506.35	35.72	50.04	1.00	80.97	29.51
384	12,896.37	330.22	33.96	55.86	1.00	78.00	22.69
385	9,167.09	865.78	14.72	59.72	1.00	99.07	46.40
386	4,490.40	262.39	45.99	80.30	0.00	98.80	25.37
387	16,373.99	1,226.36	31.86	66.39	0.00	97.84	25.51
388	2,635.35	83.92	39.25	78.13	0.00	97.38	8.03
389	128,664.83	9,197.62	38.20	38.90	1.00	87.28	64.27
390	2,844.38	222.15	32.89	69.52	1.00	94.47	3.98
391	2,308.12	135.14	55.08	54.30	0.00	98.42	9.82
392	7,517.44	485.08	40.51	51.38	1.00	98.09	29.12
393	7,145.25	258.61	40.60	67.01	0.00	97.10	22.85
394	7,981.84	450.77	44.22	46.84	0.00	96.74	17.62
395	209,820.07	14,989.48	37.50	61.59	1.00	92.97	56.36
396	15,889.60	1,211.49	36.34	59.94	1.00	98.92	40.41
397	241,900.47	17,577.06	36.65	51.09	1.00	92.28	67.77
398	5,807.59	421.55	38.42	68.61	1.00	99.74	11.53
399	11,531.82	841.58	37.98	66.38	1.00	99.53	29.34
400	290,891.39	21,615.34	35.31	46.13	1.00	79.52	72.16
401	4,053.11	352.90	27.60	45.98	1.00	93.74	31.93
402	14,461.95	1,176.46	31.13	38.44	1.00	95.21	39.83
403	9,824.63	797.83	31.25	53.48	1.00	96.73	47.07
404	6,745.28	670.44	19.69	70.47	0.00	98.50	41.11
405	16,588.74	1,337.01	31.82	36.08	0.00	69.34	38.27
406	2,203.36	81.14	29.38	86.50	0.00	99.96	25.22
407	13,109.05	1,106.47	31.69	61.29	1.00	95.73	44.64
408	365,269.21	28,008.31	33.82	57.21	1.00	60.40	58.69
409	9,300.03	1,529.19	4.19	56.44	1.00	83.42	53.27
410	43,677.53	4,610.22	16.90	47.87	1.00	72.51	69.79
411	62,886.74	7,016.27	14.90	50.05	1.00	80.07	55.06
412	11,831.96	781.33	49.84	37.42	1.00	81.65	41.19
413	3,104.42	461.11	6.58	56.12	0.00	87.75	32.89
414	7,017.24	696.50	20.06	54.73	0.00	94.69	22.53
415	6,448.46	605.11	24.87	47.62	0.00	74.59	12.68
416	5,499.66	492.48	24.87	41.19	1.00	98.39	14.35
417	6,181.93	683.37	17.02	65.00	1.00	93.39	41.23
418	7,133.26	701.73	22.73	52.34	1.00	65.85	16.53
419	21,171.52	2,924.78	8.12	64.46	1.00	85.16	67.05
420	4,564.67	551.62	13.08	69.94	1.00	76.36	8.53
421	2,169.68	196.83	21.12	35.20	1.00	49.06	4.91

422	17,237.76	1,792.09	18.59	52.60	1.00	89.48	42.99
423	3,869.88	349.25	23.65	46.31	1.00	78.54	23.66
424	9,518.61	691.88	39.11	42.86	1.00	17.96	21.00
425	7,085.10	301.39	18.70	66.29	0.00	83.24	12.69
426	21,610.90	1,983.76	23.56	37.62	1.00	59.19	27.43
427	70,095.70	8,230.82	12.95	33.72	1.00	68.50	52.81
428	1,653.80	301.00	0.44	66.35	1.00	90.21	44.09
429	15,723.84	2,070.61	9.67	41.68	1.00	78.06	72.22
430	1,739.54	135.47	11.96	47.26	0.00	88.46	28.86
431	14,572.75	867.97	61.25	37.85	1.00	19.55	0.00
432	1,720.76	323.94	0.39	59.37	0.00	63.49	27.88
433	4,044.29	620.22	6.04	62.20	1.00	80.52	44.11
434	3,243.78	214.51	24.48	46.62	1.00	80.49	15.95
435	10,595.41	1,015.46	21.41	42.92	1.00	66.33	28.95
436	6,658.90	695.27	18.48	38.36	1.00	57.07	31.81
437	9,196.94	775.76	27.12	23.47	1.00	32.34	30.52
438	2,609.15	168.79	51.25	44.66	0.00	66.58	0.00
439	7,122.32	1,159.30	4.85	61.22	1.00	81.28	56.01
440	2,504.87	220.78	28.41	28.12	1.00	50.10	33.72
441	3,945.63	299.37	36.48	50.41	1.00	80.75	17.59
442	1,272.52	92.08	42.59	55.28	0.00	89.69	22.88
443	19,930.78	1,922.25	22.72	43.92	1.00	71.02	33.99
444	3,351.46	460.89	9.53	43.55	0.00	55.34	14.27
445	8,833.76	776.94	28.81	39.16	1.00	53.12	37.17
446	4,453.22	321.92	42.29	47.08	1.00	88.75	26.67
447	4,243.89	412.89	21.75	29.88	1.00	49.08	27.91
448	4,416.90	523.62	13.15	34.80	1.00	43.10	23.27
449	7,152.26	701.43	22.76	33.25	1.00	51.65	33.17
450	4,425.41	371.98	31.82	59.76	1.00	65.99	1.96
451	5,934.16	447.65	38.65	34.87	1.00	82.72	11.12
452	4,280.84	307.51	36.18	53.88	0.00	84.12	17.44
453	11,622.24	817.40	42.39	30.07	1.00	79.94	10.57
454	17,052.33	1,235.08	40.51	28.48	1.00	86.43	46.94
455	2,651.64	246.86	20.77	46.02	1.00	82.45	35.82
456	1,237.69	115.94	18.06	62.28	1.00	79.32	29.91
457	9,501.75	589.86	40.14	48.44	0.00	90.28	12.60
458	15,754.50	1,075.45	39.39	39.04	1.00	91.03	15.04
459	4,914.85	385.20	31.57	31.49	1.00	87.72	17.68
460	3,363.43	232.17	43.43	59.62	0.00	87.44	10.33
461	2,688.80	179.10	46.44	47.89	1.00	93.83	4.31
462	8,413.13	414.48	48.53	38.79	0.00	94.49	6.25
463	4,652.91	382.61	20.82	53.77	1.00	80.59	2.31
464	4,488.01	467.36	13.61	89.84	0.00	93.33	26.73
465	2,959.16	239.69	28.79	57.62	1.00	92.13	22.20
466	6,655.86	510.31	34.32	40.54	1.00	84.93	6.55
467	2,539.94	94.54	46.90	59.34	0.00	89.57	0.00
468	6,542.73	686.92	15.62	73.80	1.00	89.34	32.59
469	950.84	82.53	29.58	80.83	0.00	89.52	0.00
470	5,268.57	329.21	55.98	34.76	1.00	47.92	0.00
471	4,168.40	217.51	39.39	56.40	0.00	94.75	3.69
472	7,188.31	664.12	22.17	40.24	1.00	45.48	11.72
473	6,341.10	480.15	36.50	48.54	1.00	84.94	2.45
474	16,183.84	1,238.50	34.67	42.00	1.00	45.68	27.26
475	15,491.42	1,321.62	27.78	49.46	1.00	71.50	23.93
476	4,946.20	357.30	40.63	47.67	1.00	71.24	1.55
477	2,143.33	153.74	42.48	69.86	1.00	39.92	0.00
478	2,921.74	213.51	39.74	49.80	1.00	34.11	2.24
479	1,267.36	110.57	28.69	80.13	1.00	83.64	40.72
480	28,508.64	612.48	46.64	35.78	1.00	35.36	8.78
481	36,237.56	2,252.94	49.79	49.45	0.00	97.43	3.69
482	129,029.36	9,220.90	38.23	45.27	1.00	93.57	40.93

483	6,231.12	387.83	43.29	52.34	0.00	88.44	0.00
484	7,214.91	256.28	17.57	67.93	0.00	92.82	41.11
485	6,748.61	146.79	45.91	70.33	0.00	52.09	1.94
486	4,294.00	145.02	25.00	55.32	0.00	66.29	33.65
487	4,414.12	239.99	71.13	67.24	0.00	96.26	0.00
488	3,575.25	212.27	55.81	53.52	0.00	93.31	1.30
489	14,545.51	876.72	50.76	62.25	0.00	93.96	0.00
490	8,014.14	270.19	30.12	75.10	0.00	25.26	9.04
491	7,528.28	275.01	16.55	51.87	1.00	79.52	21.10
492	7,949.76	183.98	40.74	62.42	0.00	88.23	10.57
493	23,563.62	1,818.19	19.43	54.59	1.00	7.73	24.83
494	2,902.64	255.94	18.87	47.78	0.00	83.51	38.29
495	12,725.46	1,503.46	11.90	44.71	0.00	28.66	24.51
496	6,264.93	233.28	15.54	52.29	1.00	70.46	17.33
497	6,566.80	469.79	39.00	32.25	0.00	90.67	3.43
498	43,101.62	3,077.71	37.16	34.27	1.00	91.32	23.17
499	13,175.52	1,010.60	33.03	45.66	1.00	85.98	10.57
500	9,422.11	725.64	33.71	38.48	0.00	76.75	15.50
501	3,217.34	236.85	36.10	69.24	0.00	97.84	27.59
502	38,007.13	2,739.70	36.32	35.84	1.00	94.66	15.41
503	12,054.65	833.17	38.39	57.20	1.00	4.59	0.69
504	8,522.62	461.62	44.97	60.56	0.00	4.29	0.00
505	27,362.32	2,156.26	21.53	42.55	1.00	11.08	26.57
506	2,601.20	389.91	2.71	53.20	0.00	78.17	50.25
507	2,996.16	176.49	66.45	76.01	0.00	0.00	0.00
508	36,885.55	757.11	50.77	43.02	0.00	25.61	9.44
509	9,087.68	357.14	54.69	55.68	0.00	10.10	6.63
510	65,500.76	1,385.31	48.10	42.74	1.00	21.70	21.94
511	8,378.85	628.02	34.83	38.24	0.00	84.16	19.08
512	11,021.33	214.28	56.43	56.41	0.00	51.47	9.97
513	18,310.14	1,284.89	36.96	50.41	1.00	86.91	2.47
514	2,026.08	185.91	18.75	42.47	0.00	89.38	13.75
515	2,781.37	150.67	69.23	53.23	0.00	96.12	0.00
516	4,783.26	467.51	26.29	41.65	0.00	80.55	36.29
517	7,880.83	223.80	27.41	55.18	1.00	83.91	27.43
518	9,427.79	638.33	53.43	57.36	0.00	3.81	1.62
519	8,862.39	197.06	43.82	64.58	0.00	73.69	3.46
520	67,119.71	5,191.38	26.72	37.02	1.00	12.89	27.73
521	4,964.26	296.97	48.56	60.93	0.00	99.04	0.00
522	186,604.05	13,365.38	37.80	46.06	1.00	90.56	29.80
523	3,256.38	323.83	20.55	48.74	0.00	35.22	6.69
524	7,035.11	597.37	33.43	63.02	1.00	5.04	0.00
525	3,877.75	335.61	36.33	50.43	0.00	2.20	9.59
526	12,134.65	878.67	39.01	57.13	1.00	94.36	31.43
527	21,817.18	697.51	22.34	36.70	1.00	47.53	21.23
528	4,316.55	236.05	70.56	67.21	0.00	91.71	0.00
529	9,757.27	311.93	22.46	66.72	1.00	90.81	25.03
530	4,651.69	278.71	56.76	59.71	0.00	92.42	8.45
531	4,739.16	227.30	51.16	69.12	0.00	0.00	2.14
532	7,644.64	174.61	52.20	66.47	0.00	1.09	2.53
533	4,960.98	330.41	59.13	63.36	0.00	1.64	0.00
534	7,717.59	227.36	25.99	49.76	1.00	62.36	8.57
535	13,257.31	969.80	38.13	38.13	0.00	83.29	19.30
536	11,072.01	1,103.71	18.63	49.94	0.00	84.32	24.91
537	2,263.06	213.48	23.19	45.05	0.00	19.90	14.43
538	25,692.65	522.86	53.89	44.46	0.00	53.97	0.00
539	7,879.50	667.09	26.36	37.85	0.00	81.95	20.57
540	32,372.16	686.75	49.41	66.93	0.00	80.26	26.00
541	5,562.39	491.33	28.68	54.40	1.00	7.08	0.00
542	241,870.96	17,572.21	36.69	39.19	1.00	88.22	36.29
543	1,778.76	211.99	6.52	36.44	0.00	36.48	29.00

544	5,478.06	182.89	30.75	43.66	0.00	15.00	13.57
545	12,507.42	305.42	45.99	53.34	0.00	36.73	10.29
546	1,210.23	131.71	12.92	44.13	0.00	22.34	6.44
547	40,324.81	912.21	43.62	52.83	1.00	78.39	11.47
548	2,923.32	85.46	25.03	46.84	0.00	61.61	0.00
549	6,610.66	231.18	18.83	74.14	0.00	87.81	10.37
550	2,592.15	254.24	21.92	48.04	0.00	90.83	41.84
551	3,037.64	245.56	30.26	45.31	0.00	92.64	6.16
552	895.53	41.70	7.79	74.86	0.00	89.34	12.74
553	36,552.84	2,999.05	29.84	46.93	1.00	53.41	40.27
554	17,131.29	1,524.11	24.39	32.97	0.00	23.06	37.25
555	4,209.16	132.17	33.70	54.39	0.00	47.74	21.52
556	2,776.29	163.69	60.26	42.69	0.00	84.99	1.62
557	2,867.31	384.98	9.11	57.25	0.00	80.62	28.12
558	31,092.44	1,224.56	25.02	66.03	1.00	82.48	32.01
559	2,872.96	254.35	28.15	54.67	0.00	4.85	1.66
560	5,366.90	351.73	61.75	51.00	0.00	7.30	0.00
561	290,589.64	21,572.42	35.40	37.78	1.00	81.85	49.02
562	3,693.56	263.81	36.83	46.87	0.00	89.78	3.84
563	11,044.67	999.61	23.85	57.42	0.00	93.66	41.84
564	7,406.19	355.04	8.56	79.12	1.00	94.15	38.87
565	25,009.06	2,153.15	25.94	45.77	1.00	82.54	48.46
566	3,325.04	205.27	55.82	53.81	0.00	93.04	9.19
567	12,899.49	1,218.80	21.94	48.54	1.00	95.68	48.55
568	6,211.68	428.19	38.49	46.24	1.00	8.65	10.08
569	4,787.35	389.79	32.34	54.58	0.00	79.83	18.40
570	9,617.44	288.01	26.47	40.08	1.00	43.32	40.79
571	1,032.39	119.24	0.50	64.06	0.00	46.18	29.29
572	4,850.92	353.76	41.34	64.74	0.00	88.59	13.89
573	2,968.14	267.46	30.99	49.71	0.00	2.42	8.15
574	7,427.30	700.87	24.61	62.75	1.00	84.78	45.49
575	31,072.72	2,941.36	21.51	65.16	1.00	55.32	51.22
576	9,346.66	1,140.10	13.24	47.87	0.00	90.31	52.20
577	4,361.61	166.82	15.95	29.99	1.00	45.62	29.75
578	3,950.00	412.24	21.42	61.13	0.00	28.60	31.01
579	9,839.57	909.88	5.91	87.41	1.00	98.61	40.90
580	40,850.04	4,108.13	18.87	45.96	1.00	80.29	39.71
581	7,476.32	721.50	19.91	42.11	1.00	76.81	1.89
582	2,860.40	230.57	37.76	40.54	1.00	5.10	10.92
583	3,927.81	428.76	18.75	55.51	1.00	72.70	1.95
584	6,532.42	319.90	8.69	73.88	1.00	84.53	54.96
585	1,852.77	142.76	40.54	57.53	0.00	7.56	7.15
586	1,525.28	142.55	24.43	52.46	0.00	80.25	25.88
587	2,529.25	260.40	17.53	54.83	0.00	5.38	15.50
588	4,701.72	499.66	20.77	64.63	0.00	85.27	14.79
589	4,042.77	432.79	17.06	49.72	0.00	57.26	37.81
590	9,236.33	939.89	26.09	56.85	1.00	82.00	30.49
591	5,975.22	584.04	20.64	49.67	0.00	13.67	19.23
592	2,192.30	196.82	25.16	64.90	0.00	94.86	1.89
593	5,592.04	623.35	17.43	76.51	0.00	91.28	28.31
594	1,737.26	159.66	27.57	58.23	0.00	44.14	17.87
595	2,701.33	243.35	24.84	42.52	0.00	28.02	17.79
596	4,844.76	171.49	18.13	35.72	1.00	53.09	33.40
597	1,838.47	181.54	19.44	53.24	0.00	42.70	15.97
598	2,983.95	323.26	22.35	66.07	0.00	93.83	16.40
599	5,773.04	461.88	2.23	90.44	1.00	99.55	27.51
600	4,121.30	506.67	8.89	70.88	1.00	94.09	22.02
601	3,081.73	259.50	42.26	68.56	0.00	95.73	7.82
602	8,169.02	891.30	10.16	88.89	1.00	98.70	47.20
603	2,170.45	247.87	16.78	75.81	1.00	95.71	16.51
604	1,293.80	147.05	19.39	92.72	1.00	99.64	17.30

605	3,828.10	390.69	12.48	59.75	1.00	68.93	29.80
606	5,383.24	719.24	5.24	97.07	1.00	99.40	45.33
607	4,408.79	464.73	10.63	67.33	1.00	85.07	7.62
608	15,886.44	1,565.05	19.90	71.29	0.00	93.48	3.50
609	2,764.78	287.27	21.77	73.72	0.00	87.17	19.91
610	3,212.54	266.44	44.78	69.26	1.00	82.38	1.67
611	8,366.89	662.60	43.43	50.56	1.00	62.06	5.62
612	1,437.81	128.93	18.51	63.60	1.00	70.26	0.00
613	11,299.23	1,356.62	12.59	67.10	0.00	92.72	23.58
614	1,351.75	163.19	7.23	95.14	0.00	98.47	9.88
615	6,179.02	767.11	10.66	91.33	0.00	98.71	11.70
616	33,442.97	3,646.19	15.39	74.78	1.00	91.90	18.06
617	7,399.76	1,054.78	5.49	92.61	0.00	99.70	59.95
618	4,120.79	425.86	21.92	58.47	1.00	72.38	21.27
619	17,797.32	2,187.12	6.35	60.14	1.00	92.17	30.65
620	8,033.45	711.25	29.90	64.40	1.00	81.90	12.30
621	13,213.33	1,671.73	6.36	79.56	0.00	91.70	43.96
622	1,214.34	141.15	6.34	85.98	1.00	85.73	0.00
623	2,643.09	208.93	8.18	58.53	0.00	73.90	21.03
624	2,728.84	247.10	9.67	79.62	1.00	94.01	15.88
625	3,551.08	421.98	11.81	88.84	0.00	93.47	22.82
626	7,222.08	1,287.86	4.43	54.49	0.00	82.20	35.78
627	1,088.84	764.65	7.71	88.14	1.00	97.01	17.33
628	16,998.01	3,711.47	7.30	81.67	1.00	92.92	32.48
629	1,379.61	579.55	6.18	90.04	1.00	100.00	17.18
630	4,529.13	704.30	8.88	82.35	0.00	90.90	54.73
631	4,760.04	579.32	14.06	54.67	1.00	67.97	4.94
632	10,887.11	2,050.62	14.13	46.35	1.00	38.88	14.29
633	12,013.44	2,599.00	7.15	76.15	1.00	88.89	3.01
634	506.39	183.26	18.61	91.02	1.00	100.00	17.08
635	359.33	242.11	14.72	92.38	1.00	100.00	27.74
636	3,172.60	1,031.96	9.29	57.32	1.00	83.04	6.84
637	535.79	170.63	5.35	76.13	1.00	94.81	16.21
638	2,504.00	325.61	13.55	48.91	1.00	74.32	25.89
639	101.74	121.79	4.95	98.64	1.00	99.54	25.55
640	21,607.56	4,573.20	8.72	98.67	1.00	74.53	8.29
641	22,547.54	4,826.38	8.96	81.44	1.00	94.22	36.20
642	19,924.00	4,294.74	7.68	93.20	1.00	93.48	13.40
643	1,797.97	220.05	13.36	69.98	1.00	78.77	0.00
644	1,691.09	282.60	8.29	75.52	1.00	89.39	18.50
645	2,883.09	403.63	9.36	72.83	1.00	95.79	20.01
646	4,386.44	359.79	9.95	64.98	0.00	91.14	22.35
647	8,044.82	765.73	6.95	79.73	0.00	97.77	25.01
648	6,609.34	1,653.12	4.63	93.61	0.00	98.99	56.53
649	620.66	262.91	16.40	94.38	1.00	99.07	70.20
650	808.53	117.17	15.53	79.74	0.00	99.86	4.49
651	1,451.29	275.64	6.66	68.06	1.00	85.58	29.49
652	1,250.11	397.54	12.69	84.30	0.00	99.31	45.96
653	670.67	130.40	7.22	94.19	1.00	99.34	0.00
654	5,022.32	554.96	12.76	69.75	0.00	84.87	29.35
655	10,312.14	810.07	11.11	54.37	0.00	73.56	18.91
656	8,644.13	640.15	10.75	51.29	0.00	66.37	7.42
657	3,205.00	768.80	11.38	75.22	0.00	97.19	46.30
658	469.24	211.65	7.59	88.36	1.00	95.48	54.96
659	2,038.32	533.27	6.71	81.92	1.00	91.64	17.73
660	8,515.33	921.36	8.68	76.36	0.00	97.60	41.53
661	2,506.94	179.42	8.99	79.25	0.00	94.59	27.38
662	2,887.64	624.43	7.12	75.70	1.00	91.39	20.87
663	7,824.50	1,585.97	10.56	70.85	1.00	87.50	29.96
664	13,150.02	1,649.88	9.13	64.17	1.00	92.39	36.03
665	11,313.24	2,406.72	12.18	52.74	1.00	89.71	30.19

666	1,074.36	269.38	16.87	90.21	1.00	98.75	2.12
667	2,686.93	265.97	11.05	80.76	0.00	99.38	10.65
668	339.78	84.42	11.72	89.45	1.00	100.00	48.40
669	15,134.65	1,342.14	9.85	56.67	1.00	88.98	33.96
670	2,275.31	146.90	6.49	65.45	1.00	99.83	26.72
671	3,431.98	413.31	12.06	73.35	1.00	97.92	27.25
672	264.52	108.15	14.18	82.33	1.00	96.72	13.06
673	16,855.12	1,477.82	10.37	74.04	1.00	90.53	38.14
674	3,572.44	430.90	8.82	57.71	1.00	87.01	14.77
675	3,149.15	321.19	11.00	79.72	1.00	99.09	33.77
676	930.42	173.52	21.66	90.28	1.00	99.88	7.28
677	2,482.84	477.57	9.98	86.68	1.00	98.77	29.42
678	2,982.18	267.21	11.40	72.52	1.00	97.69	11.24
679	2,020.54	115.45	16.81	75.67	1.00	98.88	0.00
680	2,001.41	219.02	10.85	86.98	1.00	93.33	31.90
681	5,981.80	534.26	14.30	73.00	1.00	90.29	12.24
682	618.67	158.08	24.71	97.88	1.00	99.10	20.52
683	1,392.60	163.62	15.74	94.73	1.00	98.56	18.08
684	2,483.92	266.24	9.39	78.67	1.00	98.78	13.78
685	2,983.33	550.42	26.07	58.75	1.00	90.23	14.86
686	4,480.39	570.92	6.02	86.66	1.00	95.85	36.38
687	4,167.43	380.93	8.40	95.19	1.00	99.82	15.69
688	2,015.92	476.13	33.93	87.23	1.00	92.35	11.71
689	895.43	170.49	44.16	91.92	1.00	96.35	3.59
690	2,164.74	180.85	16.53	90.10	1.00	100.00	1.58
691	2,918.63	421.27	3.72	78.11	1.00	88.67	41.74
692	2,136.44	171.79	15.61	81.67	1.00	99.64	6.29
693	4,240.93	334.65	11.02	79.37	1.00	99.56	0.00
694	1,480.54	141.69	14.44	81.06	1.00	98.38	24.14
695	72,638.62	8,161.91	5.65	39.39	1.00	56.29	43.61
696	47,498.98	5,973.63	5.47	46.12	1.00	61.88	56.45
697	23,808.67	2,823.35	6.63	42.94	1.00	72.05	51.60
698	5,837.29	1,242.71	19.37	52.85	1.00	77.11	5.10
699	6,046.64	1,098.38	21.04	54.61	0.00	74.56	7.69
700	317,308.05	23,771.69	12.25	58.30	1.00	73.47	57.83
701	12,870.88	231.82	11.25	45.71	1.00	31.14	4.13
702	3,967.29	65.87	6.47	50.29	1.00	14.61	2.31
703	10,057.98	211.03	13.12	57.78	1.00	41.91	1.88
704	5,536.06	103.06	13.08	77.90	1.00	60.74	0.00
705	17,896.93	402.84	6.67	50.42	1.00	46.05	31.66
706	13,044.71	327.87	8.43	76.86	1.00	80.64	33.53
707	2,894.82	67.26	11.00	81.78	1.00	90.05	0.00
708	7,154.20	118.90	5.75	72.01	1.00	66.22	1.06
709	3,717.76	129.53	49.54	88.71	1.00	69.54	0.00
710	2,365.45	68.21	12.43	86.46	1.00	78.63	14.42
711	7,564.30	183.58	12.77	90.05	1.00	89.87	12.49
712	5,506.60	168.77	12.90	63.94	1.00	72.33	13.52
713	3,766.04	95.45	15.71	79.29	1.00	54.88	2.88
714	15,381.12	686.54	19.19	76.98	1.00	22.70	12.47
715	2,223.76	54.92	12.42	96.36	1.00	56.31	2.20
716	18,164.19	583.24	13.87	67.09	1.00	26.31	4.07
717	6,464.71	195.37	10.27	80.68	1.00	69.00	20.68
718	4,943.12	156.62	10.05	59.69	1.00	95.64	0.00
719	7,466.17	527.26	13.22	50.02	1.00	86.17	11.49
720	5,149.21	119.31	7.90	62.94	1.00	88.45	6.99
721	7,177.63	270.44	8.94	60.95	1.00	89.84	38.61
722	5,849.21	97.16	7.62	65.69	1.00	45.93	4.89
723	7,758.05	533.17	12.94	50.62	1.00	96.22	0.00
724	58,748.60	4,216.35	10.62	46.41	1.00	95.78	18.57
725	4,040.57	237.40	14.39	60.74	1.00	93.60	0.00
726	6,262.62	192.54	10.15	45.94	1.00	35.17	0.00

727	16,222.87	426.81	13.72	48.82	1.00	31.24	11.71
728	6,731.30	120.54	14.63	66.93	1.00	19.44	1.54
729	2,213.47	131.73	16.11	63.63	1.00	98.48	0.00
730	12,176.76	295.20	12.94	58.10	1.00	56.88	30.09
731	18,840.15	417.72	9.42	86.01	1.00	85.82	12.66
732	11,682.63	300.60	22.84	84.98	1.00	57.29	0.00
733	8,734.66	317.96	8.47	61.12	1.00	97.26	51.00
734	32,535.01	711.23	10.18	86.14	1.00	69.82	6.01
735	54,135.18	3,933.92	10.78	37.42	1.00	90.15	29.31
736	3,565.52	208.14	9.43	76.30	1.00	99.87	4.25
737	14,508.45	357.48	22.12	85.72	1.00	68.52	0.00
738	11,340.36	823.79	13.79	46.94	1.00	98.39	0.00
739	21,398.50	462.21	9.91	71.00	1.00	29.48	2.13
740	2,646.17	224.14	7.75	51.49	1.00	96.60	0.00
741	2,362.95	190.56	6.88	51.51	1.00	95.26	19.02
742	11,420.70	783.49	12.10	57.23	1.00	98.80	23.36
743	6,801.46	140.89	22.44	80.02	1.00	58.20	0.00
744	34,756.56	877.42	12.55	52.02	1.00	24.06	10.13
745	10,135.41	314.55	8.88	58.13	1.00	82.70	49.26
746	16,408.13	324.97	10.59	56.15	1.00	37.55	0.00
747	25,132.03	1,920.82	8.68	57.38	1.00	94.35	4.52
748	24,460.90	649.98	23.15	53.37	1.00	34.24	19.16
749	31,625.25	639.41	10.43	67.32	1.00	13.63	0.00
750	5,479.06	163.67	5.42	77.03	1.00	96.28	30.65
751	29,069.93	551.69	11.33	46.67	1.00	27.80	5.54
752	10,406.72	680.29	1.08	89.00	1.00	99.87	74.20
753	5,532.82	410.37	8.98	71.74	1.00	89.34	0.00
754	29,433.09	633.06	20.31	66.82	1.00	33.61	0.00
755	2,400.59	233.76	5.90	47.99	1.00	95.53	21.70
756	29,872.32	636.08	20.01	68.62	1.00	44.86	0.00
757	31,036.37	648.54	11.73	63.69	1.00	10.66	0.00
758	4,057.64	87.70	22.41	76.68	1.00	57.82	2.01
759	8,785.14	159.75	21.01	63.86	1.00	8.31	0.00
760	14,935.55	257.25	11.39	76.89	1.00	82.27	2.39
761	24,394.66	414.10	13.88	80.89	1.00	31.64	0.00
762	5,299.31	94.14	17.21	57.28	1.00	16.55	0.00
763	38,380.24	967.11	13.29	66.50	1.00	15.30	29.04
764	33,515.18	822.64	21.46	73.95	1.00	11.94	3.19
765	17,781.43	515.27	6.21	73.79	1.00	47.12	26.94
766	17,191.65	395.71	16.23	38.91	1.00	34.38	45.14
767	12,974.89	205.37	13.48	78.30	1.00	40.15	0.00
768	44,749.00	1,255.82	8.69	54.63	1.00	32.00	38.98
769	7,251.90	174.15	25.84	60.17	1.00	9.14	0.00
770	4,963.56	232.53	3.56	66.61	1.00	93.01	36.00
771	21,651.15	579.66	8.32	58.02	1.00	75.27	6.05
772	65,281.67	1,409.14	19.68	62.08	1.00	31.62	2.09
773	87,518.19	2,261.32	8.58	57.29	1.00	37.55	29.57
774	13,805.63	865.87	9.48	70.47	1.00	91.60	6.29
775	151,607.52	3,749.57	10.57	53.96	1.00	28.62	29.04
776	41,433.12	2,645.78	6.68	81.28	1.00	99.23	69.72
777	17,785.90	430.07	6.52	33.67	1.00	21.64	56.45
778	2,499.00	72.59	5.18	62.79	1.00	43.46	17.77
779	4,846.18	112.41	7.05	52.61	1.00	78.70	41.29
780	3,156.45	106.57	4.44	42.57	1.00	52.90	50.55
781	5,172.23	129.46	18.70	73.37	1.00	10.14	6.67
782	11,577.80	302.00	10.96	52.13	1.00	24.45	27.65
783	8,666.40	207.14	33.21	62.05	1.00	77.56	0.00
784	16,056.51	362.02	26.42	60.06	1.00	16.76	2.16
785	16,773.66	301.92	11.76	55.49	1.00	61.19	1.19
786	9,782.81	281.73	4.76	65.44	1.00	27.22	23.93
787	19,261.42	475.64	21.13	57.38	1.00	35.65	43.03

788	5,683.15	79.82	3.11	56.86	1.00	0.00	0.00
789	4,579.29	77.58	9.38	60.69	1.00	71.15	4.60
790	8,449.83	219.56	33.83	60.89	1.00	22.82	1.75
791	29,511.52	582.39	16.64	48.10	1.00	26.48	1.92
792	17,678.56	512.53	11.35	53.04	1.00	38.13	24.41
793	7,730.16	150.92	20.06	67.46	1.00	51.74	0.00
794	2,395.65	69.48	12.72	37.06	1.00	29.19	21.83
795	7,563.05	266.11	4.59	84.02	1.00	71.24	50.80
796	3,198.44	64.49	10.29	66.99	1.00	67.97	22.24
797	6,372.36	158.57	20.38	57.01	1.00	35.47	30.04
798	11,640.89	238.55	11.20	61.40	1.00	64.89	21.37
799	7,106.62	226.00	10.89	76.96	1.00	74.25	30.05
800	3,612.88	73.92	14.71	62.47	1.00	69.77	4.34
801	6,987.84	139.35	11.23	70.51	1.00	70.46	13.19
802	3,804.35	73.06	9.35	57.13	1.00	34.34	2.16
803	4,700.38	113.07	30.56	55.57	1.00	71.62	23.53
804	21,557.26	382.63	15.61	44.49	1.00	42.08	0.00
805	12,091.13	251.03	9.29	79.17	1.00	83.14	17.24
806	20,736.88	452.92	16.28	49.38	1.00	28.65	8.37
807	2,843.70	96.80	5.47	68.84	1.00	80.96	25.66
808	29,560.70	679.57	20.06	56.55	1.00	25.17	9.38
809	6,374.62	158.15	4.68	52.65	1.00	66.45	0.00
810	10,574.22	250.94	15.44	55.15	1.00	10.87	12.77
811	45,991.93	901.46	11.42	55.90	1.00	17.61	16.14
812	10,265.85	176.88	10.69	53.37	1.00	25.31	4.22
813	27,881.18	503.30	12.15	63.66	1.00	9.27	0.00
814	34,557.58	665.76	9.47	57.60	1.00	43.47	5.42
815	13,121.97	202.33	7.79	54.62	1.00	70.67	0.00
816	4,872.35	23.72	2.95	65.30	1.00	67.74	0.00
817	8,557.17	160.99	14.80	41.87	1.00	47.27	0.00
818	164,750.69	4,292.67	12.09	57.86	1.00	38.61	33.14
819	14,944.49	293.40	14.15	46.20	1.00	39.65	0.00
820	60,473.69	1,241.91	11.19	49.09	1.00	17.52	24.38
821	3,512.23	86.79	19.56	67.17	1.00	28.41	0.00
822	4,462.53	94.97	19.98	80.36	1.00	77.39	3.92
823	12,025.34	188.92	8.47	67.39	1.00	76.35	0.00
824	160,696.84	4,136.90	11.19	59.83	1.00	12.70	43.49
825	83,120.99	1,699.02	23.57	53.41	1.00	30.10	23.83
826	7,234.19	135.44	8.84	85.06	1.00	83.21	20.11
827	9,259.85	202.51	14.94	56.75	1.00	60.54	18.25
828	2,507.39	51.36	2.51	86.65	1.00	90.67	21.98
829	6,455.96	137.16	11.14	56.16	1.00	13.49	5.50
830	7,476.52	157.54	14.21	94.32	1.00	97.35	8.73
831	9,160.27	199.89	10.91	89.03	1.00	91.82	7.00
832	6,206.09	134.91	4.53	76.66	1.00	34.75	33.23
833	5,476.79	112.26	11.45	70.96	1.00	67.73	31.55
834	9,110.89	156.69	4.47	67.09	1.00	90.26	3.95
835	76,522.90	2,261.60	10.94	52.21	1.00	38.85	57.41
836	11,716.09	262.44	9.18	72.61	1.00	69.30	19.23
837	12,945.02	322.39	8.57	74.89	1.00	65.66	33.81
838	7,412.29	186.64	29.10	61.52	1.00	27.70	12.74
839	13,528.93	243.92	18.93	53.76	1.00	28.00	16.11
840	9,574.39	228.65	8.44	71.22	1.00	18.75	28.52
841	376,526.70	9,000.07	12.98	51.75	1.00	21.58	36.98
842	89,809.05	2,562.11	8.40	56.89	1.00	22.75	28.25
843	9,288.33	175.67	9.02	70.90	1.00	84.12	29.94
844	3,774.41	96.14	13.19	55.00	1.00	74.54	40.29
845	2,717.70	72.29	9.44	74.56	1.00	70.42	23.22
846	7,218.94	119.95	10.31	81.27	1.00	78.70	0.00
847	33,255.99	945.36	11.62	60.86	1.00	44.71	36.67
848	271,132.00	6,001.12	14.89	65.10	1.00	52.92	58.08

849	2,980.88	102.93	7.80	71.13	1.00	78.51	35.48
850	13,458.48	261.86	12.25	68.92	1.00	83.96	17.51
851	3,882.41	138.30	23.14	79.76	1.00	87.80	35.54
852	6,564.96	165.50	11.68	76.25	1.00	81.24	34.07
853	5,875.94	158.95	8.39	54.36	1.00	70.01	17.63
854	25,641.05	881.08	10.57	58.76	1.00	57.07	47.05
855	6,340.24	146.30	8.63	66.25	1.00	2.64	2.99
856	258,198.37	5,733.08	14.71	56.06	1.00	41.50	26.79
857	75,444.70	2,245.43	8.49	56.74	1.00	13.16	34.69
858	8,628.62	180.85	6.77	59.15	1.00	24.95	13.01
859	7,160.36	171.61	13.21	62.50	1.00	31.49	40.19
860	8,564.62	157.96	7.53	63.33	1.00	91.50	3.19
861	4,747.71	159.99	6.87	72.31	1.00	66.54	28.66
862	10,665.97	213.24	6.25	50.28	1.00	20.65	22.70
863	5,357.17	250.67	9.67	80.38	1.00	95.15	73.06
864	2,923.12	86.33	17.88	78.74	1.00	12.01	3.47
865	4,253.22	94.73	15.35	65.34	1.00	56.78	18.27
866	46,574.18	1,591.10	8.41	54.59	1.00	15.34	33.66
867	13,680.02	255.09	10.96	39.59	1.00	53.25	1.35
868	8,216.77	165.03	7.14	73.42	1.00	88.94	17.80
869	5,080.36	97.61	14.85	71.65	1.00	88.83	8.47
870	4,269.51	95.15	13.25	72.71	1.00	24.27	9.07
871	314,096.65	6,989.96	9.37	54.57	1.00	52.97	79.14
872	5,241.53	102.67	11.65	55.43	1.00	95.63	12.54
873	2,660.65	149.89	4.02	89.32	1.00	92.25	75.35
874	37,962.49	1,306.66	8.71	56.28	1.00	30.21	48.84
875	87,517.93	2,145.54	11.63	55.45	1.00	26.14	38.65
876	12,535.99	468.92	8.80	54.75	1.00	29.42	41.12
877	10,045.28	205.14	15.39	55.71	1.00	74.61	13.16
878	7,529.56	327.95	5.32	83.30	1.00	90.57	31.68
879	2,468.65	58.72	19.16	79.53	1.00	95.15	12.19
880	4,927.09	122.77	10.46	45.78	1.00	47.20	27.58
881	7,068.78	121.13	7.48	80.19	1.00	66.74	1.97
882	29,025.48	907.29	8.61	59.45	1.00	18.23	8.12
883	5,833.30	144.58	7.61	67.12	1.00	86.72	39.48
884	5,285.45	166.48	8.78	67.63	1.00	55.75	10.80
885	3,216.52	186.97	2.81	68.27	1.00	85.73	41.42
886	102,029.16	2,261.98	16.30	61.49	1.00	47.39	39.33
887	13,067.24	370.06	12.33	61.86	1.00	40.76	30.34
888	29,712.64	732.17	12.75	51.72	1.00	48.94	4.24
889	3,805.82	78.88	11.57	76.14	1.00	90.12	13.36
890	1,010.35	43.60	3.94	83.58	1.00	87.47	42.24
891	4,502.21	91.61	6.53	76.45	1.00	44.18	11.44
892	10,369.06	287.93	8.61	62.53	1.00	64.94	6.08
893	269,107.73	5,972.81	9.40	57.64	1.00	77.79	41.47
894	18,458.65	434.71	8.57	53.32	1.00	81.30	44.77
895	17,925.11	438.28	10.72	44.84	1.00	74.69	31.17
896	8,945.46	235.65	15.89	69.32	1.00	51.64	5.39
897	4,314.85	95.72	14.21	64.67	1.00	90.15	31.08
898	5,606.29	144.46	14.47	66.72	1.00	45.82	28.26
899	5,649.22	270.29	9.49	60.13	1.00	79.04	41.25
900	22,617.04	609.05	8.07	64.52	1.00	49.54	20.64
901	1,793.31	77.14	11.48	80.23	1.00	89.11	38.12
902	1,621.97	55.43	9.39	80.09	1.00	83.16	28.02
903	10,148.15	206.90	12.86	67.80	1.00	86.54	16.58
904	4,025.59	184.93	11.07	85.83	1.00	80.14	6.20
905	45,240.55	1,064.89	13.51	57.23	1.00	34.89	5.10
906	18,683.33	506.54	13.55	64.04	1.00	63.05	37.02
907	8,488.75	217.05	8.52	76.80	1.00	74.24	33.87
908	1,648.70	97.51	8.45	85.60	1.00	91.96	53.22
909	5,342.26	124.94	7.97	47.03	1.00	87.07	31.63

910	2,611.13	162.15	2.30	83.58	1.00	97.18	64.28
911	24,610.15	604.76	11.33	49.69	1.00	57.52	2.30
912	3,224.87	52.06	12.77	86.73	1.00	60.08	0.00
913	10,344.80	281.27	12.29	74.77	1.00	69.99	35.36
914	9,337.20	227.90	8.36	74.92	1.00	66.87	37.35
915	5,707.05	136.36	8.03	78.69	1.00	74.81	2.11
916	1,280.15	48.30	3.99	72.84	1.00	29.34	24.37
917	1,093.28	37.90	3.23	49.53	1.00	90.78	32.75
918	814,038.40	19,384.30	16.21	58.14	1.00	22.94	49.20
919	4,922.10	97.03	23.50	69.91	1.00	0.25	0.00
920	286,694.44	6,683.90	15.71	37.04	1.00	9.23	18.67
921	9,822.09	183.70	21.11	61.46	1.00	47.55	7.07
922	60,115.06	1,307.05	14.69	60.69	1.00	17.07	6.04
923	6,425.75	136.37	12.64	67.27	1.00	23.83	3.69
924	10,327.45	196.25	13.38	66.62	1.00	20.16	2.64
925	31,076.52	776.50	8.56	54.23	1.00	22.75	30.62
926	5,628.59	148.20	12.07	52.39	1.00	24.81	18.54
927	6,287.39	190.85	18.39	56.62	1.00	59.06	30.18
928	3,171.91	251.23	2.80	69.95	1.00	79.48	44.62
929	4,369.85	254.50	7.98	54.95	1.00	92.11	12.03
930	14,127.98	951.37	11.85	67.07	1.00	95.59	0.00
931	3,394.65	66.34	10.55	80.19	1.00	96.15	3.98
932	5,716.38	75.76	10.48	92.41	1.00	100.00	24.01
933	95,701.85	8,061.88	8.85	30.99	1.00	58.54	39.64
934	23,968.47	3,345.35	8.51	42.21	1.00	40.75	61.84
935	3,660.00	384.85	8.46	38.53	1.00	56.24	61.56
936	3,759.59	384.52	8.34	40.46	1.00	64.54	59.11
937	3,565.83	428.53	6.99	38.14	1.00	58.14	31.49
938	4,124.20	350.12	10.45	53.53	1.00	81.21	19.76
939	4,613.96	516.96	13.92	53.53	1.00	81.84	41.80
940	1,817.52	261.56	3.89	30.88	1.00	74.84	65.73
941	10,224.81	897.69	4.63	52.10	1.00	82.99	11.69
942	7,279.91	902.21	2.94	70.50	1.00	90.99	52.81
943	2,733.66	271.30	7.54	69.86	0.00	93.58	66.89
944	1,878.78	181.41	3.46	54.92	0.00	82.37	51.05
945	43,833.19	2,011.53	14.48	24.16	1.00	31.60	23.40
946	7,906.72	536.13	4.34	22.72	1.00	18.62	30.81
947	11,869.65	1,222.51	9.71	36.81	1.00	57.46	50.71
948	12,782.58	516.97	8.29	52.68	0.00	44.19	7.80
949	80,979.69	7,832.94	9.72	28.44	1.00	36.19	31.83
950	25,065.70	1,534.68	8.96	26.52	1.00	54.90	25.00
951	31,362.70	4,406.52	8.38	24.73	1.00	36.06	35.16
952	1,645.56	224.99	6.26	61.52	0.00	76.13	35.73
953	12,901.16	1,022.34	10.39	26.02	1.00	27.82	40.68
954	17,593.11	1,053.19	7.59	10.41	1.00	13.05	17.90
955	3,612.96	800.86	7.02	42.63	1.00	57.03	19.79
956	12,856.12	1,037.28	8.91	52.76	0.00	66.59	34.01
957	10,528.08	620.47	8.90	42.27	0.00	72.41	39.36
958	7,389.07	419.81	7.01	45.39	0.00	70.38	25.62
959	12,084.75	1,126.01	13.09	28.97	1.00	51.84	28.44
960	15,894.34	1,234.79	14.76	29.18	1.00	13.58	44.14
961	26,636.03	1,316.79	9.09	9.52	1.00	19.04	18.67
962	77,380.19	4,454.05	18.62	7.79	1.00	9.60	19.06
963	5,333.27	423.01	6.28	34.94	1.00	16.84	38.82
964	27,337.42	2,181.17	20.55	13.93	1.00	9.36	45.49

ed
